# Assessing Molecular Contacts Using Atom Environments Described by Ranked Lists

**DOI:** 10.1021/acs.jcim.6c00694

**Published:** 2026-07-16

**Authors:** Loic Dreano, Ashenafi Legehar, Mael Briand, Kuura Variskallio, Alexandre Borrel, Henri Xhaard

**Affiliations:** † Drug Research Program, Division of Pharmaceutical Chemistry and Technology, Faculty of Pharmacy, 3835 University of Helsinki, Helsinki FI-00014, Finland; ‡ 673729 Sciome LLC, Research Triangle Park, North Carolina 27713, United States

## Abstract

We investigate the representation of atomic molecular environments as a list of the atoms neighboring an origin ranked by distance. We show that contact densities collected for origin-neighbor pairs of set types have regular and specific distributions when stratified by ranks. This is used to build a scoring function assessing the fitness of individual atoms with respect to their environment (FS, fitness score), as well as to characterize the contact preferences (CP) for pairs of atoms. Tested against the 3DRobot data set, the FS score was found to be highly discriminative of decoys vs native X-ray. The code is freely available on GitHub (https://github.com/DreanoLoic/Fitness_score.git).

## Graphical abstract

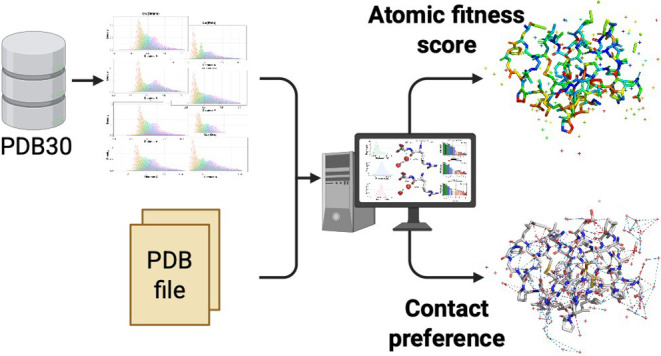

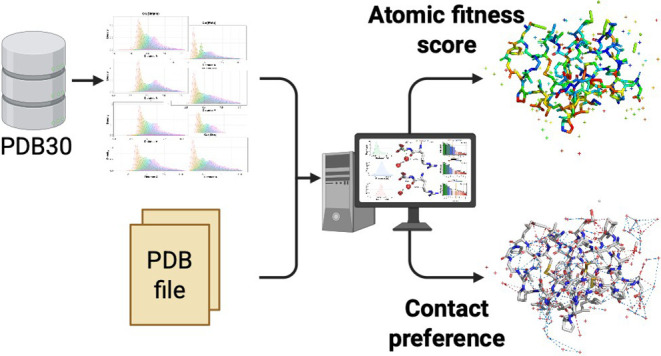

## Introduction

Knowledge-based scoring functions are widely used in docking software such as GOLD,^1,2^ FlexX,^3^ Glide,^4^ and Dock,^5^ where they are used to predict poses, rank compounds and predict binding affinities.^6–9^ These functions use knowledge-based potentials to model molecular interactions, which are derived from densities of presence collected for pairs of atoms normalized to a reference state. Knowledge-based functions can capture a wide range of molecular interactions, including uncommon interactions often poorly described by standard force fields.^10–12^ When building such functions, a way to collect solid statistics is, for example, to focus on line-of-sight interactions between atoms.^13,14^ It is moreover important to account for solvation, and solvent exposure has been used to modulate knowledge-based scoring functions, as in the Astex Statistical Potential and others.^15,16^ A surface-area function such as the one used in PLIff, computed using a Voronoi tessellation algorithm, allows focusing on direct contacts while accounting for solvation.^17^ Atomic distributions have been modeled using different methods, including, for example, Gaussian mixtures.^18^

Another way to apprehend molecular interactions is through the propensity of a pair of atoms to be in contact. Small networks present a valuable tool to represent and visualize molecular contacts.^19^ This approach has been developed by Kuhn and co-workers based on the seminal work by Taylor^13^ with the ratio of frequencies (*R*_F_) score, which is based on the propensity of atomic surfaces to interact and takes into account angular preferences for predefined SMART fragments.^13,14,20^

In previous work, we have conducted two large data-mining studies of the PDB; one in which we characterize the environments around ion pairs in both proteins and ligands;^21^ and another where we study structural isosteres of phosphate groups.^22^ For ion pairs, we characterized contact preferences through a contingency analysis.^21^ This early work was conducted using scripts, but since then we have developed a relational database to explore molecular contacts.^23^

Here, we introduce a conceptual framework that describes the environment of a given origin atom by the list of its neighboring atoms ranked by distance. We present a key observation, in which the distribution of the neighbors collected over multiple pairs is very regular when stratified by their ranks in the list. This allows us to build a *fitness score* (*FS*) for an individual atom, derived from the distances of it*k* first neighbors and accounting for the types of the atoms involved as well as their ranks in the neighbor list. Furthermore, we estimate atoms’ pairwise *contact preference* (*CP*), defined as the ratio of the observed frequency of encountering a given neighbor type at a given rank to the background frequency of that type at the same rank. Overall, these scores share similarities with, respectively, knowledge-based scoring functions^10–12,15–18^ and the *R*_F_ score developed by Taylor as well as Kuhn and co-workers.^13,14,20^

## Experimental Section

Data collection and analysis were performed using Python scripts, R for statistical analysis, and a PostgreSQL database that has been described elsewhere.^23^ The database was created and populated with a Python script using the SQLAlchemy (v.2.0.13) package.^24^ Data were handled using NumPy (v.1.20.2) objects (lists of distances, identifiers, and other information).^25^ PyMOL (v.2.5.4) was used for visualization.^26^ Distance calculations were made using Sklearn (v.0.24.2) KDTree.^27^ R packages (RPostgreSQL, ggplot2, ggrepel, plyr, ca) were used for statistical analysis, density calculation, and plots.^28^

We used 11 custom atom types defined by aggregating the 119 PDB atom denominations (Supporting Information 1). Seven of these types encoded protein atoms, and three encoded ligand heteroatoms, metals, and water molecules, respectively. Protein hydrogen atoms were assigned as an 11th type but were not considered in this study. Additionally, a twelfth “empty” type was defined to handle missing neighbors during environment construction.

We represented the molecular environment of an atom as a distance-ranked list of its neighboring atoms (Figure 1). Throughout the manuscript, *i* denotes an origin atom (the atom whose environment is being scored) and *j* denotes one of its neighbors. For each origin atom *i* of type *T*_*i*_, we defined the ranked environment of size *k*, denoted *Env*_*k*_(i), as the ordered set of the *k* nonbonded neighbors of *i*, sorted by increasing distance to *i*. To each neighbor *j* ∈ *Env*_*k*_(i) we attached three quantities: its type *T*_*j*_, the distance *d*_*ij*_ between *i* and *j*, and its rank *r*_*j*_, corresponding to the position 1, 2, ..., *k* of *j* inside *Env*_*k*_(i). Neighbors *j* of an origin *i* were defined as all atoms within 10 Å of *i*, a distance beyond which no significant nonbonded interactions occur. We further applied two types of filters in order to avoid considering atoms whose presence is only a result of chemical bonding. The first filter, *self*, excluded from *Env*_*k*_(i) all atoms from the residue associated with *i* as well as those from the residues immediately preceding and following it in the protein chain. The second filter, *primary contacts*, retained in *Env*_*k*_(i) only the closest atom from each interacting residue, thus omitting atoms that might be found only due to covalent linkage to the atom forming the primary contact. In this manuscript, the *self* filter was always applied whereas, different uses of the *primary contact* filter were investigated.

**Figure 1:**
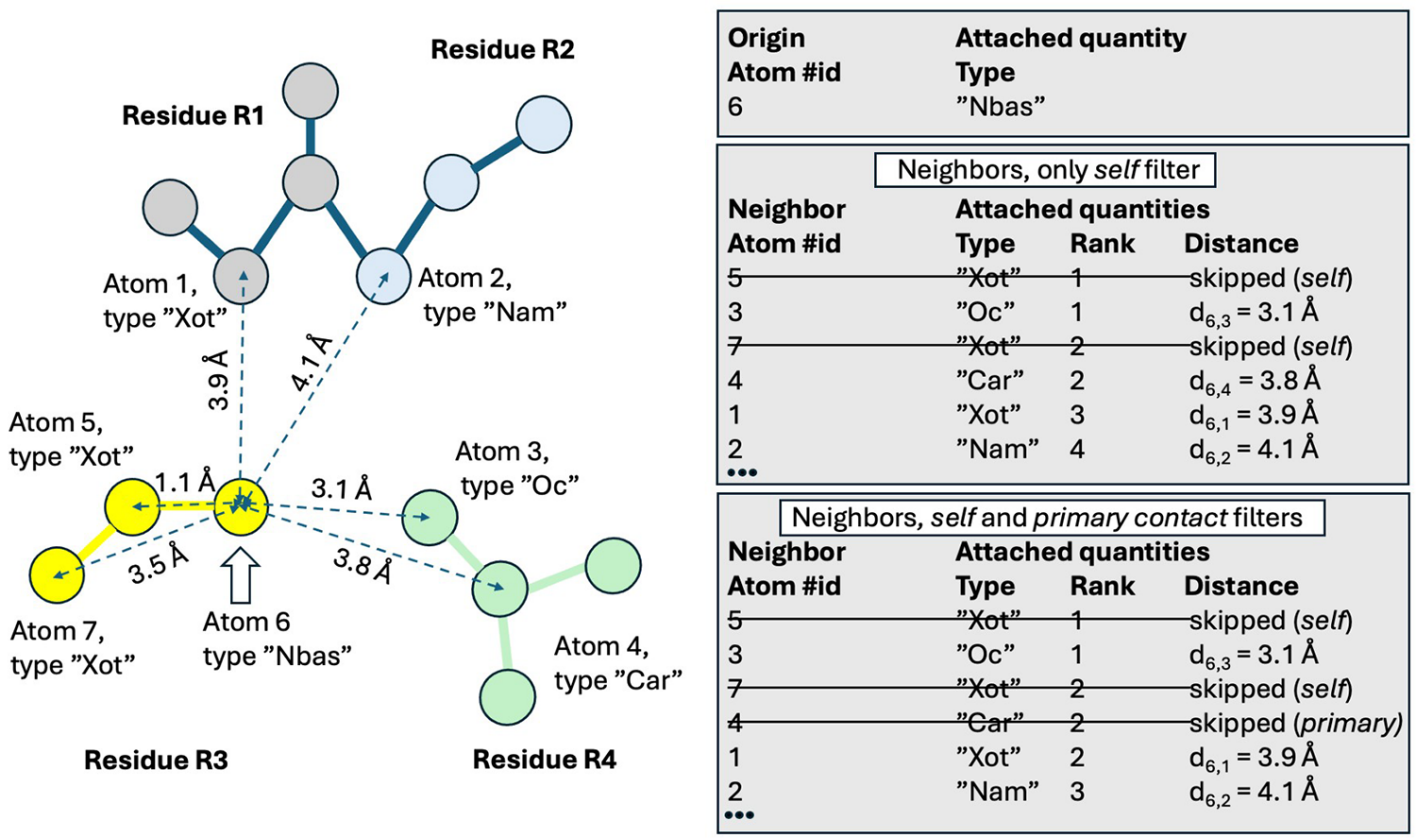
Schematics illustration of the construction of the neighbor lists. The schematics portray seven labeled atoms (1–7) belonging to four amino acid residues (R1 (gray), R2 (light blue), R3 (yellow) and R4 (light green)) used to compute the *self* filter. The closest atomic distances (Å) from origin atom 6, used to construct the ranked lists of neighbors, are shown by dashed lines. Neighbor lists can be constructed considering only the *self* filter (middle-right box) and considering both the *self* and *primary contact* filters (bottom-right box).

In the last part of this manuscript, we investigated the atom types of the neighbors in *Env*_*k*_(i) abstracted into a *k*-tuple, where each position corresponds to a rank in the neighbor list. Two variants were considered: tuples preserving the rank order of *Env*_*k*_(i), and unordered tuples where only the set of neighbor types is retained, irrespective of their position (e.g., Oow-Nbas-Car and Car-Oow-Nbas would belong to the same unordered tuple). These k-tuples are not used in constructing the fitness score *FS* and the contact preference *CP* described below.

We used, for deriving statistics on molecular environments, a set of 22,930 unique representative structures (mean resolution 2.27 Å; standard deviation 0.66 Å) solved by X-ray crystallography, referred to as PDB30. This set was downloaded from the PDB (accessed January 2018) and is based on a BLAST search clustering (27,330 clusters sharing over 30% sequence identity), where for each cluster a representative structure with the highest resolution and more than 20 amino acids was selected.

The molecular environment of every atom in the PDB30 data set was calculated and stored in the *contact_matrix* table of the custom PostgreSQL database.^23^ For each origin atom, the lists of the ten closest neighbors for both full- and primary-contact configurations (i.e., with or without the *primary contact* filter) were stored in the *environment* table. If an atom had fewer than ten neighbors within the 10 Å search sphere, the remaining positions in these lists were filled with the “empty” type.

For all 1000 possible triplets (10 origin types × 10 neighbor types × 10 ranks) of origin type *T*_*i*_, neighbor type *T*_*j*_, (a pair hereafter noted *T*_*i*_(*T*_*j*_)) and rank *r*_*j*_ ∈ {1, ..., 10}, we precomputed a reference distance density ρ_*T*_*i*_,*T*_*j*_,*r*_(*d*) from PDB30. Using R, we discretized each density into 512 distance points–which allows a very high resolution since each 1.0 Å of density is represented by ∼50 values–and stored the resulting values as tabulated files (distance, density). Each density describes the probability of observing a neighbor of type *T*_*j*_ at distance *d* from an origin of type *T*_*i*_ when that neighbor occurs at rank *r* in PDB30.

We used the distance densities computed from the PDB30 set to construct a knowledge-based scoring function, the *fitness score* (*FS*), which evaluates how well the local environment of an atom agrees with these reference density distributions. For each neighbor *j*∈*Env*_*k*_(i), we calculated a contribution score *S*_*T*_*i*_,*T*_*j*_,*r*_*j*__(*d*_*ij*_), abbreviated *S*_*ij*_, by comparing the observed distance *d*_*ij*_ to the corresponding reference distance density distribution ρ_*T*_*i*_,*T*_*j*_,*r*_*j*__(d) associated with the (origin type, neighbor type, rank) triplet (*T*_*i*_,*T*_*j*_,*r*_*j*_). The contribution score was obtained in relation to the maximum of that normalized density distribution, such that *s*_*ij*_ = 1 corresponded to an optimal (most probable) distance

1 $$S_{T_{i},T_{j},r_{j}}\left(d_{\mathrm{ij}}\right)\equiv S_{\mathrm{ij}}=\frac{\rho_{T_{i},T_{j},r_{j}}\left(d_{\mathrm{ij}}\right)}{\max_{d}\;\rho_{T_{i},T_{j},r_{j}}\left(d\right)}$$

By construction (eq 1), *s*_*ij*_ lies in [0, 1] and equals 1 when *d*_*ij*_ coincides with the mode of the reference density. The atomic fitness score *FS*_atom_^*k*^(*i*) of origin *i* is the arithmetic mean of the contribution score of its *k* ranked neighbors

2 $${\mathrm{FS}}_{\mathrm{atom}}^{k}\left(i\right)=\frac{1}{k}\sum_{j\in \mathrm{En}v_{k}\;\left(i\right)}S_{\mathrm{ij}}$$

By construction (eq 2), an exception was defined where there were no neighbors at a given rank, the score *s*_*ij*_ of this position was arbitrarily given the value of 0.5, which was chosen due to a limited impact on the score. This case was not observed at the first 3 ranks studied.

A fitness score *FS*_structure_^*k*^(*Q*) of a structure *Q* was obtained by averaging *FS*_atom_^*k*^(*i*) over all origin atoms in *Q*. Formally, denoting *P*(*Q*) the set of origin atoms of *Q*

3 $${\mathrm{FS}}_{\mathrm{structure}}^{k}\left(Q\right)=\frac{1}{\left|P\left(Q\right)\right|}\sum_{i\in P\left(Q\right)}FS_{\mathrm{atom}}^{k}\left(i\right)$$

The performance of the *FS* score was evaluated using two independent data sets. First, the 3DRobot data set, composed of 200 native structures associated each to 300 decoys generated with diverse levels of similarity,^29^ was used to evaluate the ability of the fitness score to discriminate between native and non-native conformations. The 3DRobot data set provides for each decoy a root-mean-square deviation (RMSD) to native, which was used as such for analysis. We used available scripts from the VoroMQA,^30^ 3DCNN-MQA,^31^ Discrete Optimized Protein Energy (DOPE),^32^ and RWPlus^33^ to score the 3DRobot set. To complete the analysis, solvent accessibility was computed using MODELLER.^34^ Second, a data set of 359 diverse protein structures solved by X-ray crystallography with a resolution lower than 1.0 Å was used to examine how the fitness score relates to structural descriptors such as B-factors and solvent accessibility, as well as to analyze its behavior on water molecules, which are well resolved at these of resolutions.

Building upon the same molecular environment framework, we defined the *contact preference CP*, which evaluates the ratio of the frequencies of observing and expecting a pair of atoms characterized by their types, taking the ranks in the neighbor list into account. For each origin atom of type *T*_*i*_, and each neighbor type *T*_*j*_ observed at rank *r*_*j*_ ∈ {1, ..., *k*}, we defined the observed frequency *f*_obs(*T*_*i*_,*T*_*j*_,*r*_*j*_)_ as the proportion of neighbors of type *T*_*j*_ found at rank *r*_*j*_ around origins of type *T*_*i*_. We also defined the expected frequency *f*_exp(*T*_*j*_,*r*_*j*_)_ as the background proportion of neighbors of type *T*_*j*_ found at the rank *r*_*j*_ across all atom types. The contact preference ratio is then expressed as

4 $$\mathrm{CP}\left(T_{i},T_{j},r\right)=\frac{f_{\mathrm{obs}\left(T_{i},T_{j},r_{j}\right)}}{f_{\exp\left({\cdot ,T}_{j},r_{j}\right)}}$$

A ratio *CP* > 1 indicates that the pair (*T*_*i*_, *T*_*j*_) occurs more frequently than expected at that rank, whereas *CP* < 1 denotes an underrepresented contact. Unlike the fitness score *FS*, the contact preference *CP* is independent of interatomic distances. Of note, the relationship between pairs is nonreciprocal; meaning the rank of *T*_*j*_ as a neighbor of *T*_*i*_ does not necessarily match the rank of *T*_*i*_ as a neighbor of *T*_*j*_.

Statistical analyses were further performed using a simple correspondence analysis in R to explore relationships between atom types and their positions in the ranked neighbor lists. For each origin atom type, a contingency table was constructed with neighbor atom types as rows and ranks *r*_*j*_ ∈ {1, ...,10} as columns, populated with occurrence counts extracted from the *environment* table. The correspondence analysis decomposes this contingency table into orthogonal dimensions that summarize the association structure between atom types (rows) and ranks (columns) based on occurrence counts (cells), each dimension explaining a fraction of the total information contained in the data table.

## Results

For the purpose of presenting this study, we focus on three atom types: aromatic carbon (Car in the custom types), basic nitrogen (Nbas), and water oxygen (Oow); data on other types are presented as Supporting Information. The number of origin atoms collected for these cases was 4,965,111 for Car, 867,579 for Nbas, and 2,683,524 for Oow.

### The Frequency of Neighbors is Conditioned by Their Rank and Type

For all origins, the frequencies of the observed neighboring atom types vary along their ranks in the neighbor list (Supporting Information 2). For example, for Nbas there is a high proportion of carboxylate oxygens (Oox) and carbonyl groups (Oc) in the first three ranks, which is easily explained since Oox can act as a counterion and Oc as a hydrogen-bond acceptor. Nbas has also empty neighbors at high ranks, a probable consequence of the long side chains of arginine and lysine pointing toward the protein exterior in crystal structures. For the water molecules (Oow), most interactions at close range are polar, either with other water molecules or with polar protein groups.

Overall, at the ranks 3–5, the relative proportion of aliphatic carbons and sulfur atoms (Xot) generally increases. Comparing rank 1 and rank 10 (Supporting Information 2), the profiles are clearly different. At rank 10, the frequencies of neighbors are somewhat similar among the ten origins, except for the ligand atoms query (Hetatm). This reflects that molecular interactions are specific at close range. The “anomaly” for Hetatm might be explained by the inclusion of, for example, lipid–lipid and ligand-cofactor data points in the data set. The frequencies of observing different neighbors at lower ranks are not influenced by the filtering method (*primary contacts* or not) used to define neighbors (Supporting Information 2).

The Figure 2A corresponds to the case of a “classical” way of collecting the densities of pairwise distances of given atom types (some scoring functions may consider only the first closest contact). Strikingly, when accounting for possible ranks in the neighbor list, densities appear to be well stratified (Figure 2B,C) (all densities provided as Supporting Information 3). Indeed, there is a strong order in the position and shape of the peaks when accounting for the ranks in the neighbor list (Figure 2B,2C). The interval between peaks is regular for most of the 1000 densities investigated (Supporting Information 3). The peaks fit well with those expected from molecular interactions. For example, the peak observed around 2.9 Å corresponds well to polar contacts (Nbas­(Oow)), and around 3.8 Å to cation-π contacts (Nbas­(Car)). Most irregularities correspond to ligand heteroatoms (Hetatm) or amide nitrogens (Nam) from side chain and main chain. When applying the *primary contact* filter, a sharper separation of the peaks is observed (Figure 2C, Supporting Information 3), but the shapes of the distributions do not change.

**Figure 2:**
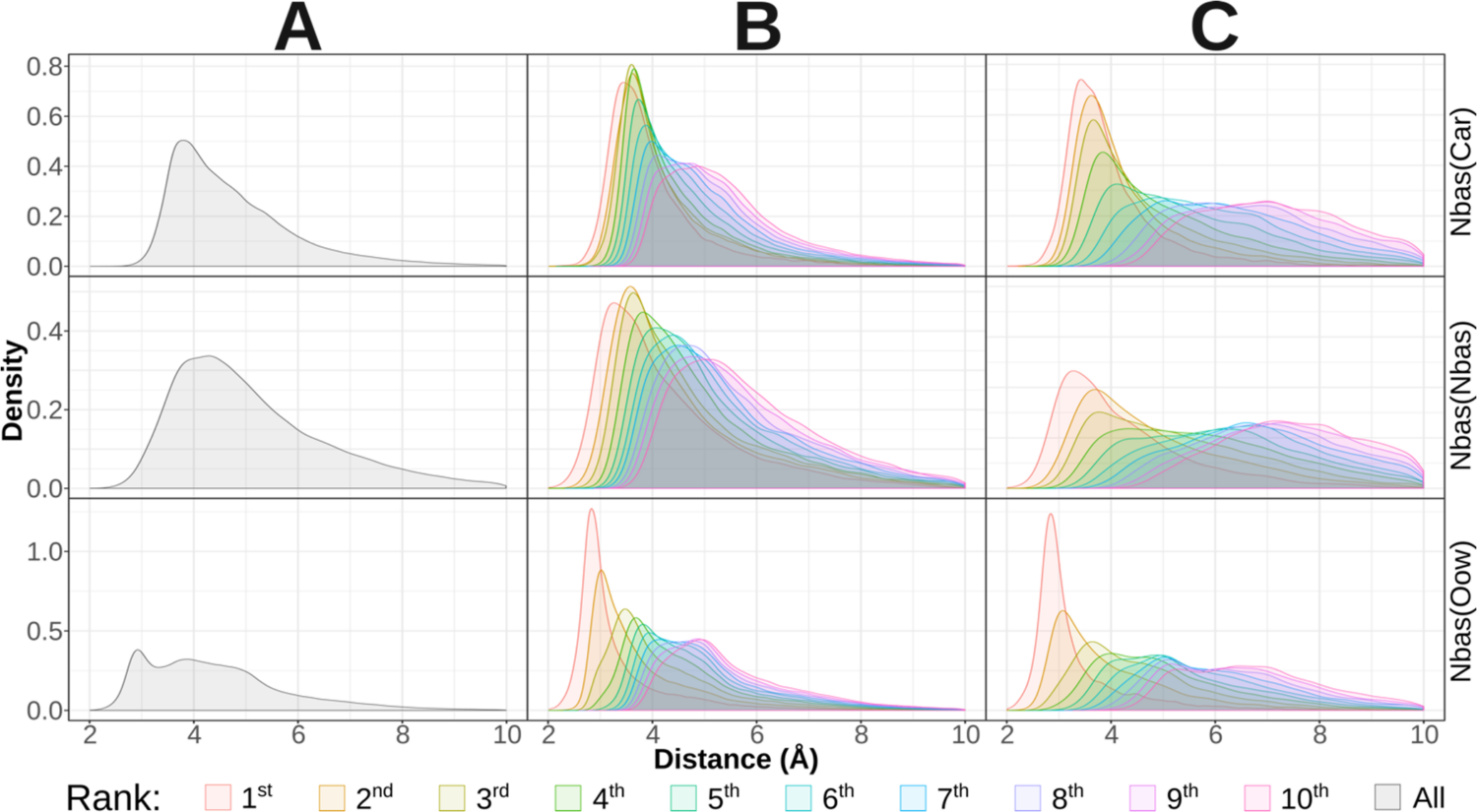
Raw density distributions as a function of distance for the first ten neighbors, exemplified by a Nbas origin and Car (top panels), Nbas (middle) and Oow (bottom) in the PDB30 data set. (A) No stratification based on rank; (B, C) Stratification by neighbor rank. Neighbors are defined as either: (A, B) any atom within 10 Å, or (C) any atoms within 10 Å, but applying the *primary contact* filter.

### The Fitness Score FS

We took advantage of the rank stratification to devise a simple score, the *FS*_atom_^*k*^ score, which assesses the fitness of individual atoms in a three-dimensional molecular structure. For each nonbonded neighbor *j* of origin atom *i*, an individual contribution score *S*_*ij*_ is derived by evaluating the observed distance against the reference density for that specific atom-type pair and rank, normalized to its maximum such that *S*_*ij*_ ∈ [0, 1] (eq 1). *FS*_atom_^*k*^ is then defined as the average of the *k* such contributions (eq 2). For example, using *k* = 3, the three nearest neighbors of water molecule 925 in the crambin crystal structure (PDB code 3NIR) (Figure 3A) are water molecule 924 at rank 1 (Oow, 2.73 Å), the side chain hydroxyl of tyrosine 215 at rank 2 (Oh, 2.75 Å), and the Cγ of arginine 191 at rank 3 (Xot, 4.29 Å), yielding *S*_*ij*_ scores of 1.00, 0.85, and 0.17 respectively, and a final *FS*_atom_^3^(*Oow*_925_) = 0.67.

**Figure 3:**
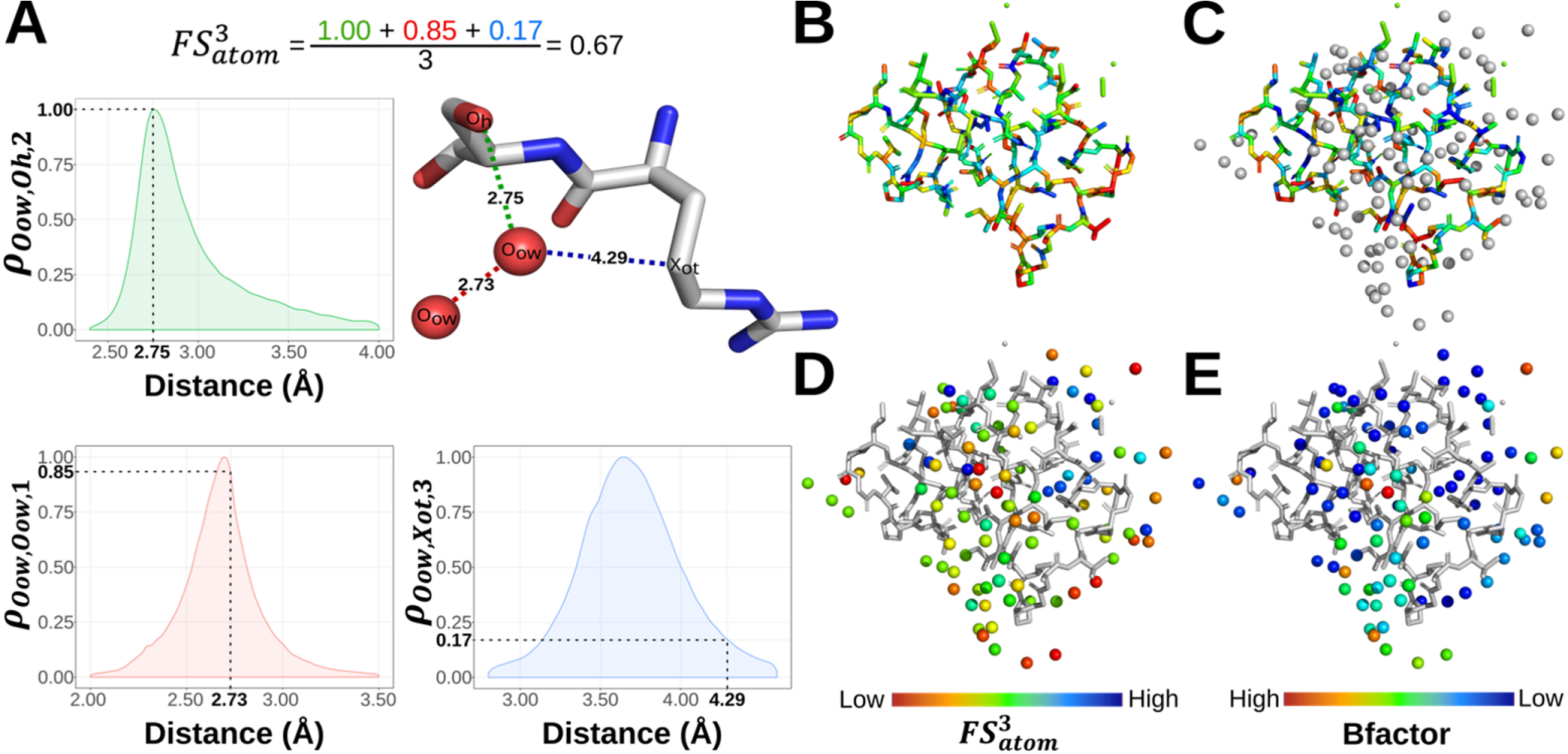
Illustration of the *FS*_atom_^3^ applied to the crambin crystal structure (PDB code 3NIR). (A) Decomposition of *FS*_atom_^3^(*Oow*_925_) into its three contribution scores *S*_*ij*_ (*y*-axis, bold), each derived from a rank- and atom-type-specific normalized reference density as a function of the observed interatomic distance (*x*-axis, bold). (B–D) *FS*_atom_^3^ scores mapped onto the crambin structure using Low–High color scale. (B) Protein atoms scored in the absence of crystallographic water molecules. (C) Protein atoms scored in the presence of crystallographic water molecules. (D) Crystallographic water molecules scored independently. (E) Crystallographic water molecules colored by *B*-factor.

The scripts provided use tables of precomputed densities (discretized into 512 elements, this number being enough to retain a fine grain in all cases) and are therefore fast to execute, 0.5 s to score crambin on a standard computer; (Figure 3B–E). The *FS*_atom_^*k*^ score does not use integration around the maxima, which is justifiable since most peaks are unimodal, and thus integration would likely result in only marginal gain in accuracy at the expense of computational cost.

The *FS*_atom_^*k*^ scoring function can be applied to any atom in a molecular structure, including ligands and water molecules. In general, buried atoms have higher scores than atoms at the surface (Figure 3B). This can be easily explained since the solvent around surface atoms is often not observed by X-ray crystallography. When we accounted for crystallographic water molecules and recalculated the *FS*_atom_^3^ for protein atoms (Figure 3C), the *FS*_atom_^3^ of surface atoms indeed increased. Since *FS*_atom_^*k*^ can be trained on any atom type, including metals and ligand atoms, we tested the approach by scoring water molecules (Figure 3D). As a result, we observed a correspondence where the *FS*_atom_^3^ followed the B-factor (compare Figure 3D,3E), which will be further analyzed in this manuscript.

The method can be applied to calculate a score for a complete protein structure, the *FS*_structure_^*k*^ score, which is the arithmetic mean of all its *FS*_atom_^*k*^ scores (eq 3). We benchmarked the *FS*_structure_^3^ scoring function on a well-studied data set, the 3DRobot data set.^29^ The 3DRobot data set is composed of 200 diverse native X-ray structures each accompanied by a set of 300 decoys of varying molecular similarity, expressed by the RMSD to the reference native structures. The RMSD is calculated by the Zhang group following protein alignment, and concerns only the main chain Cα deviations, not the side chains differences.^29^ We benchmarked four additional scoring functions: DOPE, an atomic distance-dependent statistical potential not dependent on adjustable parameters, based on a reference state corresponding to noninteracting atoms in a homogeneous sphere;^32^ RWplus, a pairwise distance-dependent, atomic statistical potential function, using an ideal random-walk chain as reference state, that incorporates a side-chain orientation-dependent energy term;^33^ VoroMQA, a function that combines statistical potentials with interatomic contact areas, derived from Voronoi tessellation;^30^ and 3DCNN-MQA, which uses a local structure quality evaluation with a deep neural network containing 3-dimensional convolutional neural network layers.^31^ In order to compare the functions, normalizations were conducted. The scores of the native structures were set to 0 and the scores of the decoys oriented so that more negative scores indicate a lesser quality 3D structure. We then conducted two types of normalization, one setting the standard deviation within each native-decoy set as unity, the other setting the standard deviation in the whole set as unity.

As a result, all tested scoring functions were able to discriminate well native X-ray structures (scores set to 0) from their associated decoys (scored less well, and thus obtaining negative scores. The more negative the decoys scores are, the better is the scoring function to discriminate natives from decoys) (Figure 4A and Supporting Information 4). When considering a stringent success criteria, ranking the native structure at the top (position 1 when ranking the scores by decreasing order), this was achieved in 90.3% of the cases for 3DNN, 85.5% for *FS*_structure_^3^, 57.0% for VoroMQA, 31.5% for DOPE, and 21.0% for RWplus. For the DOPE, RWplus, VoroMQA, and 3DCNN-MQA functions, a correspondence between RMSD and similarity to native is apparent (Figure 4 and Supporting Information 4): For decoys very similar to native X-ray structures (low RMSD in 0–2 Å range), normalized scores are close to 0. For these functions, most of the decoy scores are less than one standard deviation away from the native scores (Figure 4F in the 0–2 Å range). In contrast, the *FS*_structure_^3^ score shows a high level of discrimination, with the scores of around 80% of decoys above one standard deviation, even for decoys that have a RMSD close to 0 compared to native (Figure 4B). The dependency on RMSD is much less marked for *FS*_structure_^3^ in contrast to other functions (Figure 4A and Supporting Information 4). Upon further analysis, the lower *FS*_structure_^3^ scores for decoys are due to a drop in their individual *FS*_atom_^3^ scores concerning most of the atoms. Thus, the high decoys/native discrimination is likely driven by the local nature of the *FS*_atom_^3^ scores. Small deviations from optimal distances are in this case heavily penalized, as can be inferred from Figure 3A and eq 1.

**Figure 4:**
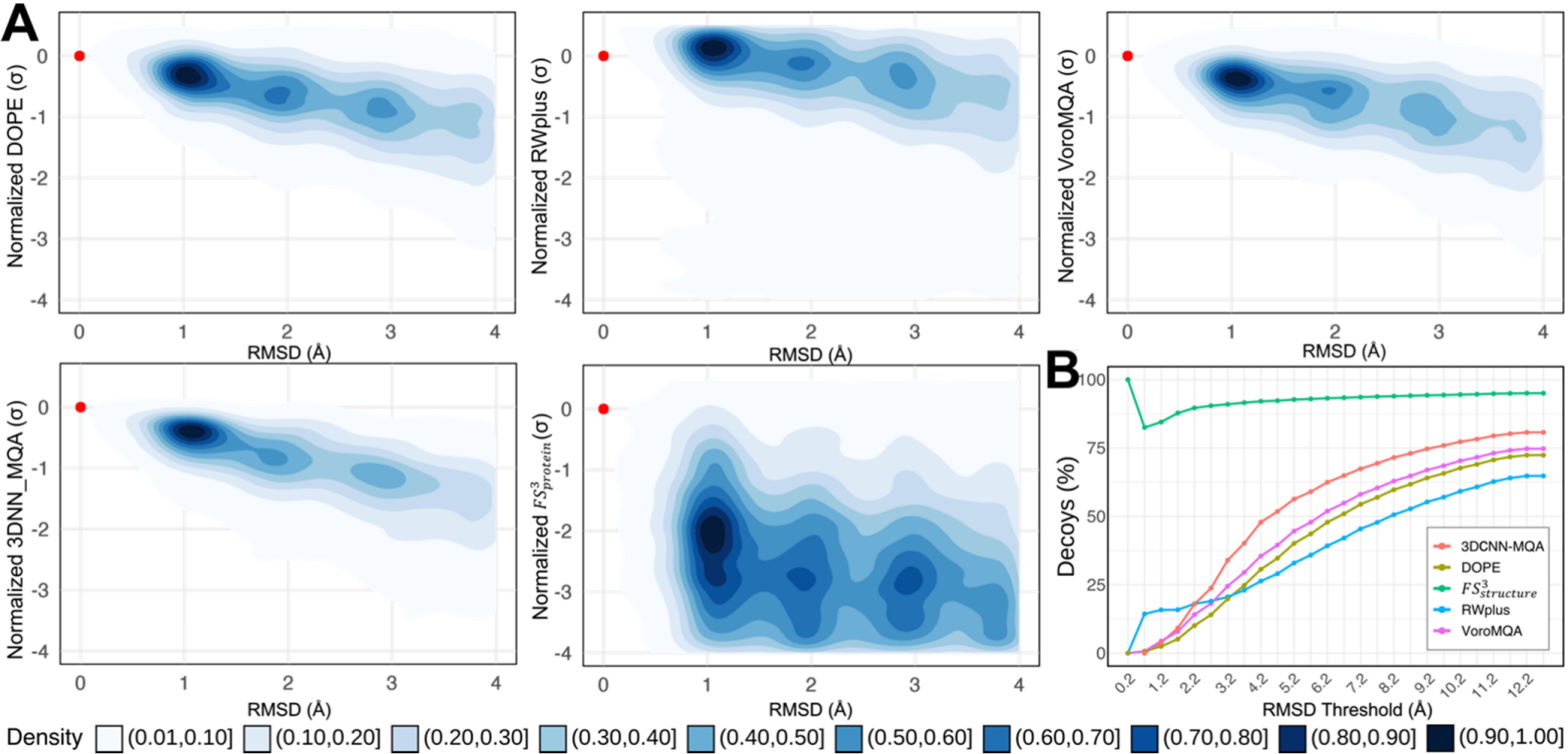
Combined densities of scores obtained when benchmarking the *FS*_protein_ scoring function against established scoring functions on the 3DRobot data set. *Y*-axis: normalized scores, where unity is the standard deviation of the scores within each native-decoy set, the score of each native X-ray structure set to 0 (red dot), and scaling is done so that more positive values indicate better scores. *X*-axis, RMSD of each decoy to native. (B) Graph showing (*y*-axis) the percentage of decoys related to each native X-ray structure for which the score of this native X-ray structure differs by at least one standard deviation, as a function of RMSD threshold.

To generalize these otherwise qualitative results, we characterized a test set of 359 high resolution structures (resolution better than 1.0 Å). As a result, the *FS*_atom_^3^ scores appear informative about structural indicators such as B-factor (Figure 5A) and are clearly affected by solvent accessibility (Figure 5B). This is true for *FS*_atom_^3^ scores calculated both with and without crystallographic water molecules (100 866 waters in the set). For atoms located at the surface of the protein, usually more mobile or subject to crystal anisotropy (higher B-factor) and more solvent exposed (higher SASA), scores often significantly improve upon consideration of water molecules (Figures 3B,3C and 5A). This means the individual *FS*_atom_^3^ scores for solvent-exposed atoms need to be corrected by including solvation, which has implications when considering ligand interactions in knowledge-based scoring functions, as accounted by others.^15–17,30^ That being said, when it comes to a global *FS*_structure_^3^ score, solvent-exposed atoms often represent a small proportion of the total number of atoms (Figure 5B) and thus have minimal impact.

**Figure 5:**
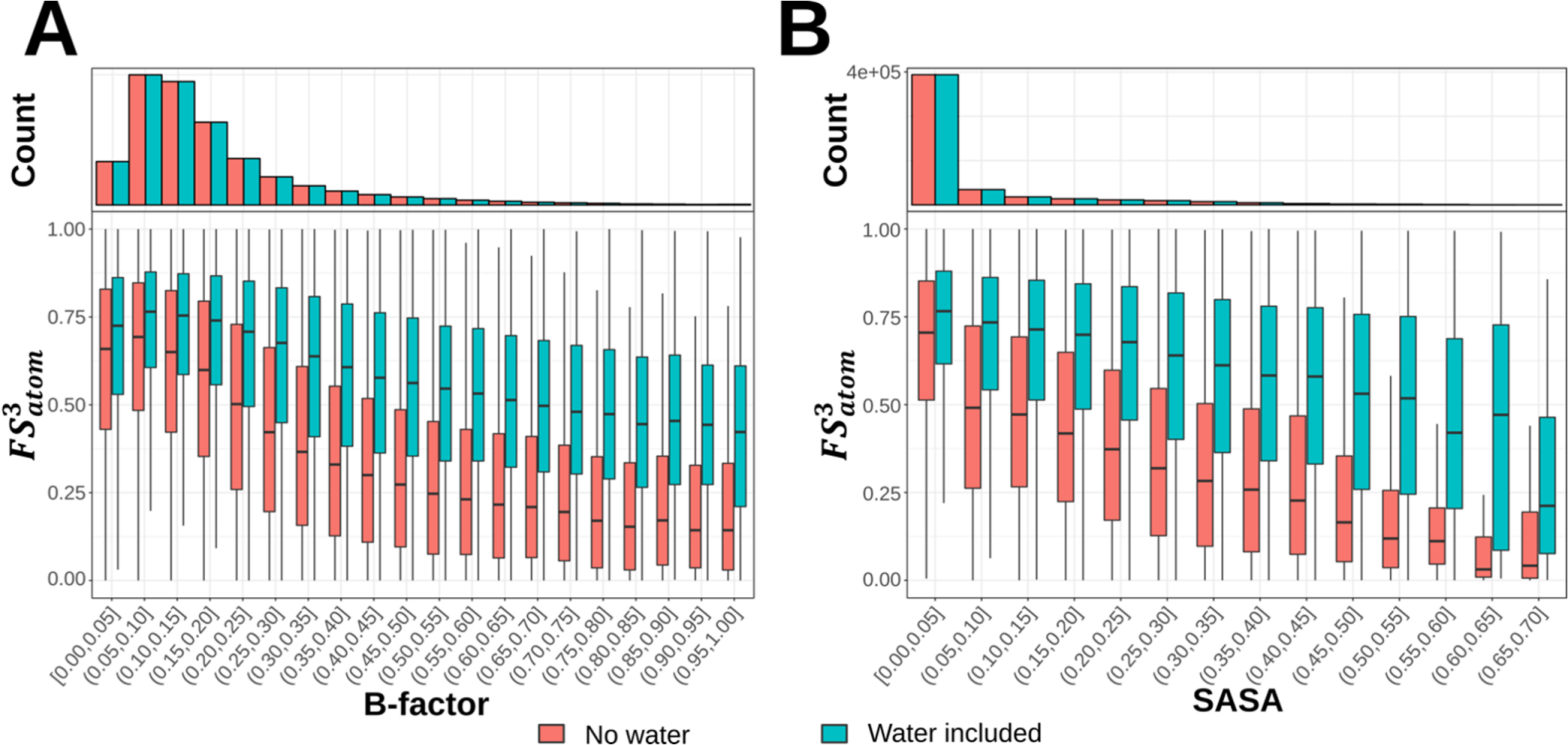
Distribution of the *FS*_atom_^3^ scores for all atoms from 359 high resolution structures (resolution <1.0 Å) pooled together. The scores are calculated either considering crystallographic water molecules (blue) or without (red). Top, counts of atoms. Bottom, boxplot of the scores as a function of (A) *B*-factor or (B) solvent accessibility. Each box represents 50% of the data.

### Contact Preference *CP* Scores for Atom Pairs, Accounting for Ranks in the Neighbor Lists

An alternative way to assess molecular contacts is to consider pairs of interacting atoms (origin, neighbor), akin to the R_F_ score introduced by Taylor.^13^ We developed an approach where, for each atom pair, we calculated a *CP* score, i.e., the ratio of the frequencies of observing and expecting this pair, taking the ranks in the neighbor list into account (eq 4) (Figure 6). The data can be summarized into sets of matrices, which fit well with our knowledge about molecular interactions. For example, the contact preference *CP* between ion pair atoms is high: it is 4.9 times more likely to observe a carboxylate oxygen counterion (Oox) at rank 1 of a basic nitrogen (Nbas), compared to the background frequencies of carboxylate oxygens at rank 1 (Figure 6B). Similarly, but less prominently, the contact preference *CP* for hydrogen bonding atoms is above unity. The most under-observed contacts are amide–amide (Nam–Nam) and carbonyl–carbonyl (Oc–Oc). This is probably due to these contacts being repulsive. When the ranks increase, the contact preference *CP* tends toward unity, i.e., an equal frequency of observing and expecting contacts.

**Figure 6:**
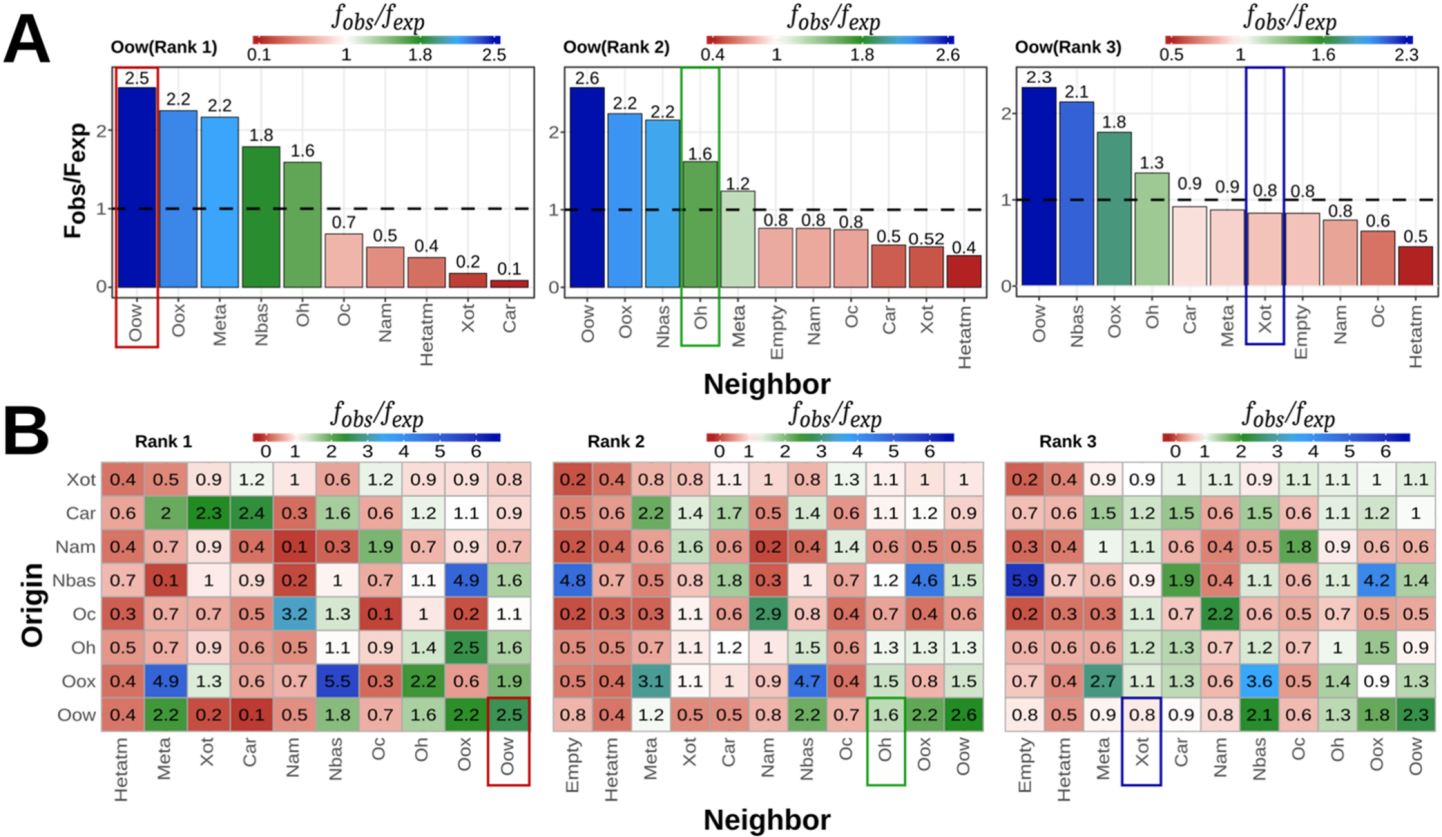
Contact preferences *CP* given as the ratio of the frequencies of observing an atom of a given type at a given rank (*f*_obs_) on the background frequencies at that rank (*f*_exp_). (A) *CP* around the three first ranks for an Oow origin. Boxes, types observed for selected examples. (B) Matrix of *CP* for all origin-neighbor combinations for the first three neighbors. The empty type may or may not be found at a given rank in a set of data. Color gradients are set within each matrix and are provided here only as a visual guide.

The contact preference *CP* can be illustrated on a practical example with the environment of water 925 (see also Figure 3A, discussed earlier). The contact preference *CP* (eq 4) at the ranks 1, 2, and 3 are respectively 2.5, 1.6, and 0.8 (see boxes on Figure 6A,6B). The contact preference *CP* is amenable to a visual output in 3D structures that is independent from the *FS*_atom_ score (Figure 7). Such output can be generated by parsing the contact lists, filtering contact preferences *CP* above or below a given threshold. Applied to the example (Figure 7), the three contacts with the highest preference are highly relevant: they correspond to a salt bridge with the C-terminus (Asn46) as well as to a sulfur-π stacking contact with the disulfide bridge involving Cys4. The current script calculates directional arrows indicating origin atoms, but reciprocal contact preferences could also be calculated.

**Figure 7:**
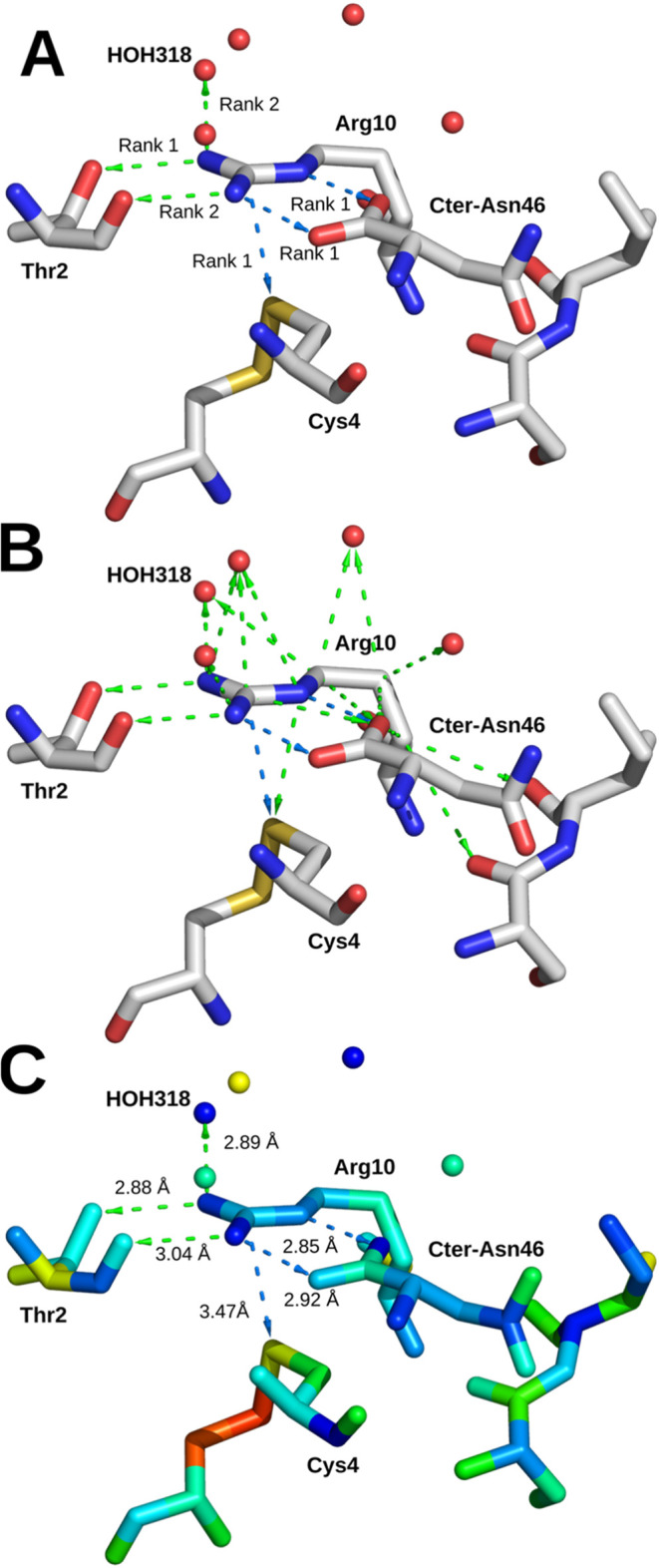
Contact preferences *CP* (directional dashed lines, CP above 1.5, blue; otherwise above 0.5, green) for atom pairs, exemplified for arginine 10 of a crambin crystal structure (PDB code 3NIR). (A) Six selected contacts, corresponding to classical hydrogen bonds, with their ranks in the contact list. (B) The first three contacts for every side-chain atom. (C) Plot combining the *FS*_atom_^3^ score with the contact preference *CP*, with distances mapped.

### Evaluating the Training Data—Molecular Environments Taken as Tuples

We also investigated whether elements of the molecular environment lists can be considered at once as a single tuple. These lists of tuples can be constructed preserving the rank information or without order (the data associated with the tuple are pooled for all the combinations of types included in the tuple at different ranks). There are thus more tuples in the lists preserving the rank information than in the unordered ones; the maximum theoretical numbers of tuples using three positions and 11 atom types in those lists are respectively 1331 and 286 (Figure 8). Environments preserving ranks contain more information, however they do not provide enough data points for representative statistics for scarcely populated environments. On the other hand, unordered-list environments cannot condition atom types to ranks in the neighbor list. Thus, each method carries its own advantages and drawbacks.

**Figure 8:**
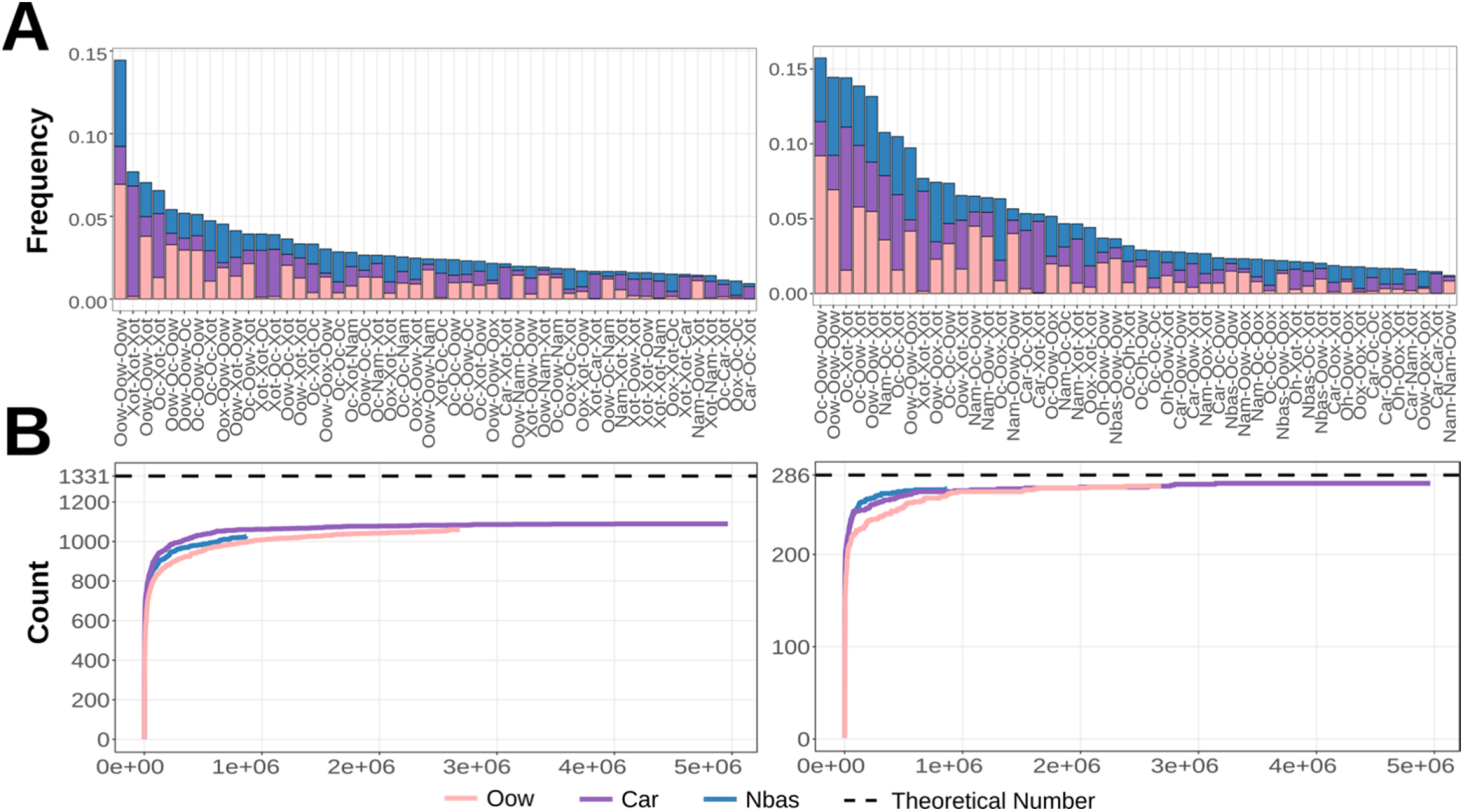
Count for environments (size 3) in the training set for the origins Oow, Car and Nbas (2,683,524, 4,965,111, and 867,579 origins considered respectively). Environments are represented by triplets of atom types that can preserve rank information (left-hand panel) or be unordered (right-hand panel). (A) Histogram combining the 50 most frequent environments for three origins (Oow, Nbas, and Car). (B) Cumulative counts of possible environments and maximum theoretical number (dashed line).

Interestingly, some tuples are more often observed than others (Figure 8A). For example, negatively charged counterions (Oox) are often seen near basic origins (Nbas), and purely hydrophobic environments (tuples composed of exclusively Xot and Car) are seldom seen for charged queries. In fact, for Car, Nbas, and Oow, respectively 242 (18.2%), 305 (22.9%), and 271 (20.4%) out of 1331 possible rank-preserving environments are never observed. Similarly, 9 (3.2%), 17 (5.9%), and 14 (4.9%) out of 286 possible unordered-list environments are never observed. We attempted to take advantage of this and develop a score based on the ratio of observed to expected frequencies of unordered-list tuples of different sizes. The performance appeared low, and this scoring function was not further investigated.

Another interesting consideration is the number of data points required to observe different environments. When queries are used to scan the training data set, for each query, the number of observed unique environments reaches a plateau (Figure 8B). Considering the tuples constructed using ordered-list and unordered-list environments, the observation is similar irrespective of the query: about a million queries are necessary to see 99% of all possible rank-preserving tuples. Conversely, with only half a million queries (10%) we train ∼85–90% of all environments. This provides orders of magnitude relevant to training future scoring functions.

## Discussion

### Application of the *FS*_atom_ and *CP* Scores for Structural Analysis

This paper analyzes atomic environments as rank-ordered lists. We demonstrate that, when conditioned by ranks, the environment distributions are very regular (Figure 2 and Supporting Information 2 and 3). This feature was used to develop a function scoring the fitness of an atom with respect to its neighbors, the *FS*_atom_^*k*^ score, which can be generalized to score full atomic structures as a *FS*_structure_^k^ score. When benchmarked against the 3DRobot set (Figure 4 and Supporting Information 4), the performance of the *FS*_structure_^k^ score was excellent. We also established the preferences of pairs of interacting atoms, i.e., a contact preference *CP* (Figure 6). These two assessments can be combined for a powerful and easily computed visual evaluation of molecular complexes (Figure 7C). The visual output is easy to compute and may perform better than simple tools to visualize molecular complexes based on simple distances in its ability to capture the most relevant molecular interactions, for example those integrated in PyMOL,^26^ Mol viewer,^35^ NGL viewer,^36^ and PLIP.^37^ While working well in the general case (Figure 7), it should be noted that the ease of calculation of the *CP* scores is balanced by some approximations (in contrast to R_F_ scores): R_F_ scores are considering the surface of each atom accessible to interactions for normalization, while *CP* use a more coarse frequency of expecting contacts based on counts; and *CP* also do not account for van der Waals radii in the computations, and thus may miss primary contacts of large atoms such as a ligand iodine atom.

In the present work, *FS*_atom_^*k*^ is developed directly from PDB files that typically do not include hydrogen atoms. While it adds approximations, at the same time this allow to work directly with the raw PDB files. The computation of *FS*_atom_^*k*^ and *CP* is fast, and does not require complex processing of the data, nor prior knowledge such as SMART fragments. Interestingly, *FS*_atom_^3^ scores are lower for solvent-exposed atoms in unsolvated proteins (Figures 3B, 3E and 4), as was observed by others.^30^ There is thus a potential to correct the *FS*_atom_^3^ a score, either implicitly or explicitly by positioning water molecules directly at the protein surface.

### Number of Relevant Neighbors

We empirically set the number of neighbors used to *k* = 3, based on the existing data: the rank dependence (Supporting Information 2); the density distribution graphs (Figure 2 and Supporting Information 3); the effect of the number of neighbors on score vs *B*-factor (Figure 5). This number *k* = 3 could be, for each query, optimized. From a chemical and geometrical perspective, the number of close interactions around an atom depends on its van der Waals radius and relates inversely to the number of connected atoms. For example, a primary amine functional group has more space around than a tertiary amine group, and thus could theoretically accommodate more interacting atoms.

Changing the number of neighbors included in the computation of the *FS*_atom_^*k*^ scores to *k* = 1 (Supporting Information 6) lowers the trend of scores following *B*-factor and solvent accessibility, which may be caused by too few atoms included in the calculation. In contrast, when the number of neighbors is increased to *k* = 10, scores appear very similar to one another and therefore less discriminative, suggesting that an optimal number of neighbors exists for calculating scores, which may be atom-type dependent.

A way to investigate the contact atoms is through the projection of the environment counts using a simple correspondence analysis (Figure 9; see also Supporting Information 7). In this projection, both atom types and ranks are represented as points in the same space, where their relative proximity reflects the strength of their statistical association. Atom type points located near a rank point in the plot correspond to atom types that are most frequently observed at that rank in neighbor lists. All the starting (rank 1) environments are very specific. For basic nitrogen (Nbas) queries, the first neighbor is most frequently a carboxylate oxygen (Oox) (Figure 9A). For water molecules (Oow), rank 1 environments are dominated by polar atoms (Oc, Oh, Oox) and metals (Figure 9B). For aromatic carbons (Car) the closest environments are predominantly aromatic (Car) or charged (Nbas) (Figure 9C). When following the progression of ranks, the simple correspondence analysis shows inflections at 3 or 4 neighbors. As discussed previously, the environment at higher ranks seems to be converging toward the baseline environment seen by any atom, markedly toward the Empty atom type.

**Figure 9:**
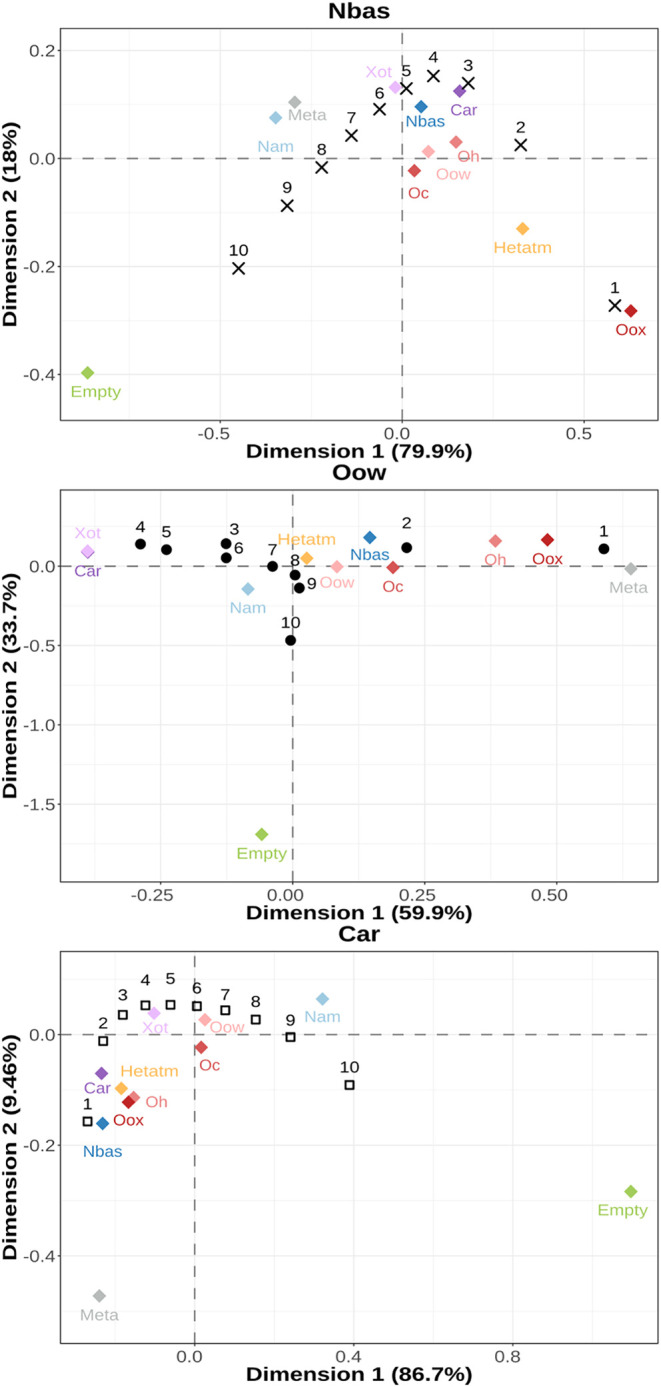
Projection using a simple correspondence analysis of the custom atom types and the first 10 neighbors for the Nbas (Top, cross), Oow (Middle, plain circle) and Car (Down, squares) origins. The first ten neighbors are projected on dimensions 1 and 2, which explains most of the data.

### Reference State

A key question in scoring function is the setting of reference state. Several approaches have been used in the literature.^10,11,30–33^ The scoring function used in this manuscript, the *FS*_atom_^*k*^ score, is based on pair potentials and does not directly use a reference state. While it can be argued that this is a drawback toward a mechanistically sound scoring function, we believe that stratification by rank significantly attenuates the need for accounting for the reference state. With similarities to the *FS*_atom_^*k*^ score, some machine learning scoring functions such as the machine-learning function RF-score use binned pairwise atomic distances as descriptors, without a reference state.^38^

## Conclusion

In this manuscript, we show that contact lists can be stratified by ranks, and that this property can be used to develop simple methods of scoring and assessing contact preferences. The fitness score *FS*_atom_^*k*^ presented here allowed a very strong discrimination of decoys and native on the 3DRobot benchmark set. The methods proposed here are amenable to large-scale unsupervised machine learning applications, since they use lists and no complex geometrical calculations beyond the initial distance calculations that is well handled by the KDtree algorithm. They could easily be used by standard structure file viewers for improved visualization of molecular interaction. The functions are simple enough to be translatable to any atoms, for example ligands or metals, without any specific adaptation.

## Supplementary Material



## Data Availability

The script that allows calculation of the fitness score and the preference score from a pdb structure is available at https://github.com/DreanoLoic/Fitness_score.git. The output is an annotated PDB file, a PyMOL session, and a csv table. The script can handle the conversion to Sybyl type to work with ligands, which will be described elsewhere.

## Notes

S1, Custom element types and their attributes; S2, Neighbor composition at different ranks. S3, distance densities of neighbors stratified by their ranks; S4, Benchmarking against established scoring functions; S5, Contact preferences matrices; S6, Influence of the number of neighbors on the score distributions, and S7, Correspondence analyses (PDF)

## §

also known as Evgeni Grazhdankin.

## Abbreviations

FS: fitness score
FS_atom_^*k*^: atomic fitness score
FS_structure_^*k*^: protein fitness score
CP: contact preference
Car: Aromatic carbon
Nbas: basic nitrogen
Oow: Water oxygen
Oox: carboxylate oxygen
Oc: carbonyl oxygen
Oh: hydroxyl oxygen
Nam: amide nitrogen
Xot: aliphatic and sulfur atoms
PDB: Protein Data Bank
RMSD: root-mean-square deviation
SASA: solvent-accessible surface area
