# Supplementary material for: Assessing Molecular Contacts Using Atom Environments Described by Ranked Lists
Source: J Chem Inf Model. 2026 Jul 16;66(14):8228–38. doi: 10.1021/acs.jcim.6c00694 (PMC13417873; doi:10.1021/acs.jcim.6c00694)
Supplement: Supplementary file 1 [file ci6c00694_si_001.pdf]

Supporting information for

Assessing Molecular Contacts Using Atom Environments described by Ranked Lists

Loic Dreano [†]; Ashenafi Legehar [†]; Mael Briand [†]; Kuura Variskallio [†]; Alexandre Borrel [‡]; Henri Xhaard [*†]

ORCIDs: L.D. 0000-0001-8280-3904; A.L. 0000-0001-9706-4139; A.B. 0000-0001-6499-4540; M.B. 0000-0001-6801-7587; K.V. 0000-0001-7932-5497; H.X. 0000-0002-3000-7858

† Drug Research Program, Division of Pharmaceutical Chemistry and Technology, Faculty of Pharmacy, Helsinki FI-00014, University of Helsinki, Finland.

‡ Sciome LLC, Research Triangle Park, North Carolina 27713, United States.

* Email: henri.xhaard@helsinki.fi

Table of contents :

Supporting information 1. Custom element types and their attributes. 2

Supporting information 2. Composition for the ranks 1 to 10 for all 10 origins 3

Supporting information 3. For each origin atom, distance densities of neighbors stratified by their rank in the environment. 6

Densities that are well stratified: 6

Densities that are less well stratified: Meta, Hetatm, Nam and Xot. 19

Supporting information 4. Benchmarking the $FSprotein3$ against established scoring functions using the 3DRobot dataset.. 27

Supporting information 5. Contact preferences matrices for the 8 protein atom type origins at the 10 ranks.. 29

Supporting information 6. Influence of the number of neighbors k on the $FSatomk$ score distribution. 32

Supporting information 7. Correspondence analyses for the 10 origins. 33

Supporting information 1. Custom element types and their attributes.

Custom types	Included atoms	Description
**H**	H	All hydrogen atoms.
**Car**	C in aromatic rings, ARG (CZ), GLN (CD), GLU (CD), ASP (CG), ASN (CG)	Aromatic rings and selected side chain carbon atoms.
**Nbas**	N in ARG (NH1, NH2, NE), HIS (NE2, ND1), LYS (NZ)	Nitrogen atoms with basic properties.
**Nam**	N in amide groups of ASN (ND2), GLN (NE2), TRP (NE1), peptide main chain	Nitrogen atoms in the amide groups of ASN and GLN, peptide main chain, and the indole nitrogen of TRP.
**Oh**	O in hydroxyl groups of SER (OG), THR (OG1), TYR (OH)	Oxygen atoms in hydroxyl groups.
**Oc**	O in carbonyl groups of ASN (OD1), GLN (OE1), peptide main chain	Oxygen atoms in carbonyl groups.
**Oox**	O in carboxylate groups of ASP (OD1, OD2), GLU (OE1, OE2), C-terminus (OXT)	Oxygen atoms in carboxylate groups or at the C-terminus.
**Xot**	Aliphatic carbons (in all amino acids except GLY), sulfur of CYS (SD) and MET (SG), rare atoms not fitting predefined categories.	Aliphatic carbons and sulfur. Generic category for rare atoms.
**Oow**	Water molecules HOH (O)	Oxygen atoms in water molecules.
**Meta**	Metal atoms (e.g., FE, MG, CA)	Metal ions.
**Hetatm**	Atoms from ligands or other heteroatoms	Atoms from non-standard residues or ligands, excluding water molecules.
**Empty**	None	Placeholder for an environment that has fewer neighbors than the specified size.

Supporting information 2. Composition for the ranks 1 to 10 for all 10 origins. Pooled on 47,770,954 origin atoms (Car : 4,965,111; Hetatm : 1,405,936; Meta : 13,600; Nam: 6,314,449; Nbas : 867,579; Oc :5,988,566; Oh :838,602; Oow : 2,683,524; Oox : 1,377,987; Xot : 23,315,400).The neighbor lists are shown without (left-hand panels) or with (right-hand panels) the *primary contact* filter applied.

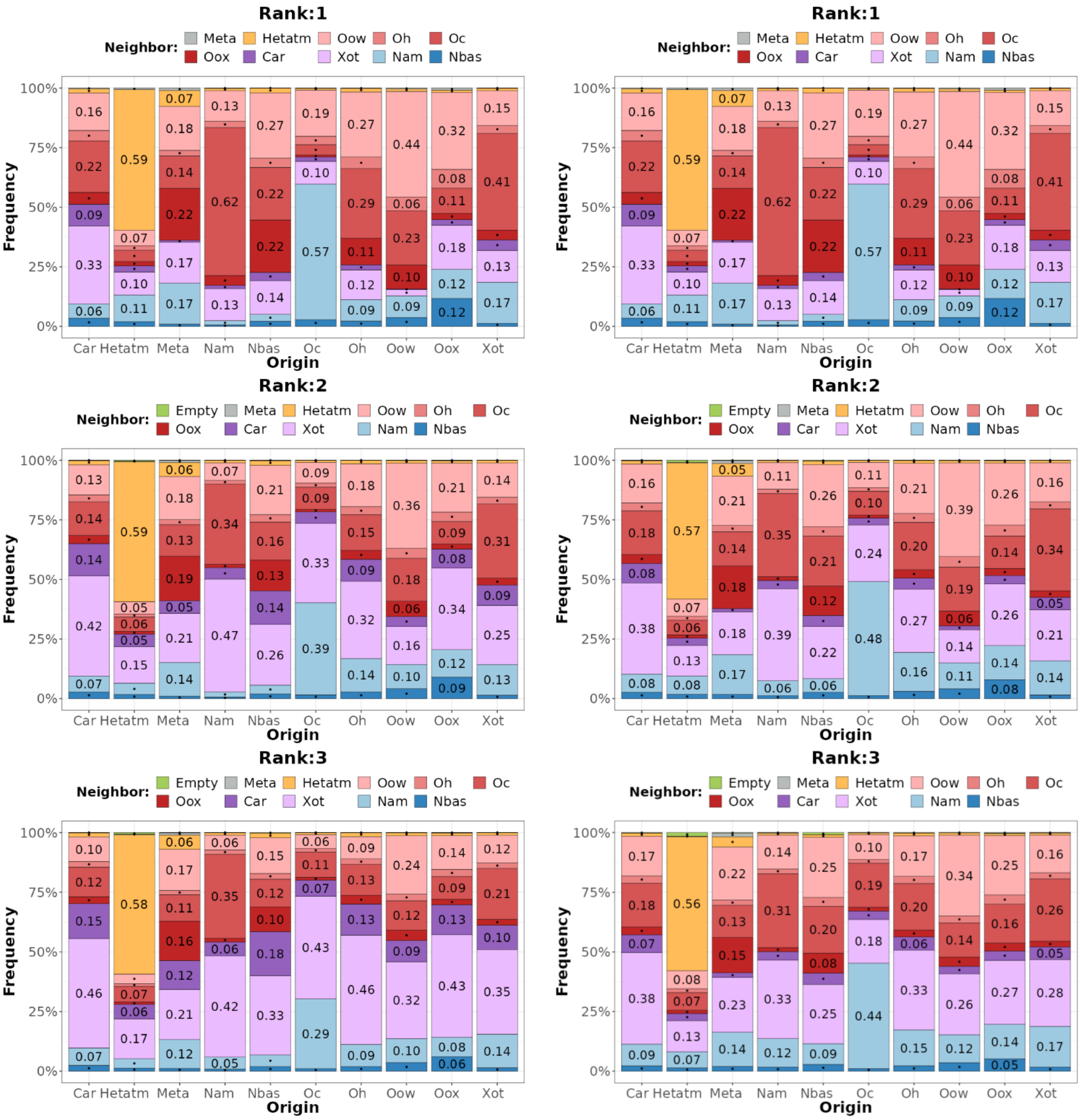

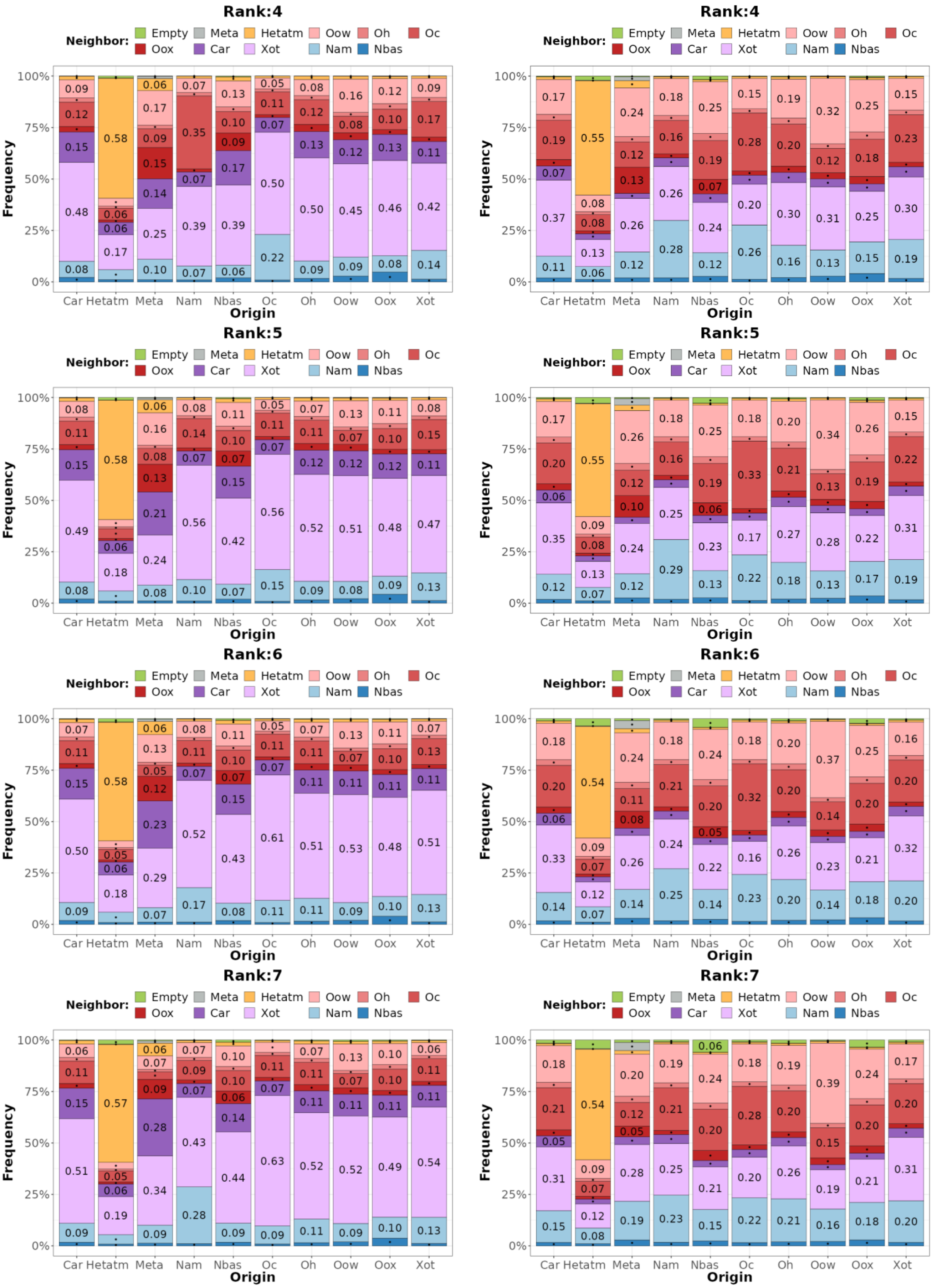
Rank:4
Neighbor: Empty Meta Hetatm Oow Oh Oc Oox Car Xot Nam Nbas
Frequency
100%
75%
50%
25%
0%
Car Hetatm Meta Nam Nbas Oc Oh Oow Oox Xot
Origin
Rank:5
Rank:6
Rank:7

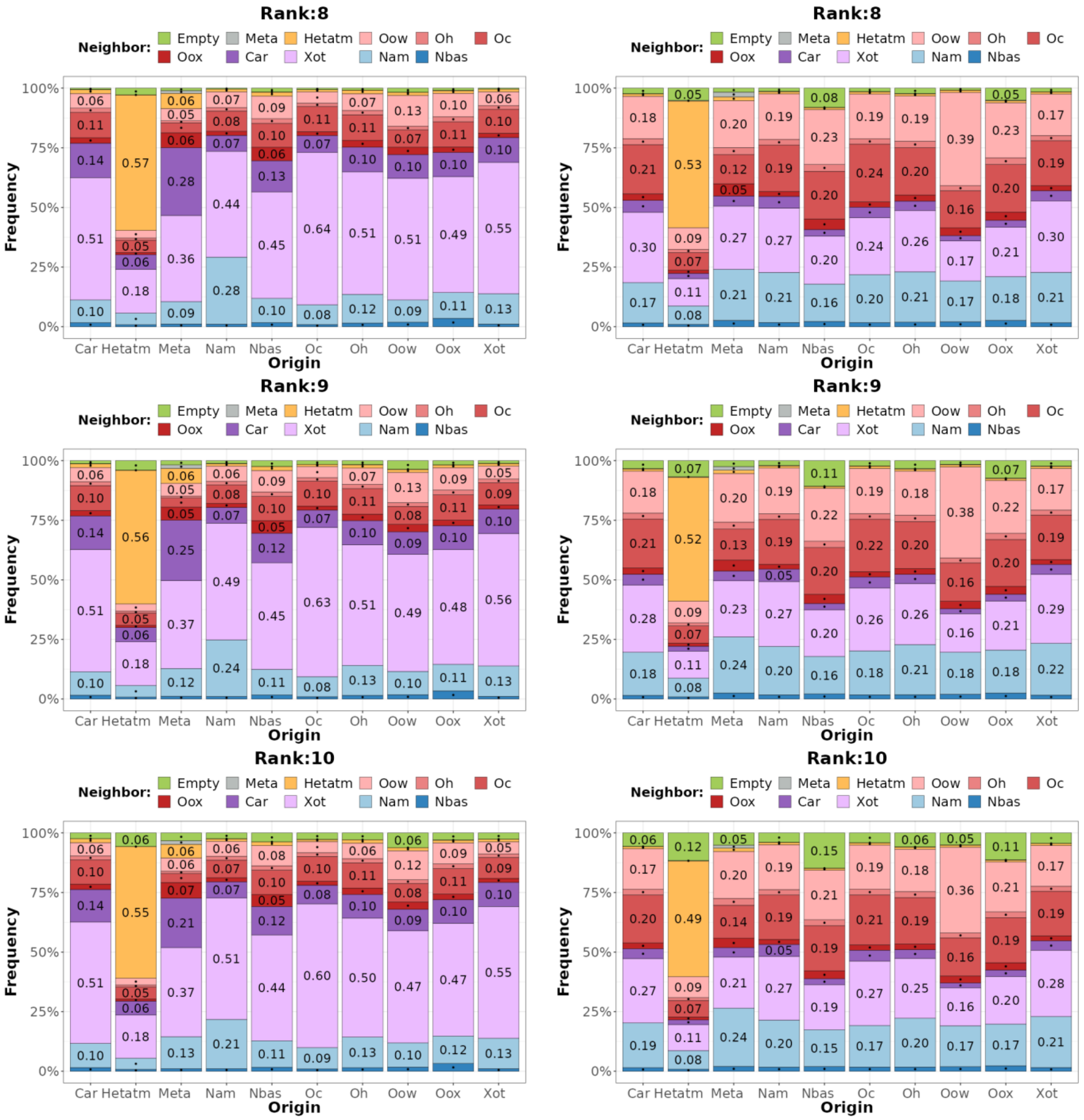
Rank:8
Neighbor: Empty Meta Hetatm Oow Oh Oc Oox Car Xot Nam Nbas
Frequency
100%
75%
50%
25%
0%
Car Hetatm Meta Nam Nbas Oc Oh Oow Oox Xot
Origin
Rank:9
Rank:10

Supporting information 3. For each origin atom, distance densities of neighbors stratified by their rank in the environment. Densities that are well stratified: Car, Nbas, Oc, Oh, Oow and Oox. The neighbor list is collected without (left-hand panels) or with (right-hand panels) the *primary contact* filter activated. The origin is given first in the label, e.g., Car(Nam) plots the distribution of the Nam neighbors from a Car origin at all 10 ranks.

Densities that are well stratified:

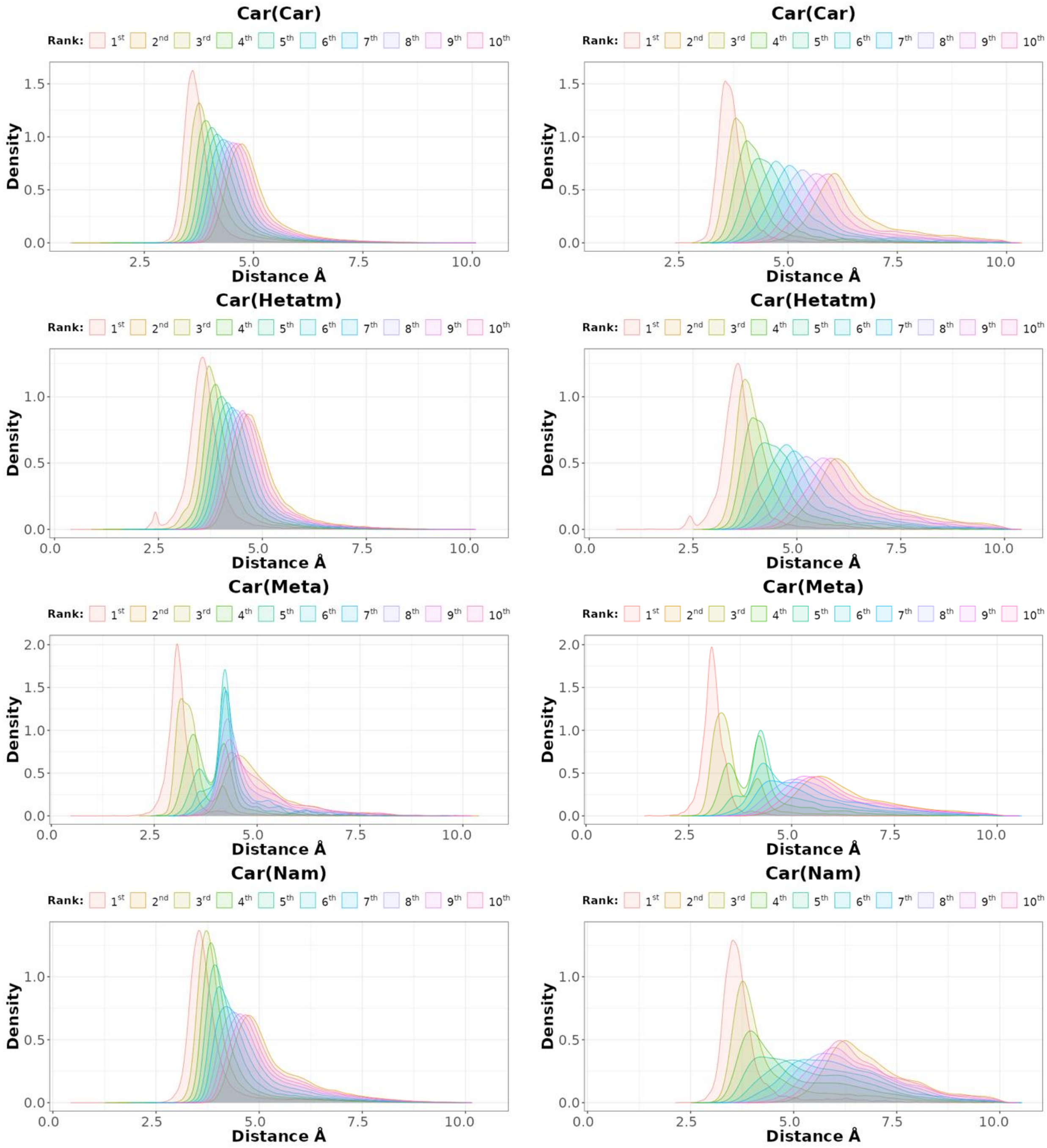

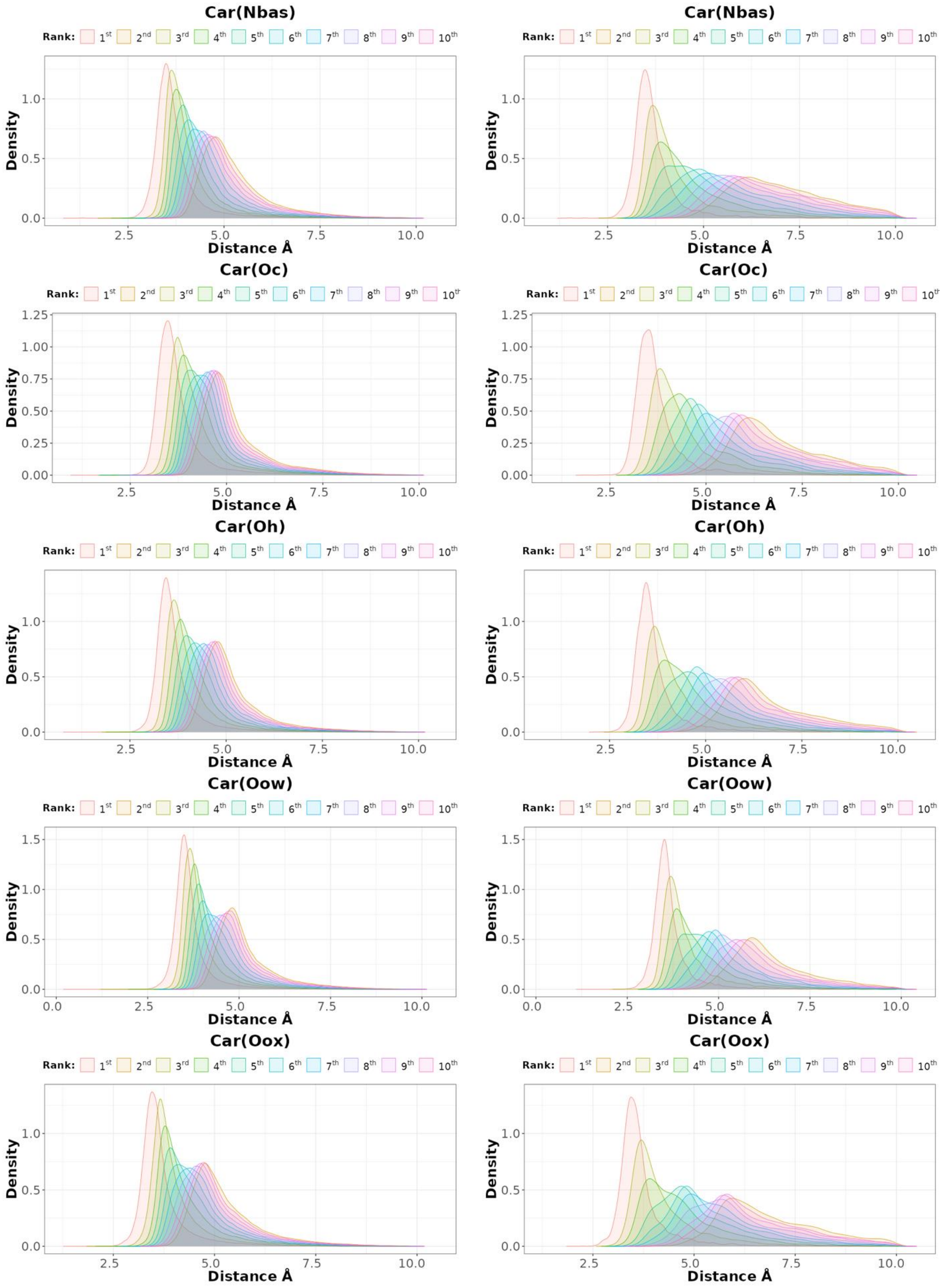
Car(Nbas)
Car(Nbas)
Car(Oc)
Car(Oc)
Car(Oh)
Car(Oh)
Car(Oow)
Car(Oow)
Car(Oox)
Car(Oox)
Rank: 1st 2nd 3rd 4th 5th 6th 7th 8th 9th 10th
Density
Distance Å

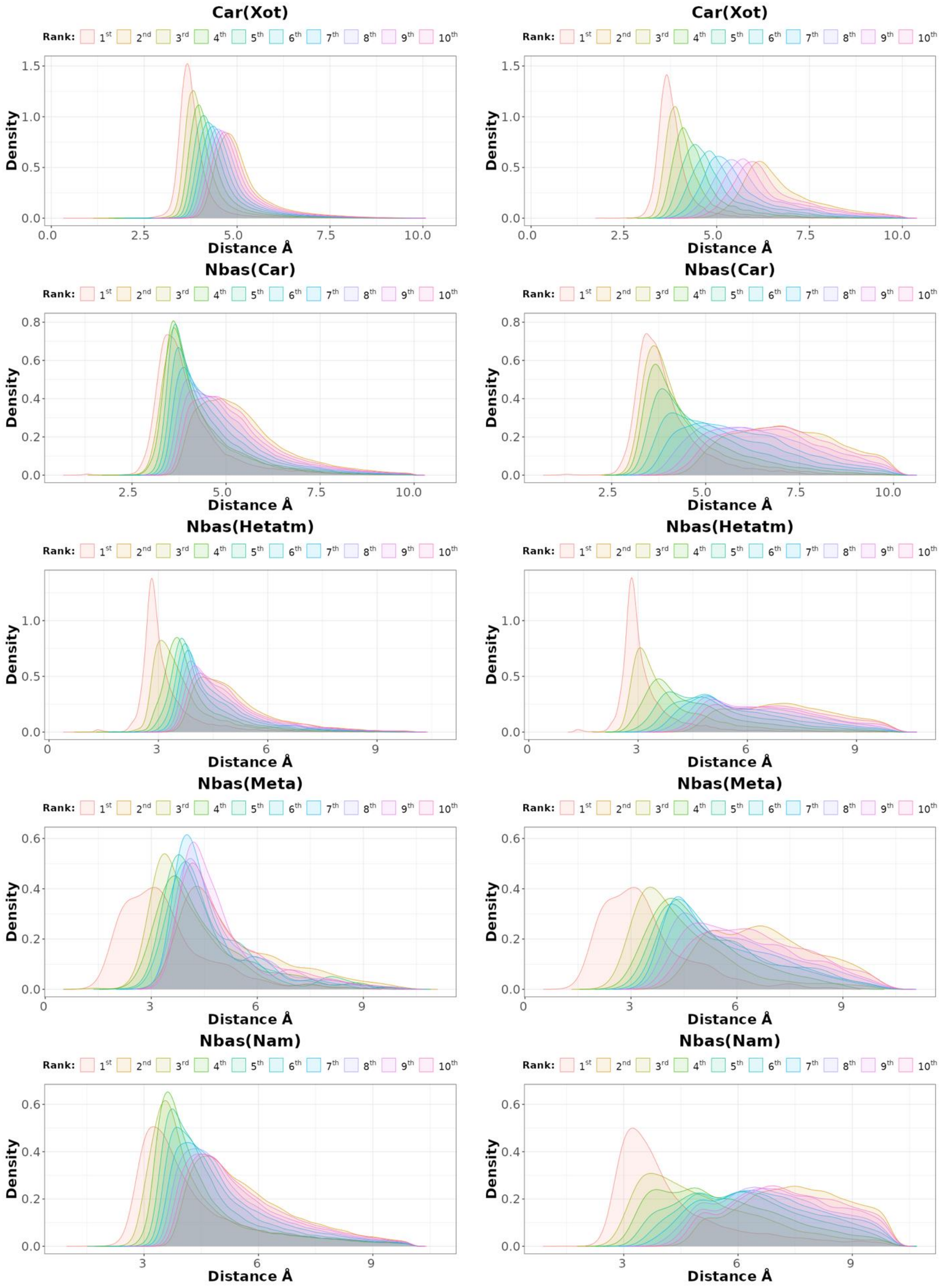
Car(Xot)
Rank: 1st 2nd 3rd 4th 5th 6th 7th 8th 9th 10th
Density
Distance Å
Car(Xot)
Rank: 1st 2nd 3rd 4th 5th 6th 7th 8th 9th 10th
Density
Distance Å
Nbas(Car)
Rank: 1st 2nd 3rd 4th 5th 6th 7th 8th 9th 10th
Density
Distance Å
Nbas(Car)
Rank: 1st 2nd 3rd 4th 5th 6th 7th 8th 9th 10th
Density
Distance Å
Nbas(Hetatm)
Rank: 1st 2nd 3rd 4th 5th 6th 7th 8th 9th 10th
Density
Distance Å
Nbas(Hetatm)
Rank: 1st 2nd 3rd 4th 5th 6th 7th 8th 9th 10th
Density
Distance Å
Nbas(Meta)
Rank: 1st 2nd 3rd 4th 5th 6th 7th 8th 9th 10th
Density
Distance Å
Nbas(Meta)
Rank: 1st 2nd 3rd 4th 5th 6th 7th 8th 9th 10th
Density
Distance Å
Nbas(Nam)
Rank: 1st 2nd 3rd 4th 5th 6th 7th 8th 9th 10th
Density
Distance Å
Nbas(Nam)
Rank: 1st 2nd 3rd 4th 5th 6th 7th 8th 9th 10th
Density
Distance Å

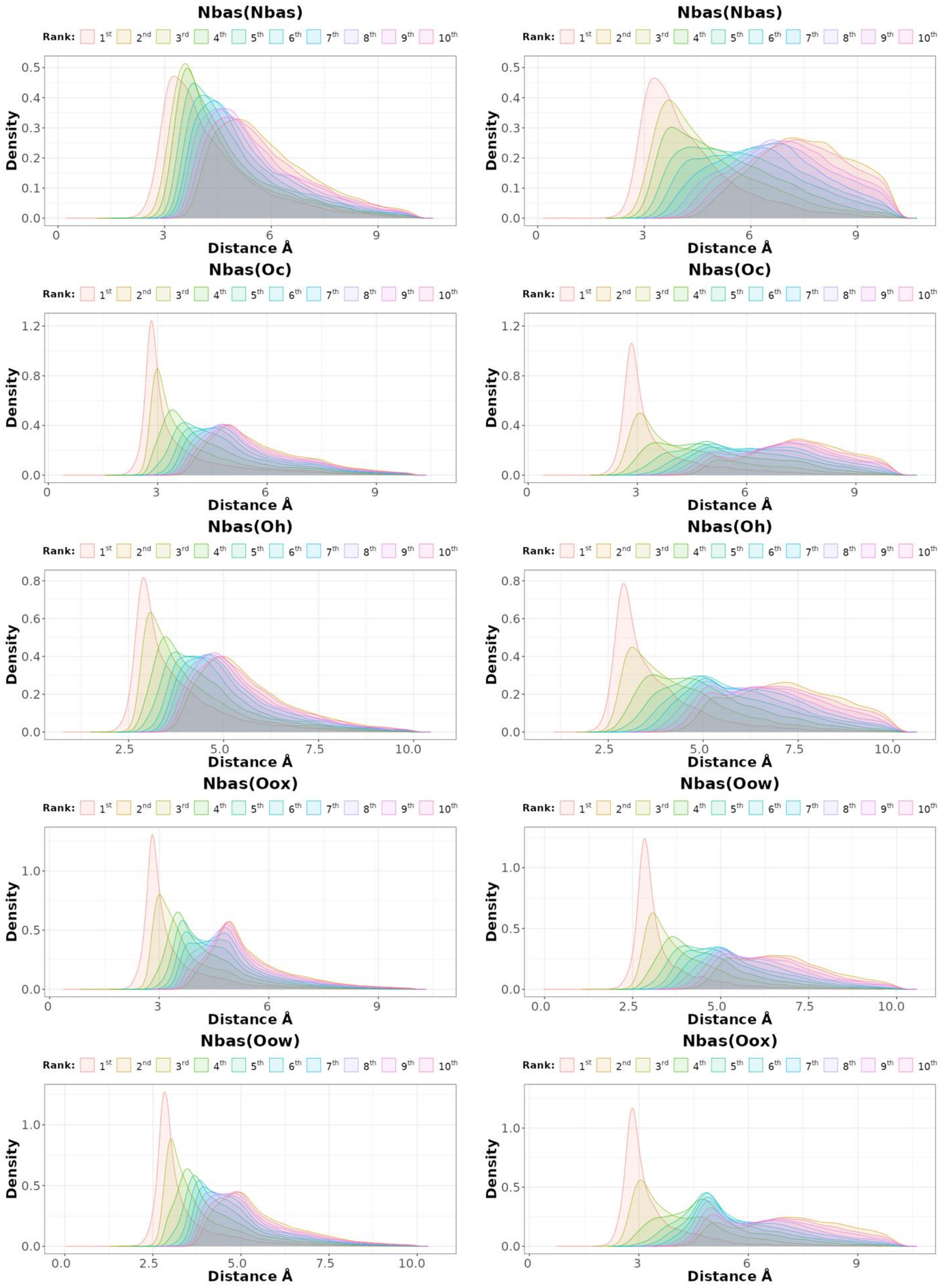
Nbas(Nbas)
Nbas(Nbas)
Nbas(Oc)
Nbas(Oc)
Nbas(Oh)
Nbas(Oh)
Nbas(Oox)
Nbas(Oow)
Nbas(Oow)
Nbas(Oox)
Rank: 1st 2nd 3rd 4th 5th 6th 7th 8th 9th 10th
Density
Distance Å

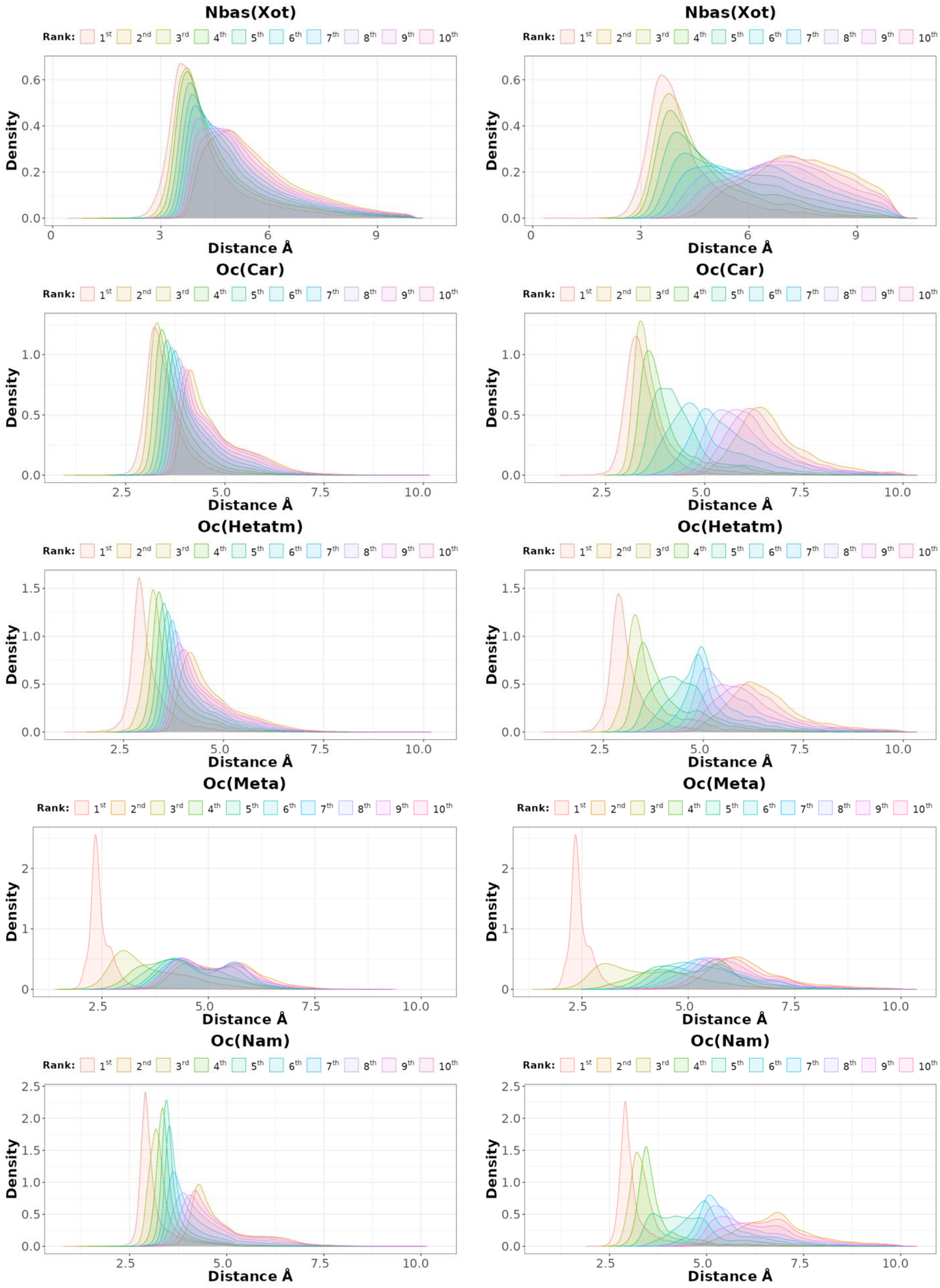
Nbas(Xot)
Nbas(Xot)
Oc(Car)
Oc(Car)
Oc(Hetatm)
Oc(Hetatm)
Oc(Meta)
Oc(Meta)
Oc(Nam)
Oc(Nam)
Rank: 1st 2nd 3rd 4th 5th 6th 7th 8th 9th 10th
Density
Distance Å

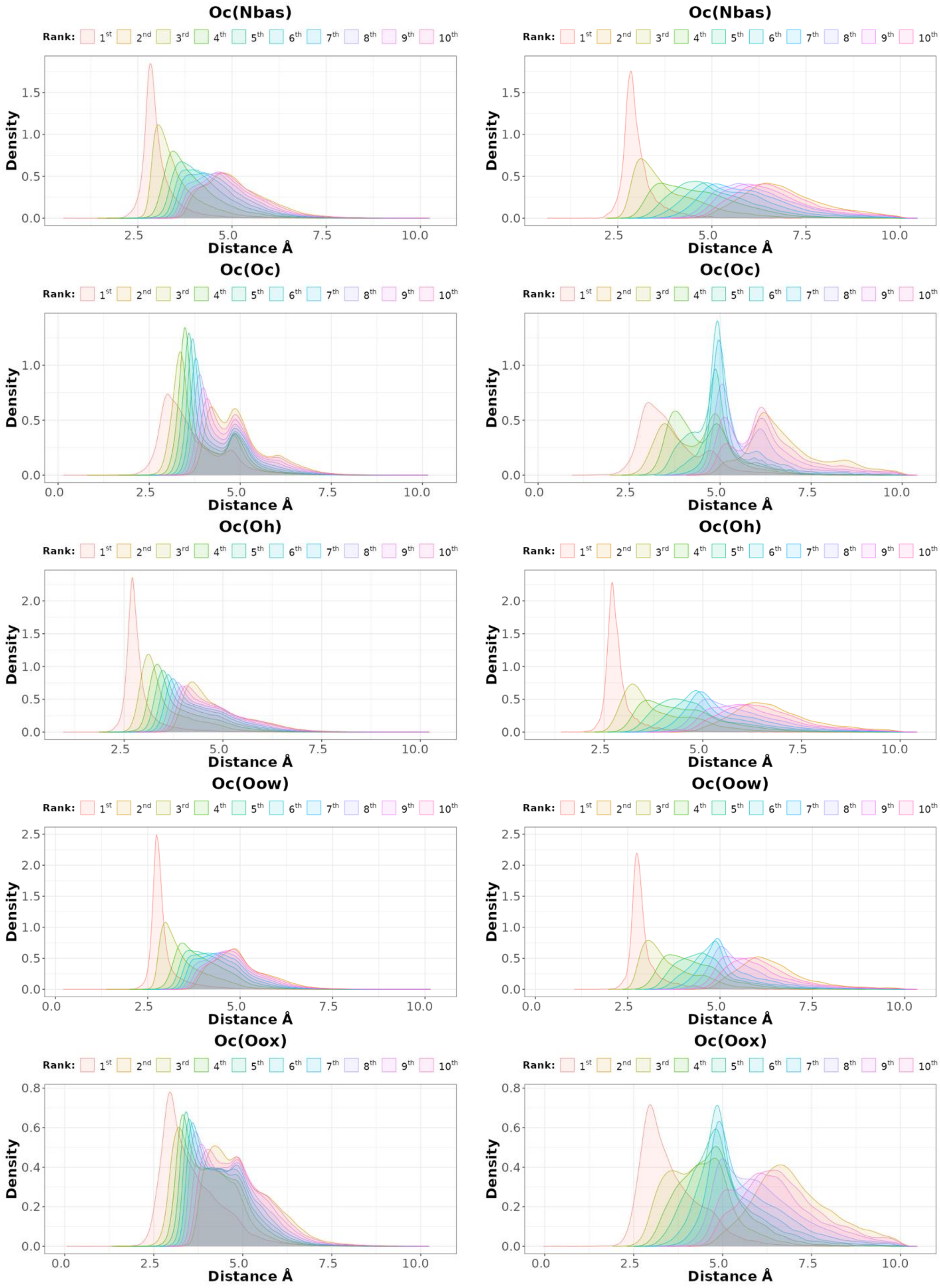
Oc(Nbas)
Oc(Oc)
Oc(Oh)
Oc(Oow)
Oc(Oox)
Rank: 1st 2nd 3rd 4th 5th 6th 7th 8th 9th 10th
Density
Distance Å

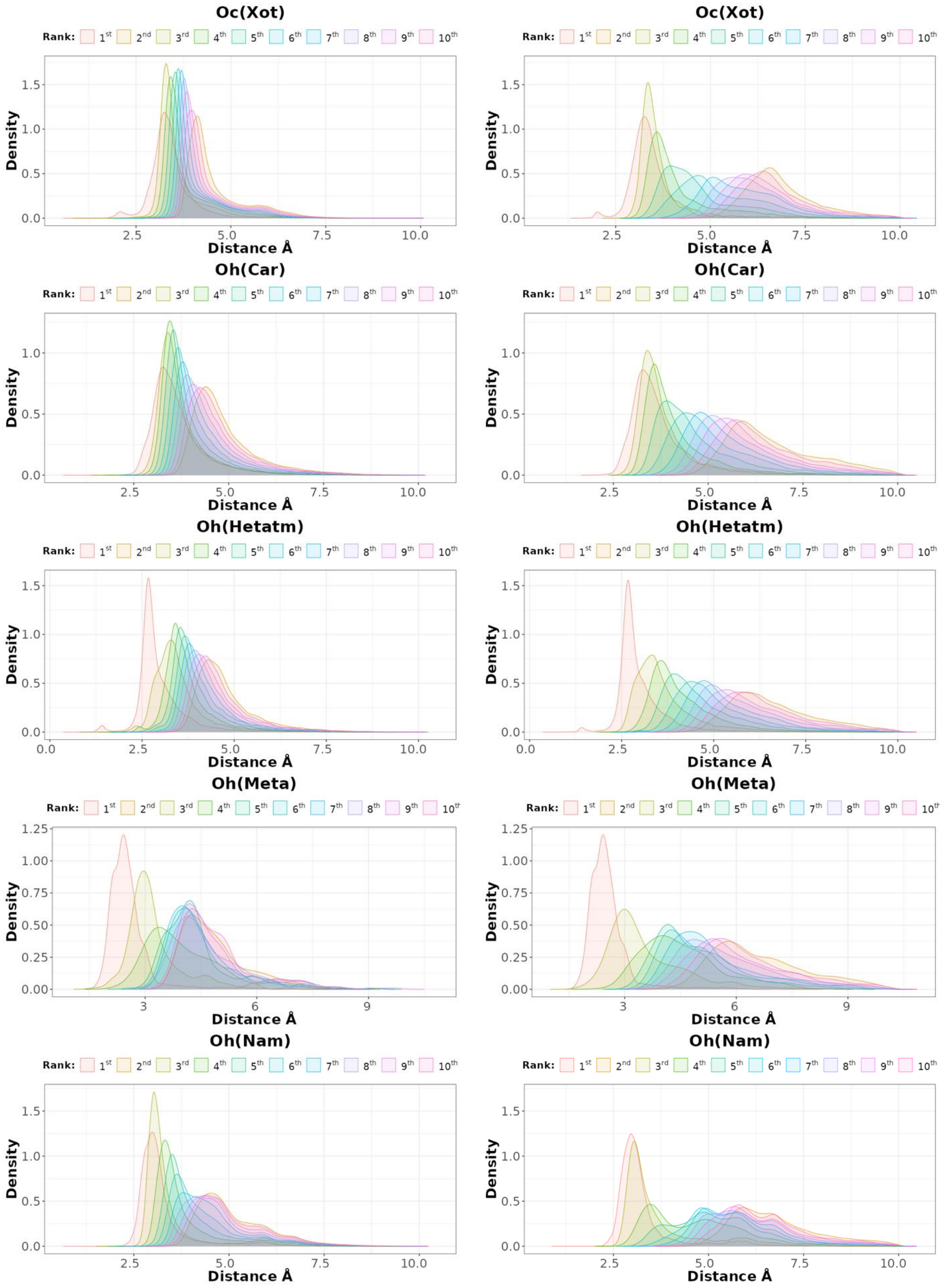
Oc(Xot)
Rank: 1st 2nd 3rd 4th 5th 6th 7th 8th 9th 10th
Density
Distance Å
Oc(Xot)
Rank: 1st 2nd 3rd 4th 5th 6th 7th 8th 9th 10th
Density
Distance Å
Oh(Car)
Rank: 1st 2nd 3rd 4th 5th 6th 7th 8th 9th 10th
Density
Distance Å
Oh(Car)
Rank: 1st 2nd 3rd 4th 5th 6th 7th 8th 9th 10th
Density
Distance Å
Oh(Hetatm)
Rank: 1st 2nd 3rd 4th 5th 6th 7th 8th 9th 10th
Density
Distance Å
Oh(Hetatm)
Rank: 1st 2nd 3rd 4th 5th 6th 7th 8th 9th 10th
Density
Distance Å
Oh(Meta)
Rank: 1st 2nd 3rd 4th 5th 6th 7th 8th 9th 10th
Density
Distance Å
Oh(Meta)
Rank: 1st 2nd 3rd 4th 5th 6th 7th 8th 9th 10th
Density
Distance Å
Oh(Nam)
Rank: 1st 2nd 3rd 4th 5th 6th 7th 8th 9th 10th
Density
Distance Å
Oh(Nam)
Rank: 1st 2nd 3rd 4th 5th 6th 7th 8th 9th 10th
Density
Distance Å

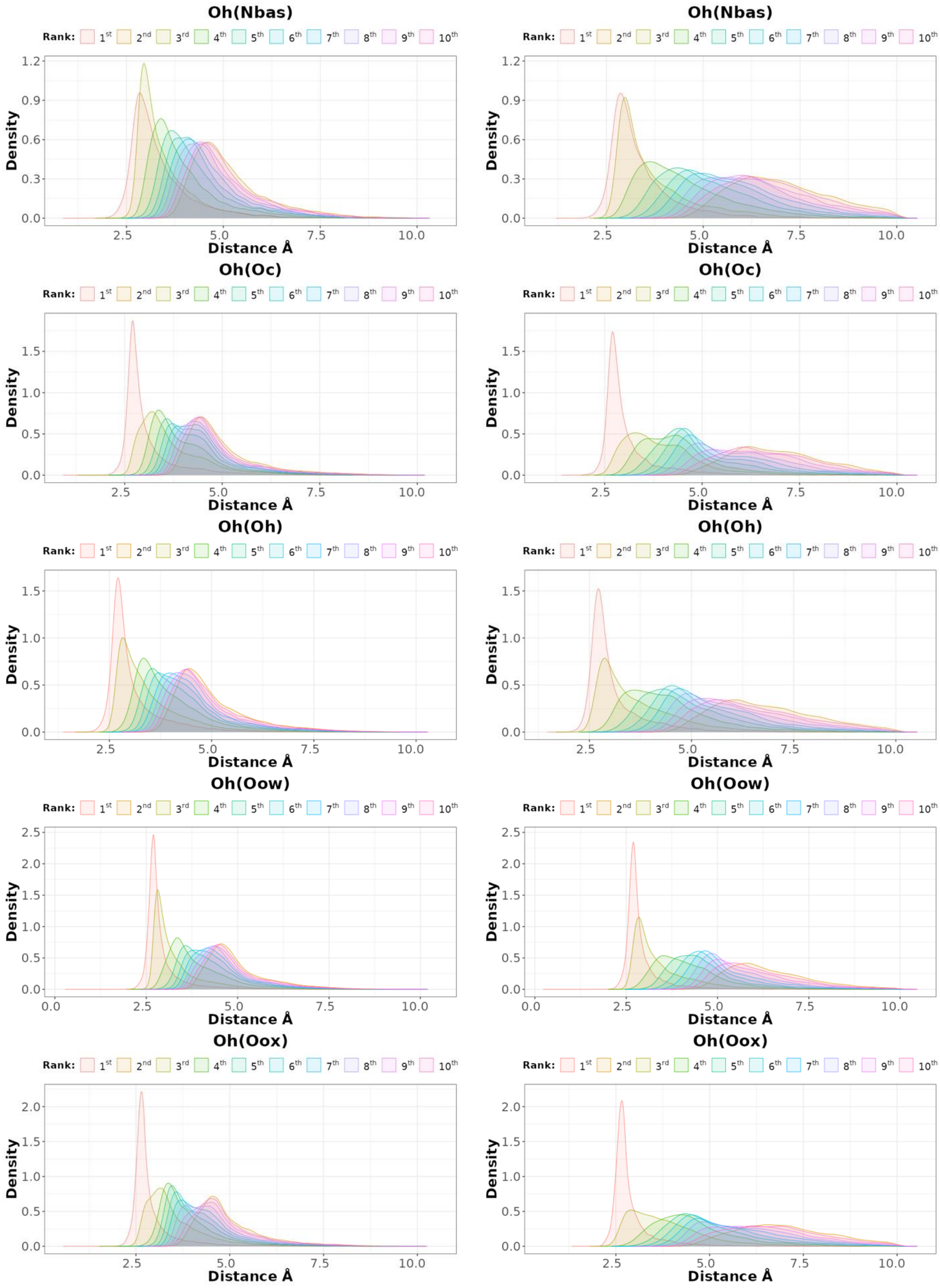

Oh(Nbas)
Rank: 1st 2nd 3rd 4th 5th 6th 7th 8th 9th 10th
Density
Distance Å
Oh(Nbas)
Rank: 1st 2nd 3rd 4th 5th 6th 7th 8th 9th 10th
Density
Distance Å
Oh(Oc)
Rank: 1st 2nd 3rd 4th 5th 6th 7th 8th 9th 10th
Density
Distance Å
Oh(Oc)
Rank: 1st 2nd 3rd 4th 5th 6th 7th 8th 9th 10th
Density
Distance Å
Oh(Oh)
Rank: 1st 2nd 3rd 4th 5th 6th 7th 8th 9th 10th
Density
Distance Å
Oh(Oh)
Rank: 1st 2nd 3rd 4th 5th 6th 7th 8th 9th 10th
Density
Distance Å
Oh(Oow)
Rank: 1st 2nd 3rd 4th 5th 6th 7th 8th 9th 10th
Density
Distance Å
Oh(Oow)
Rank: 1st 2nd 3rd 4th 5th 6th 7th 8th 9th 10th
Density
Distance Å
Oh(Oox)
Rank: 1st 2nd 3rd 4th 5th 6th 7th 8th 9th 10th
Density
Distance Å
Oh(Oox)
Rank: 1st 2nd 3rd 4th 5th 6th 7th 8th 9th 10th
Density
Distance Å

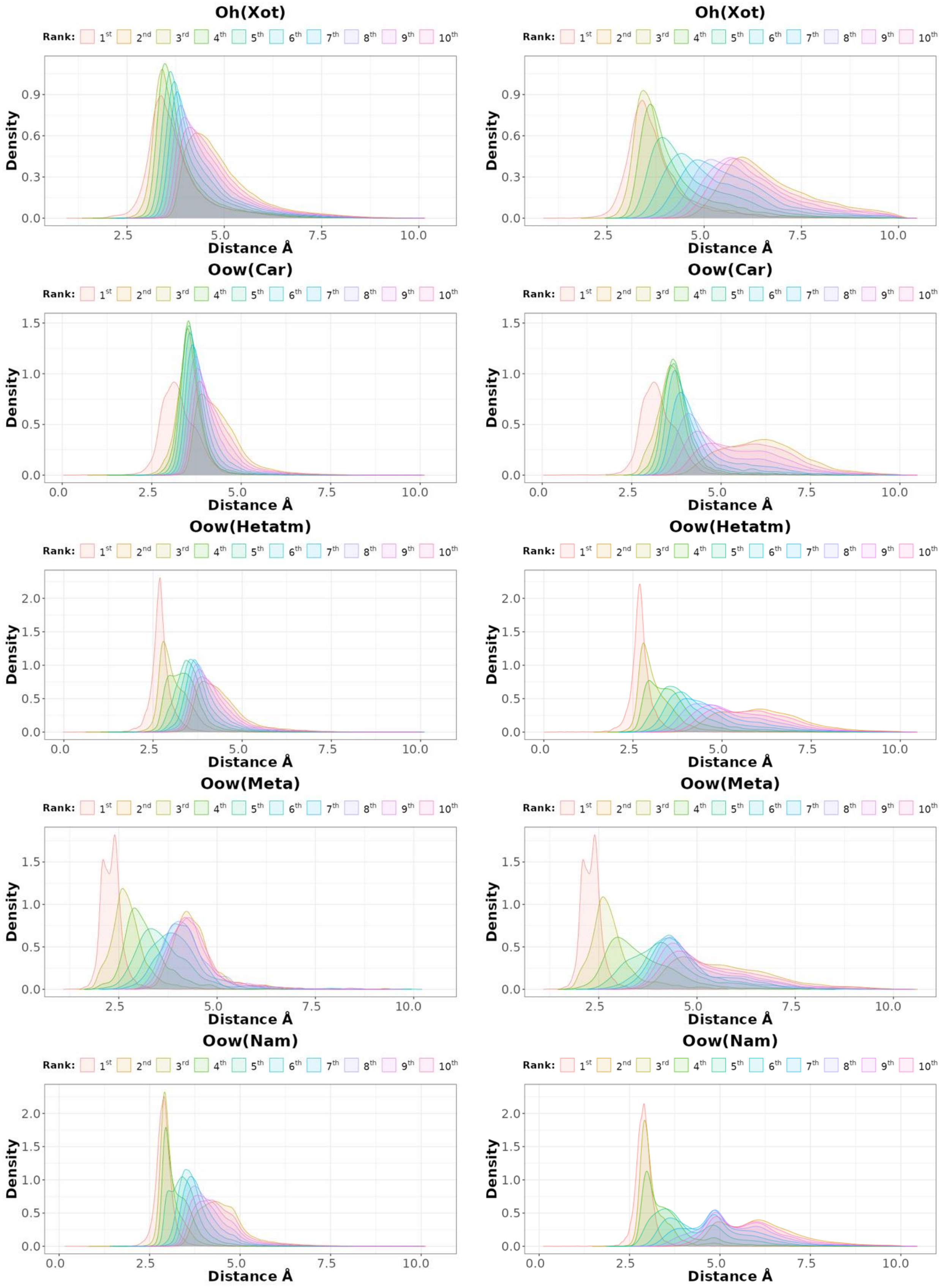
Oh(Xot)
Oh(Xot)
Oow(Car)
Oow(Car)
Oow(Hetatm)
Oow(Hetatm)
Oow(Meta)
Oow(Meta)
Oow(Nam)
Oow(Nam)
Rank: 1st 2nd 3rd 4th 5th 6th 7th 8th 9th 10th
Density
Distance Å

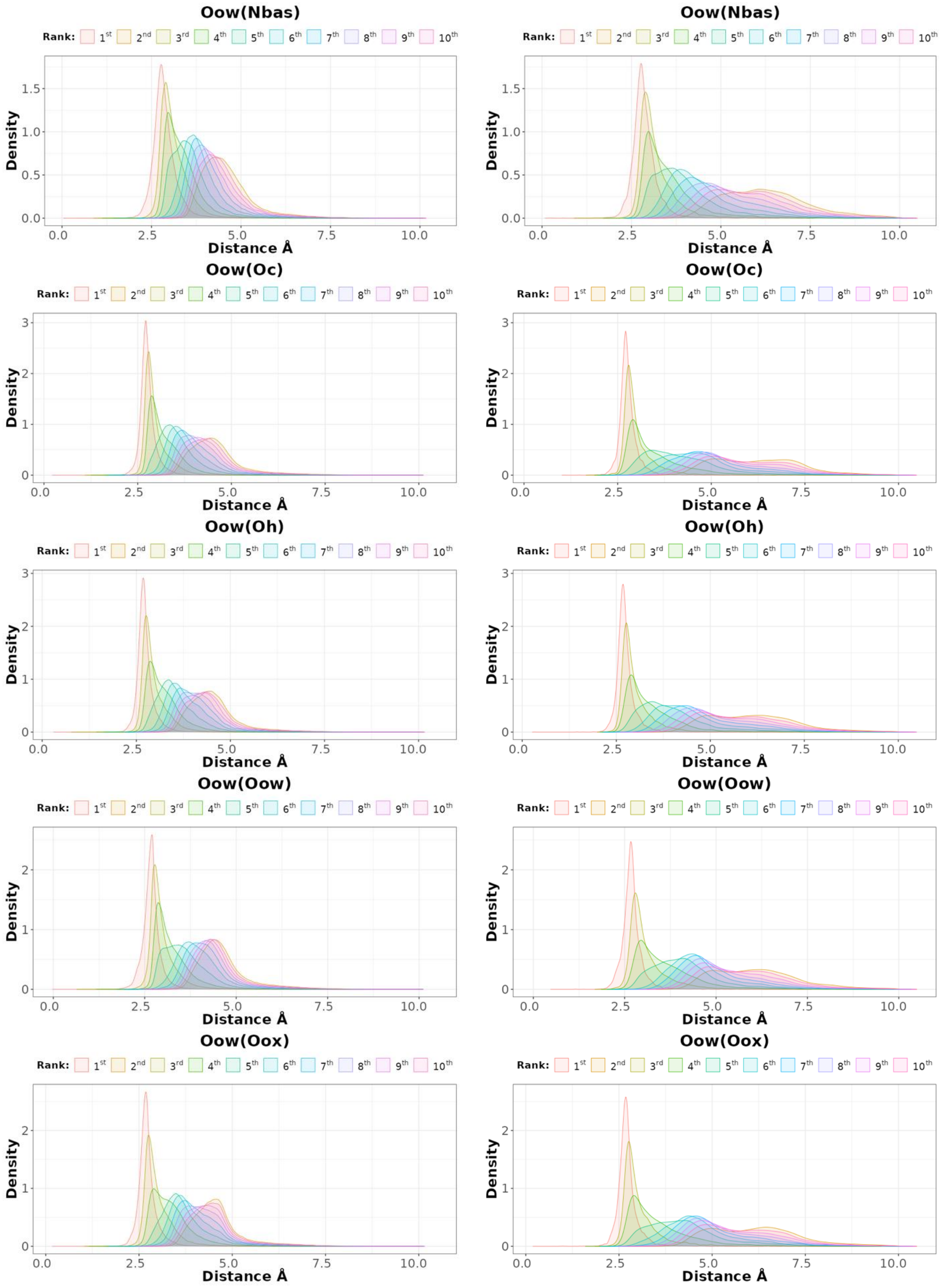
Oow(Nbas)
Oow(Nbas)
Oow(Oc)
Oow(Oc)
Oow(Oh)
Oow(Oh)
Oow(Oow)
Oow(Oow)
Oow(Oox)
Oow(Oox)
Rank: 1st 2nd 3rd 4th 5th 6th 7th 8th 9th 10th
Density
Distance Å

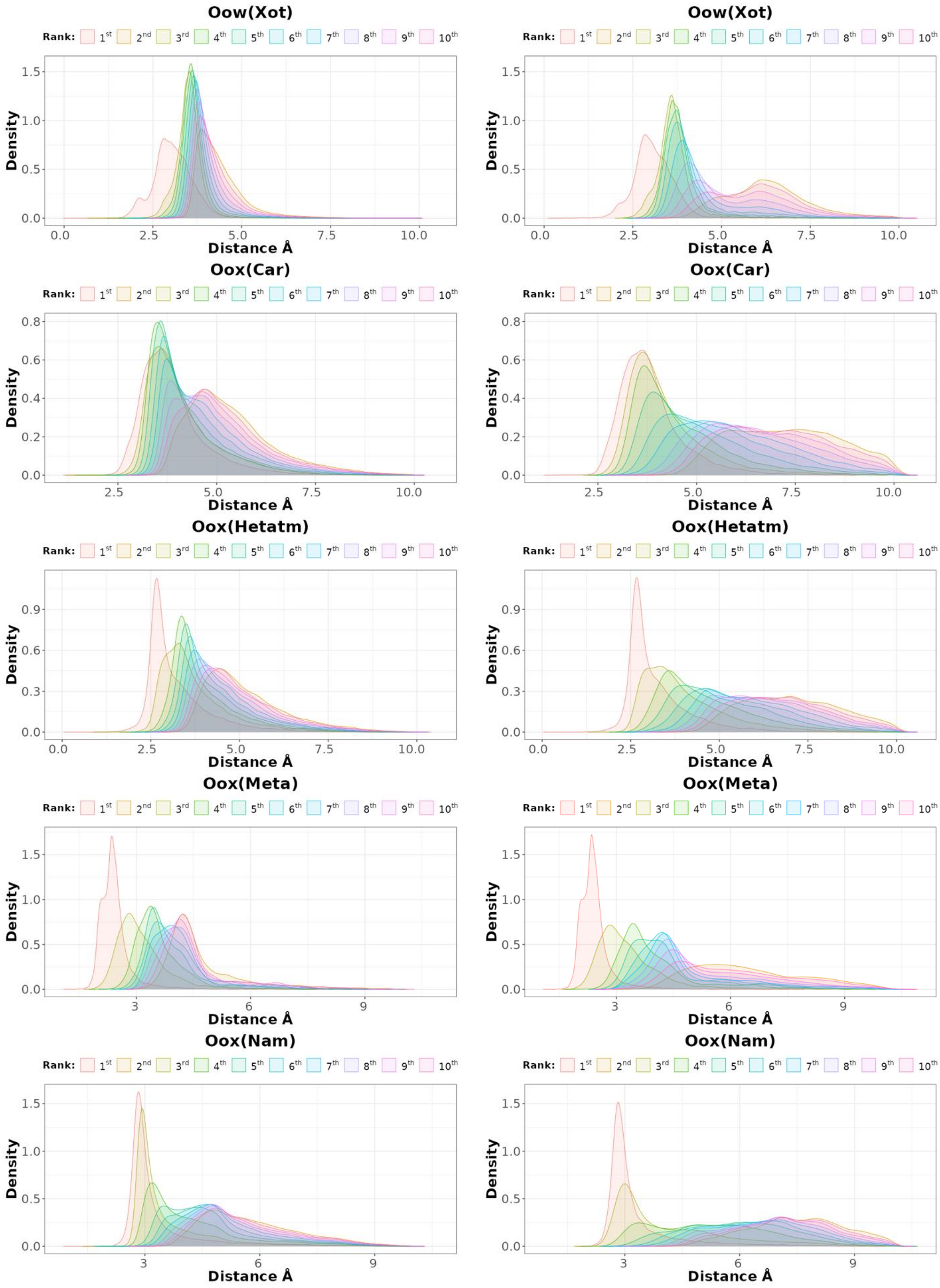
Oow(Xot)
Oow(Xot)
Oox(Car)
Oox(Car)
Oox(Hetatm)
Oox(Hetatm)
Oox(Meta)
Oox(Meta)
Oox(Nam)
Oox(Nam)
Rank: 1st 2nd 3rd 4th 5th 6th 7th 8th 9th 10th
Density
Distance Å

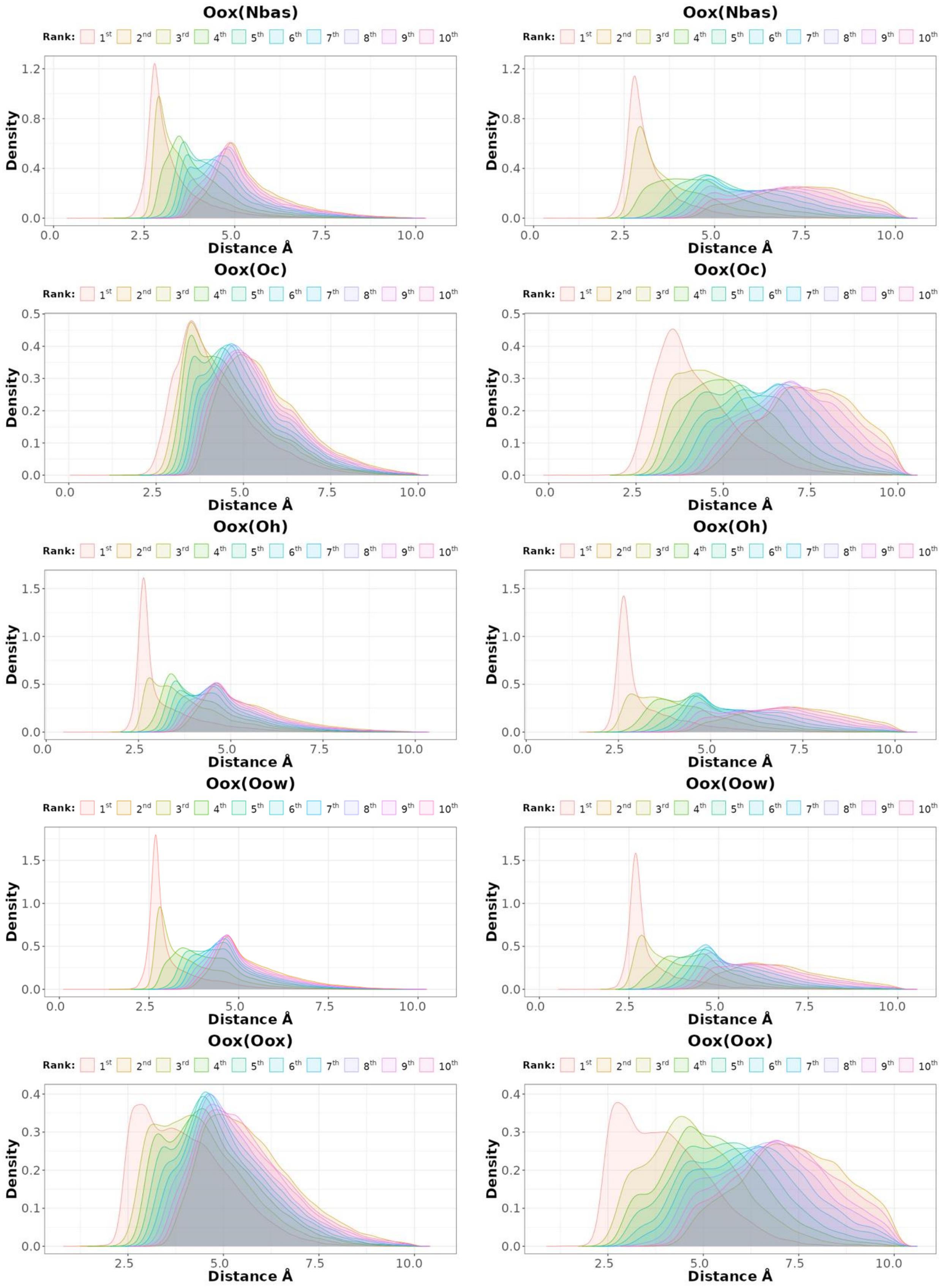
Oox(Nbas)
Oox(Nbas)
Oox(Oc)
Oox(Oc)
Oox(Oh)
Oox(Oh)
Oox(Oow)
Oox(Oow)
Oox(Oox)
Oox(Oox)
Rank: 1st 2nd 3rd 4th 5th 6th 7th 8th 9th 10th
Density
Distance Å

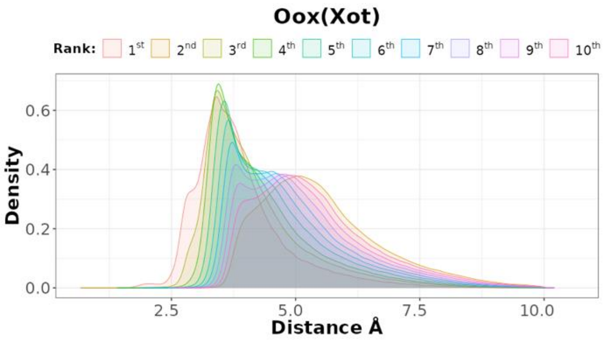
Oox(Xot)
Rank: 1st 2nd 3rd 4th 5th 6th 7th 8th 9th 10th
Density
0.0
0.2
0.4
0.6
2.5
5.0
7.5
10.0
Distance Å

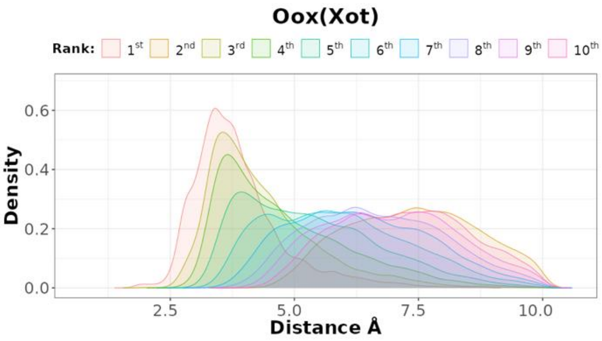
Oox(Xot)
Rank: 1st 2nd 3rd 4th 5th 6th 7th 8th 9th 10th
Density
0.0
0.2
0.4
0.6
2.5
5.0
7.5
10.0
Distance Å

Densities that are less well stratified: Meta, Hetatm, Nam and Xot.

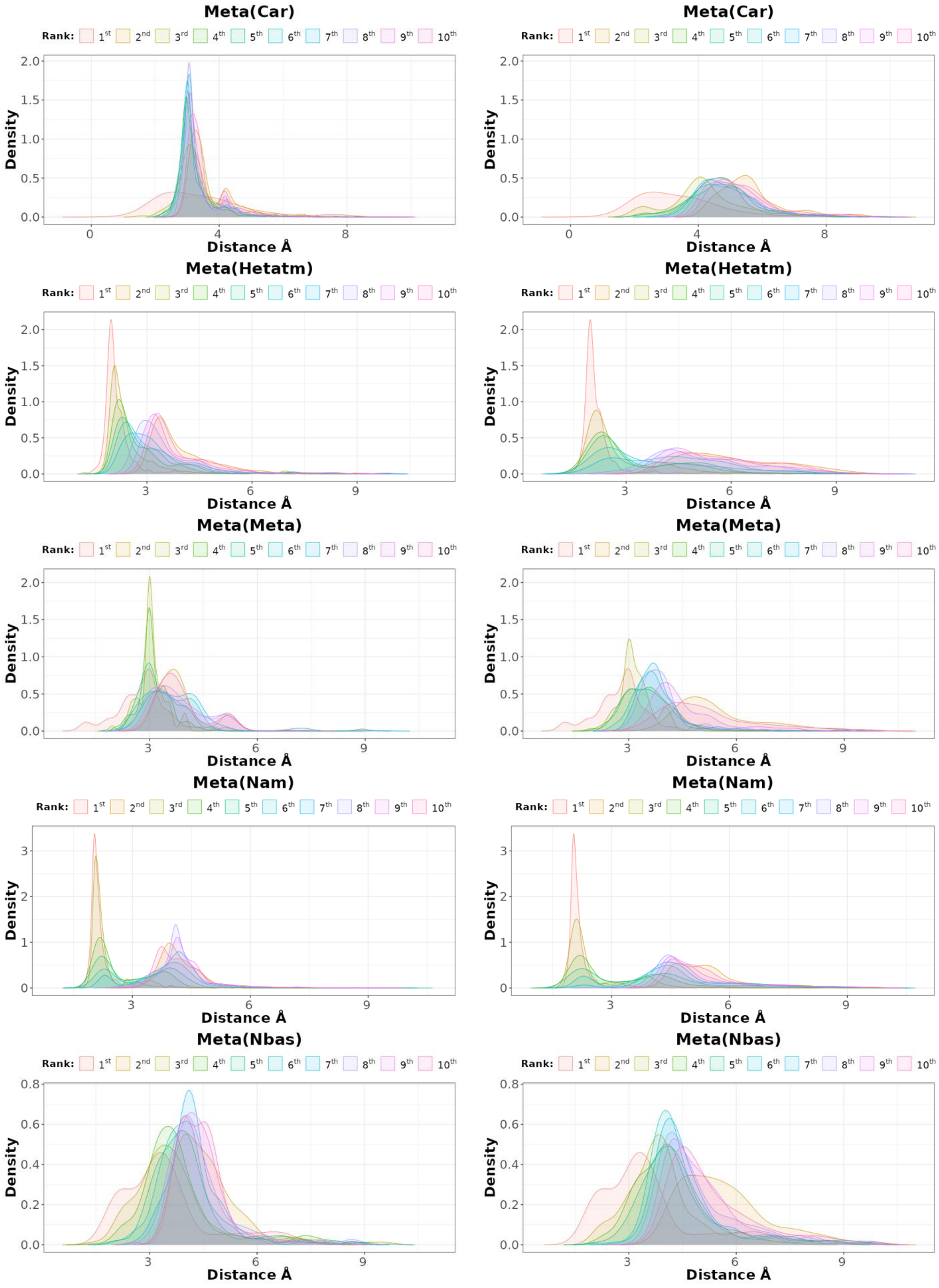

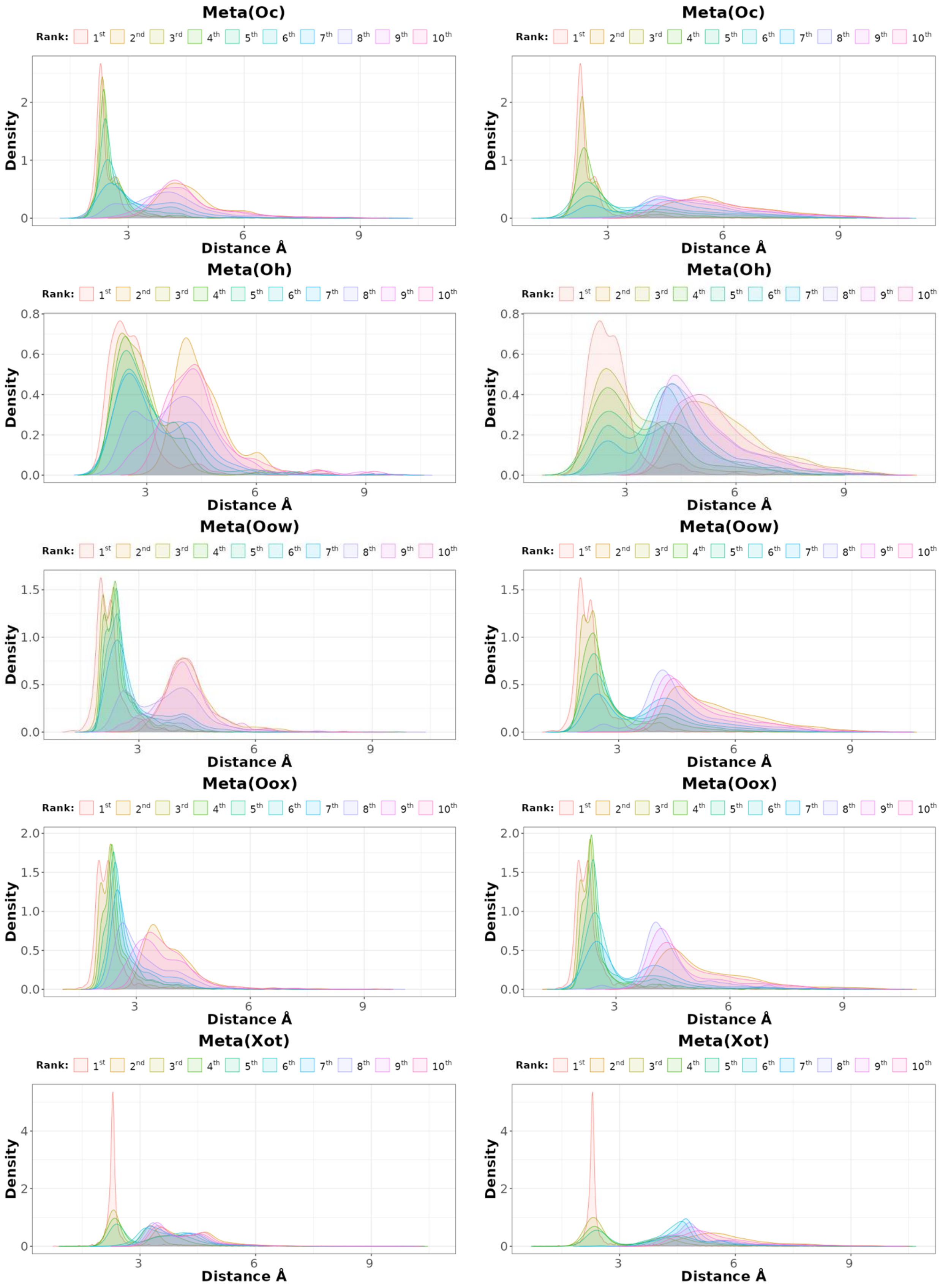

Meta(Oc)
Rank: 1st 2nd 3rd 4th 5th 6th 7th 8th 9th 10th
Density
Distance Å
Meta(Oh)
Meta(Oow)
Meta(Oox)
Meta(Xot)

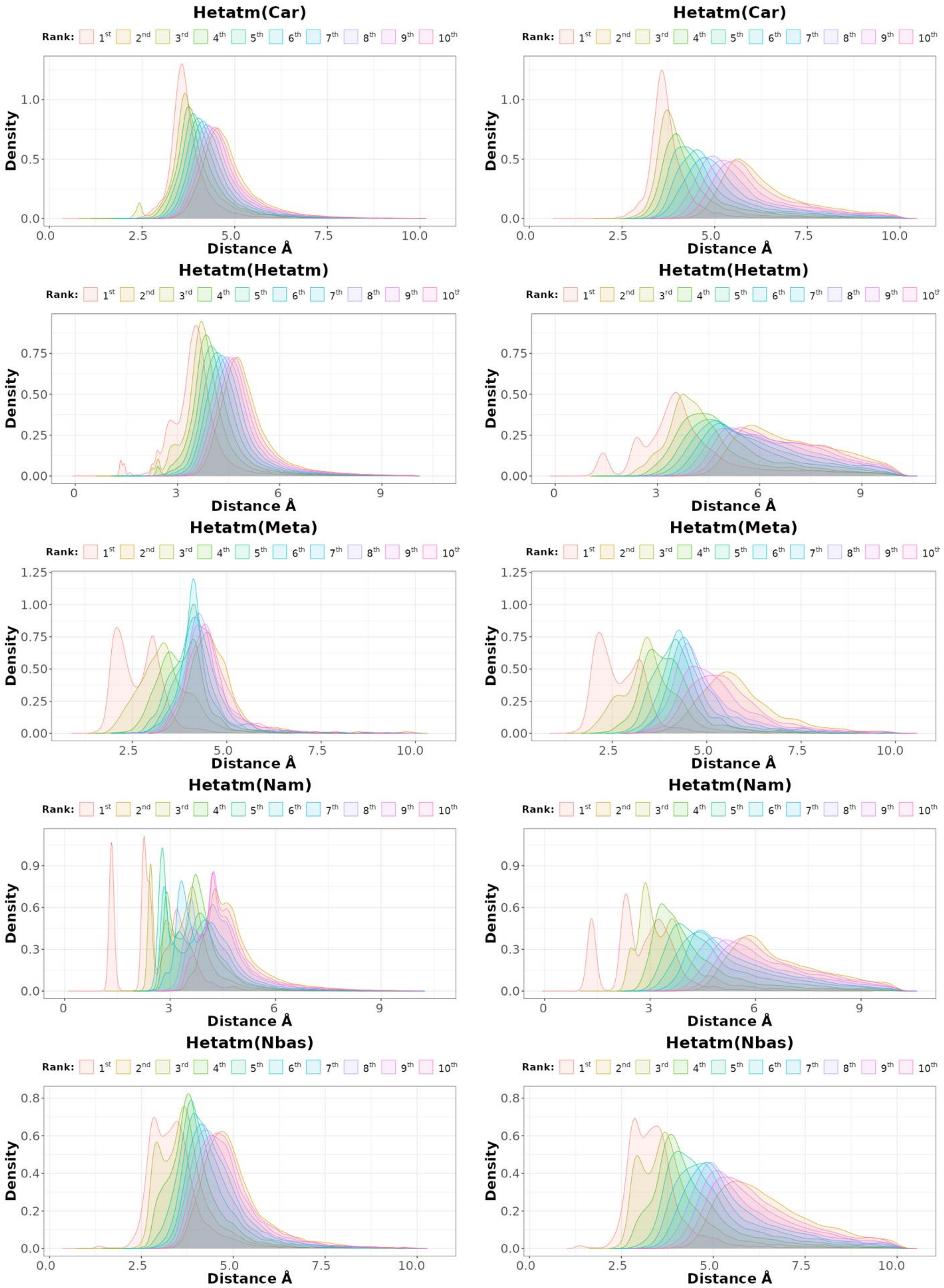
Hetatm(Car)
Rank: 1st 2nd 3rd 4th 5th 6th 7th 8th 9th 10th
Density
Distance Å
Hetatm(Car)
Rank: 1st 2nd 3rd 4th 5th 6th 7th 8th 9th 10th
Density
Distance Å
Hetatm(Hetatm)
Rank: 1st 2nd 3rd 4th 5th 6th 7th 8th 9th 10th
Density
Distance Å
Hetatm(Hetatm)
Rank: 1st 2nd 3rd 4th 5th 6th 7th 8th 9th 10th
Density
Distance Å
Hetatm(Meta)
Rank: 1st 2nd 3rd 4th 5th 6th 7th 8th 9th 10th
Density
Distance Å
Hetatm(Meta)
Rank: 1st 2nd 3rd 4th 5th 6th 7th 8th 9th 10th
Density
Distance Å
Hetatm(Nam)
Rank: 1st 2nd 3rd 4th 5th 6th 7th 8th 9th 10th
Density
Distance Å
Hetatm(Nam)
Rank: 1st 2nd 3rd 4th 5th 6th 7th 8th 9th 10th
Density
Distance Å
Hetatm(Nbas)
Rank: 1st 2nd 3rd 4th 5th 6th 7th 8th 9th 10th
Density
Distance Å
Hetatm(Nbas)
Rank: 1st 2nd 3rd 4th 5th 6th 7th 8th 9th 10th
Density
Distance Å

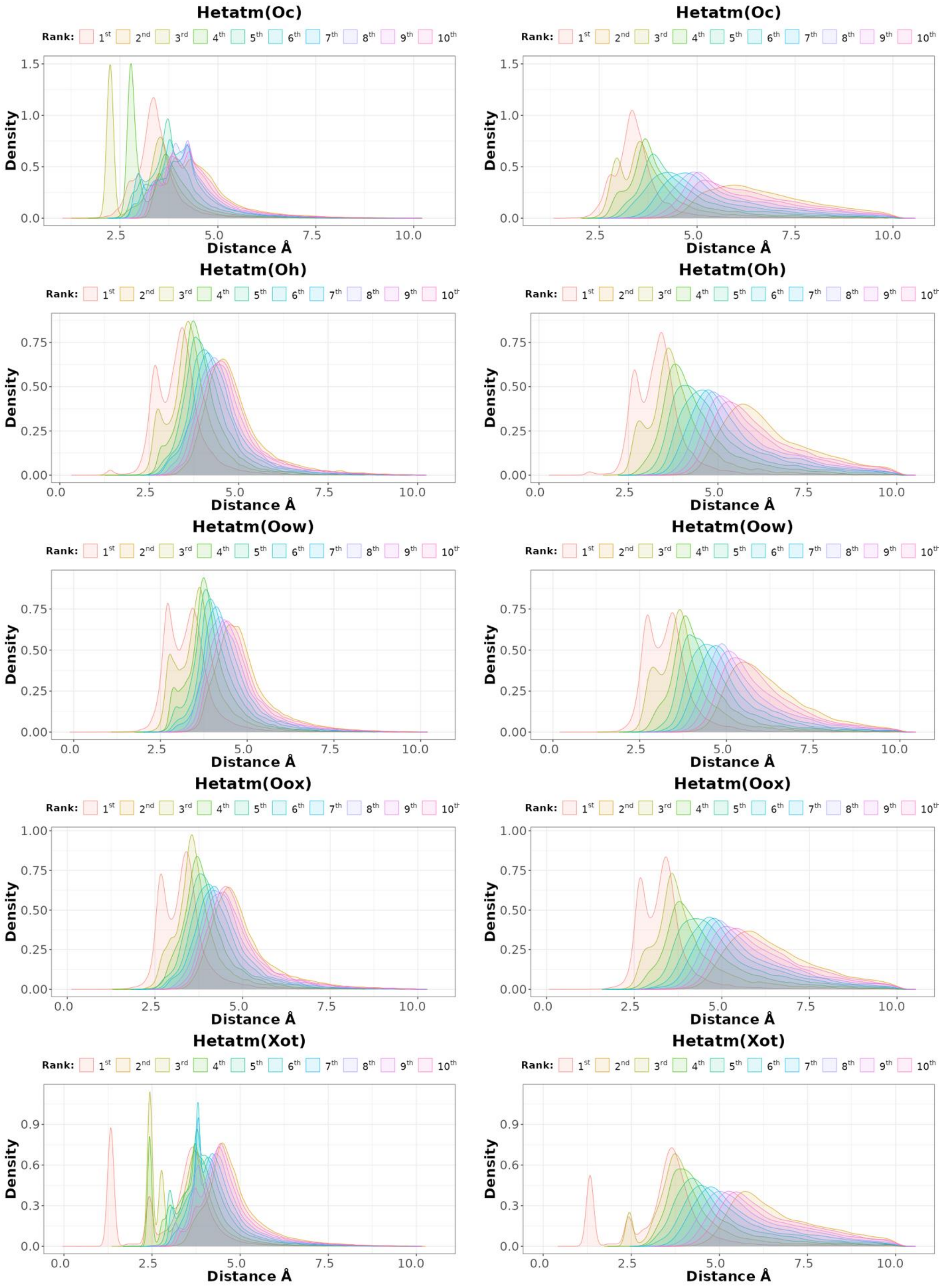
Hetatm(Oc)
Hetatm(Oh)
Hetatm(Oow)
Hetatm(Oox)
Hetatm(Xot)
Rank: 1st 2nd 3rd 4th 5th 6th 7th 8th 9th 10th
Density
Distance Å

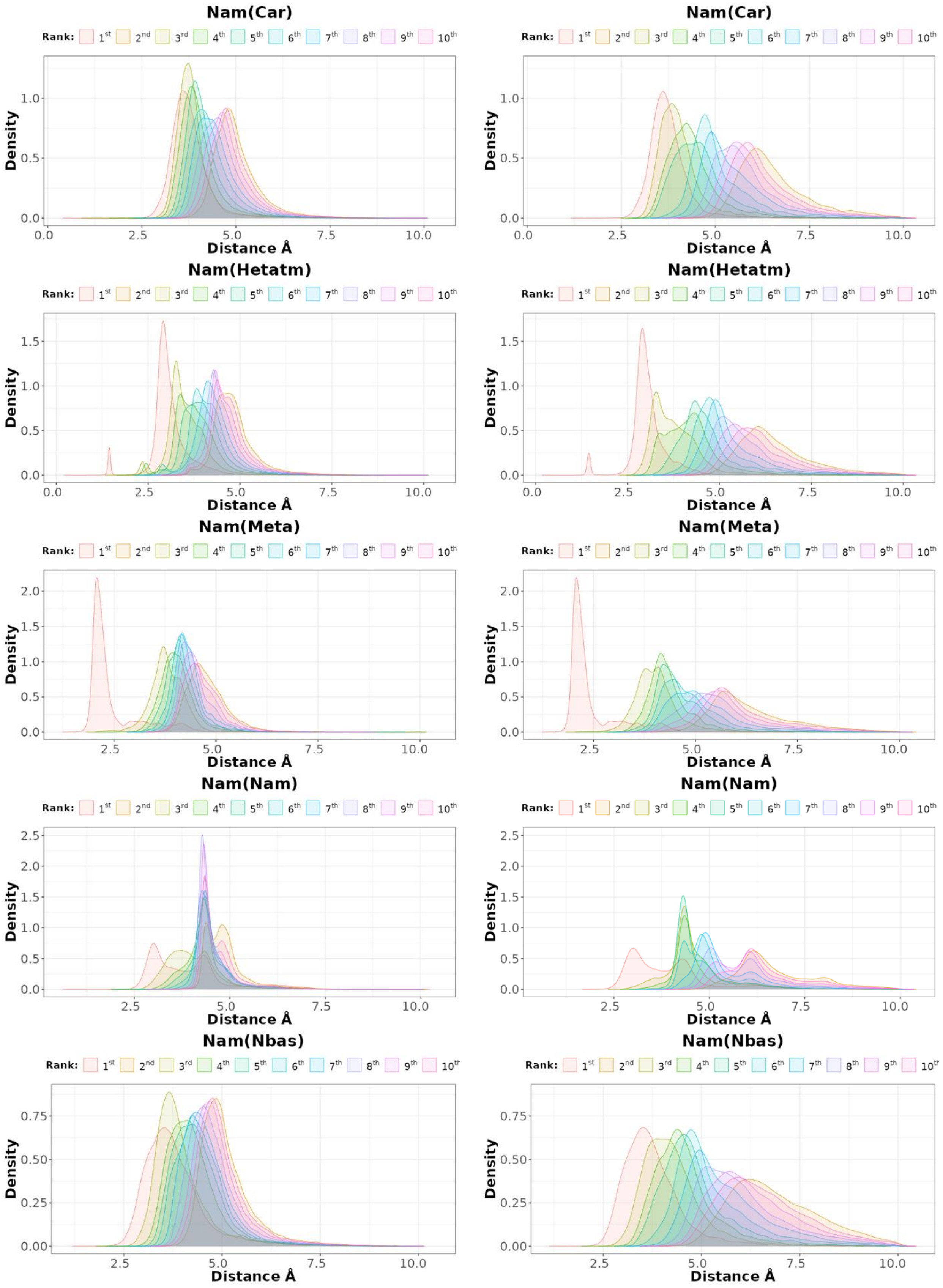
Nam(Car)
Nam(Car)
Nam(Hetatm)
Nam(Hetatm)
Nam(Meta)
Nam(Meta)
Nam(Nam)
Nam(Nam)
Nam(Nbas)
Nam(Nbas)
Rank: 1st 2nd 3rd 4th 5th 6th 7th 8th 9th 10th
Density
Distance Å

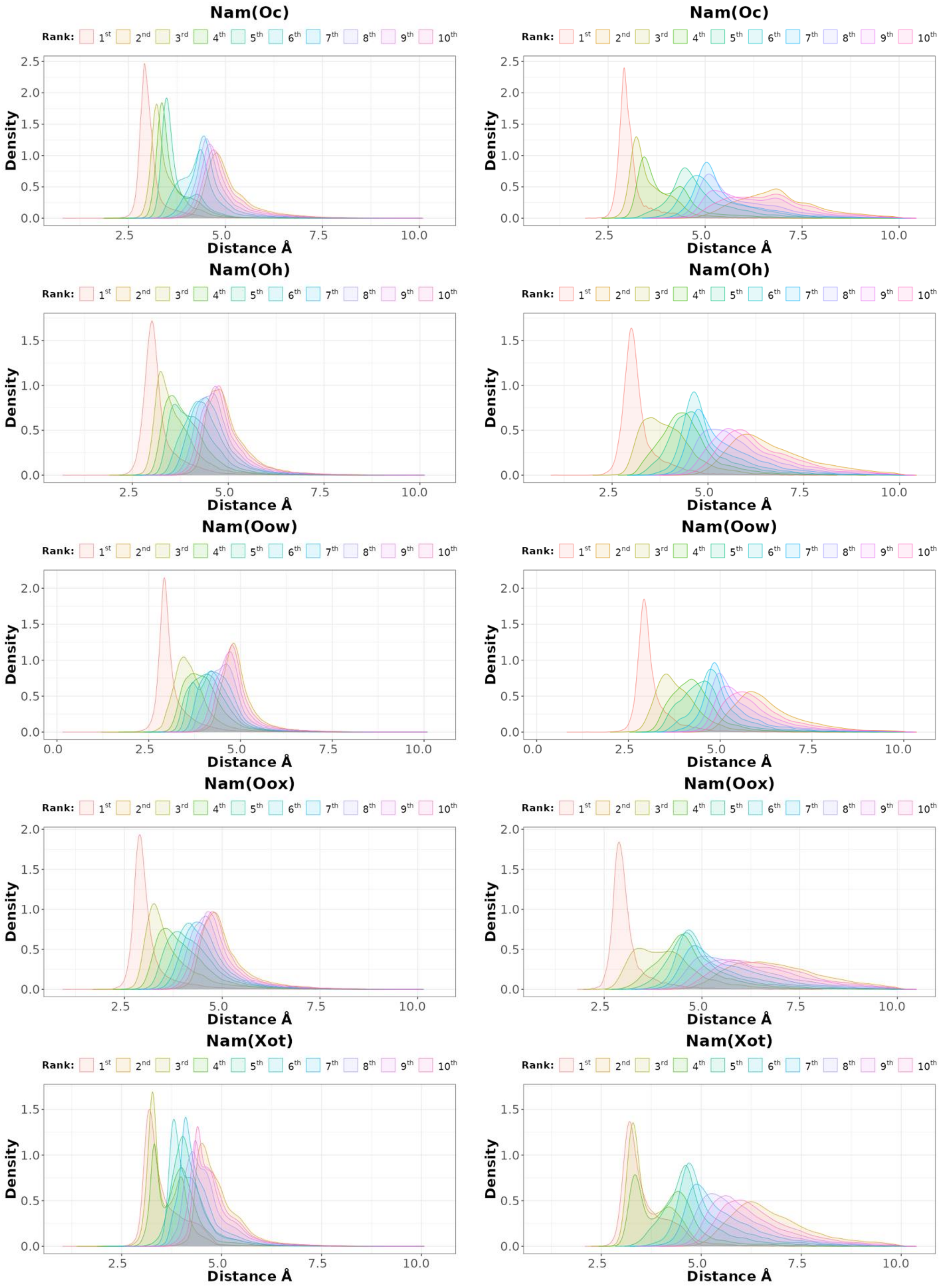

Nam(Oc)
Nam(Oc)
Nam(Oh)
Nam(Oh)
Nam(Oow)
Nam(Oow)
Nam(Oox)
Nam(Oox)
Nam(Xot)
Nam(Xot)
Rank: 1st 2nd 3rd 4th 5th 6th 7th 8th 9th 10th
Density
Distance Å

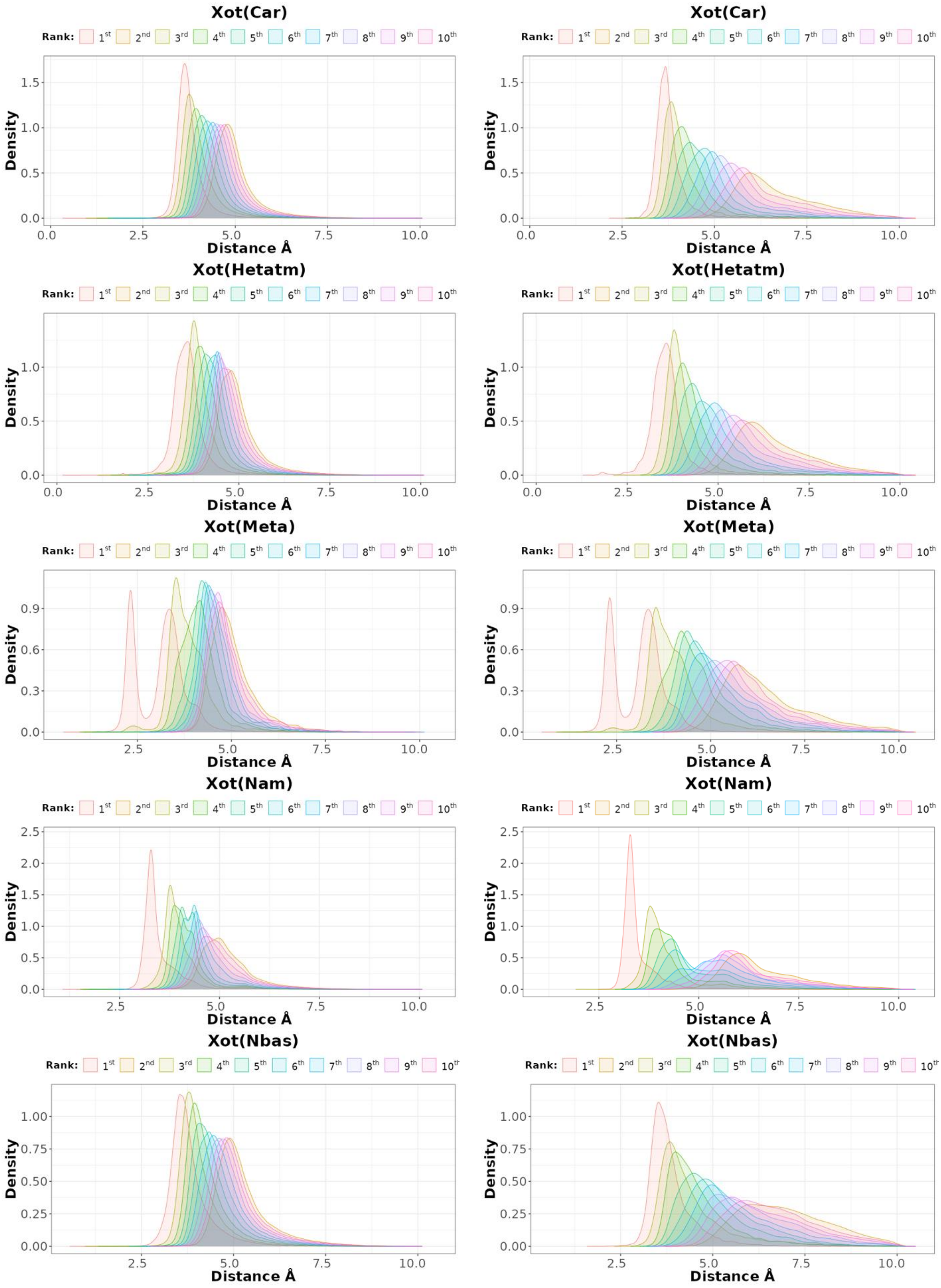

Xot(Car)
Xot(Car)
Rank: 1st 2nd 3rd 4th 5th 6th 7th 8th 9th 10th
Density
Distance Å
Xot(Hetatm)
Xot(Hetatm)
Xot(Meta)
Xot(Meta)
Xot(Nam)
Xot(Nam)
Xot(Nbas)
Xot(Nbas)

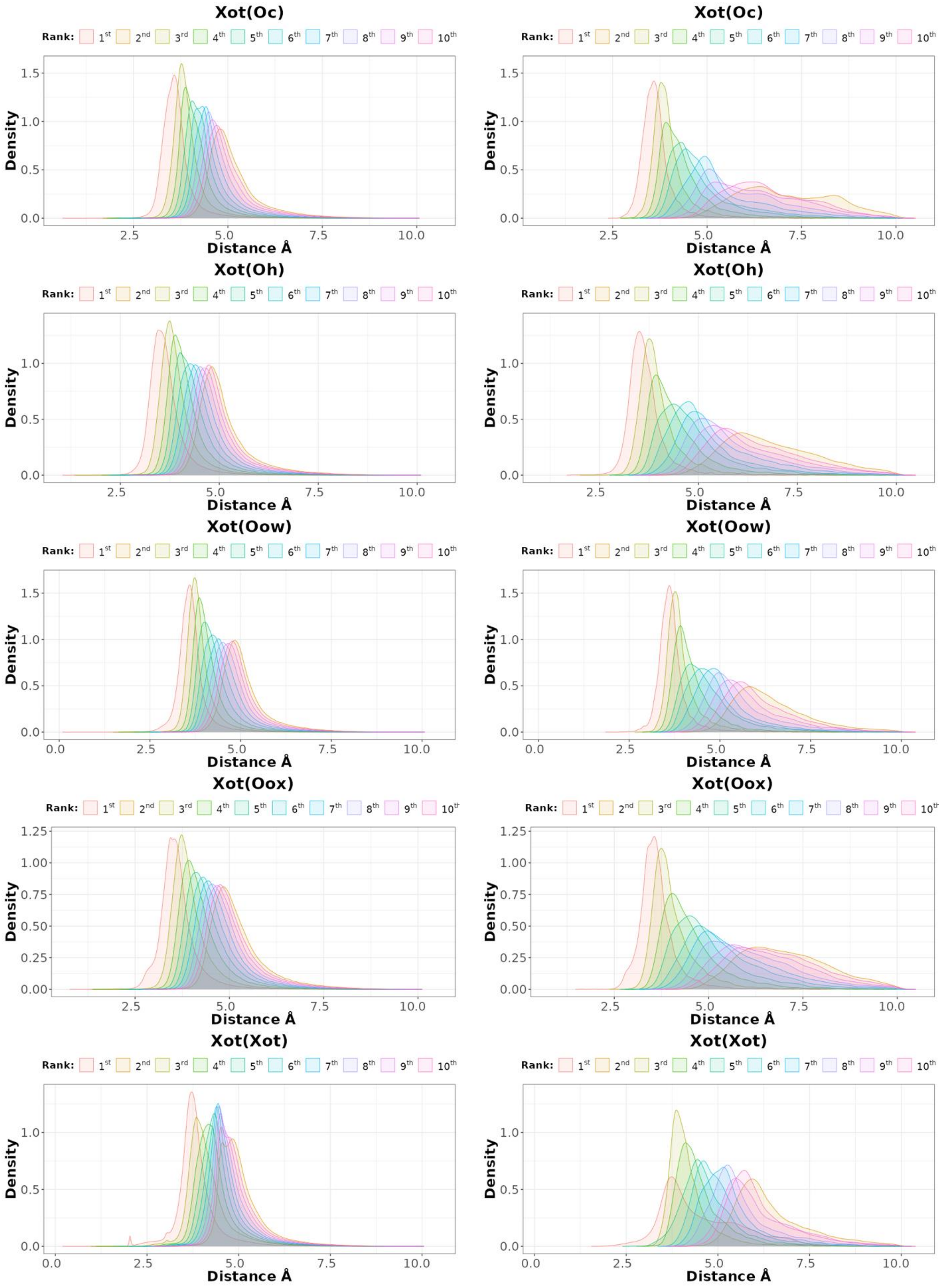

Xot(Oc)
Rank: 1st 2nd 3rd 4th 5th 6th 7th 8th 9th 10th
Density
Distance Å
Xot(Oc)
Rank: 1st 2nd 3rd 4th 5th 6th 7th 8th 9th 10th
Density
Distance Å
Xot(Oh)
Rank: 1st 2nd 3rd 4th 5th 6th 7th 8th 9th 10th
Density
Distance Å
Xot(Oh)
Rank: 1st 2nd 3rd 4th 5th 6th 7th 8th 9th 10th
Density
Distance Å
Xot(Oow)
Rank: 1st 2nd 3rd 4th 5th 6th 7th 8th 9th 10th
Density
Distance Å
Xot(Oow)
Rank: 1st 2nd 3rd 4th 5th 6th 7th 8th 9th 10th
Density
Distance Å
Xot(Oox)
Rank: 1st 2nd 3rd 4th 5th 6th 7th 8th 9th 10th
Density
Distance Å
Xot(Oox)
Rank: 1st 2nd 3rd 4th 5th 6th 7th 8th 9th 10th
Density
Distance Å
Xot(Xot)
Rank: 1st 2nd 3rd 4th 5th 6th 7th 8th 9th 10th
Density
Distance Å
Xot(Xot)
Rank: 1st 2nd 3rd 4th 5th 6th 7th 8th 9th 10th
Density
Distance Å

Supporting information 4. Benchmarking the $FS^3_{protein}$ against established scoring functions using the 3DRobot dataset.

Rank of the native X-ray structure among its 300 associated decoys for each of the five scoring functions benchmarked on the 3DRobot dataset (200 native-decoy sets). A rank of 1 indicates that the native structure received the best score among all 301 structures (native + 300 decoys). Each box represents the interquartile range across the 200 native-decoy sets; the horizontal line within each box shows the median rank; whiskers extend to 1.5 times the interquartile range; individual points beyond the whiskers are shown as outliers.

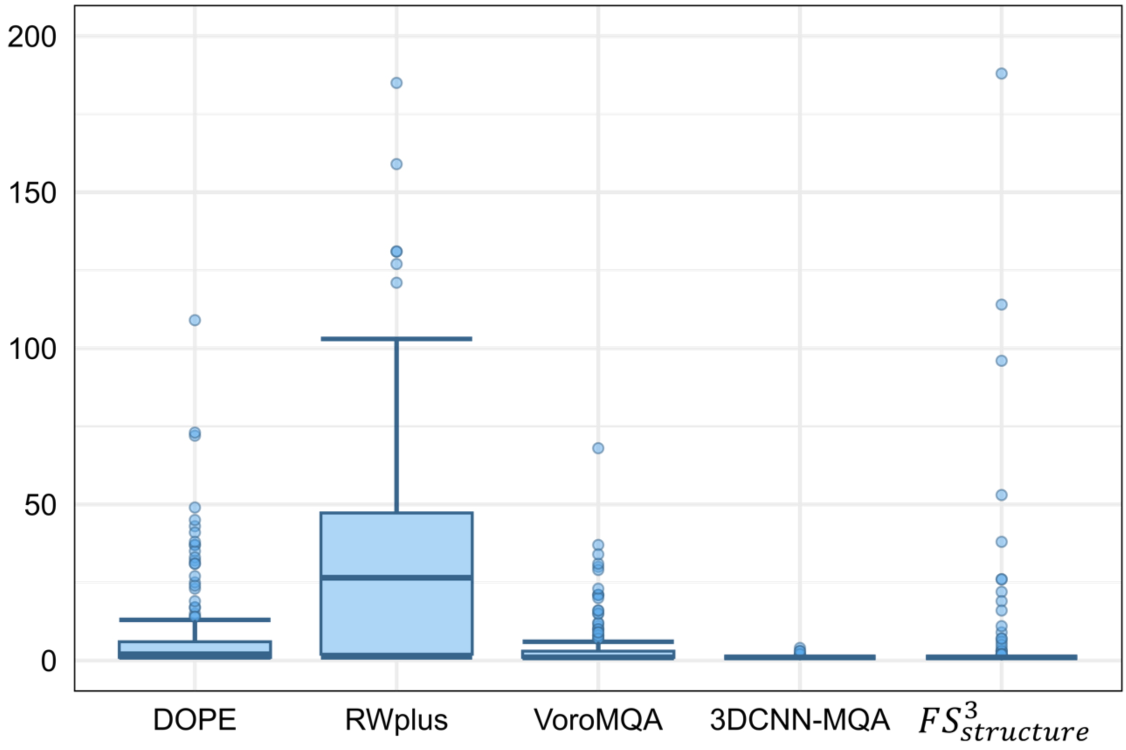

Benchmarking against other scoring functions, full plots. The $FS^k_{structure}$ score was calculated using $k = 3$ with the primary contact filter applied. In all cases, the score of each native X-ray structure was set to 0 (red point), and scaling was done so that more positive values indicate better scores. The x-axis shows the RMSD of each decoy to native; densities of presence are plotted as a color gradient.

Left column (next page): normalization within each native-decoy set, where unity is the standard deviation of the scores within that set; this normalization highlights the ability of each function to separate native X-ray structures from decoys.

Right column (next page): normalization over the complete dataset for each scoring function, where unity is the standard deviation of all scores combined; this normalization allows comparison of raw score distributions across scoring functions.

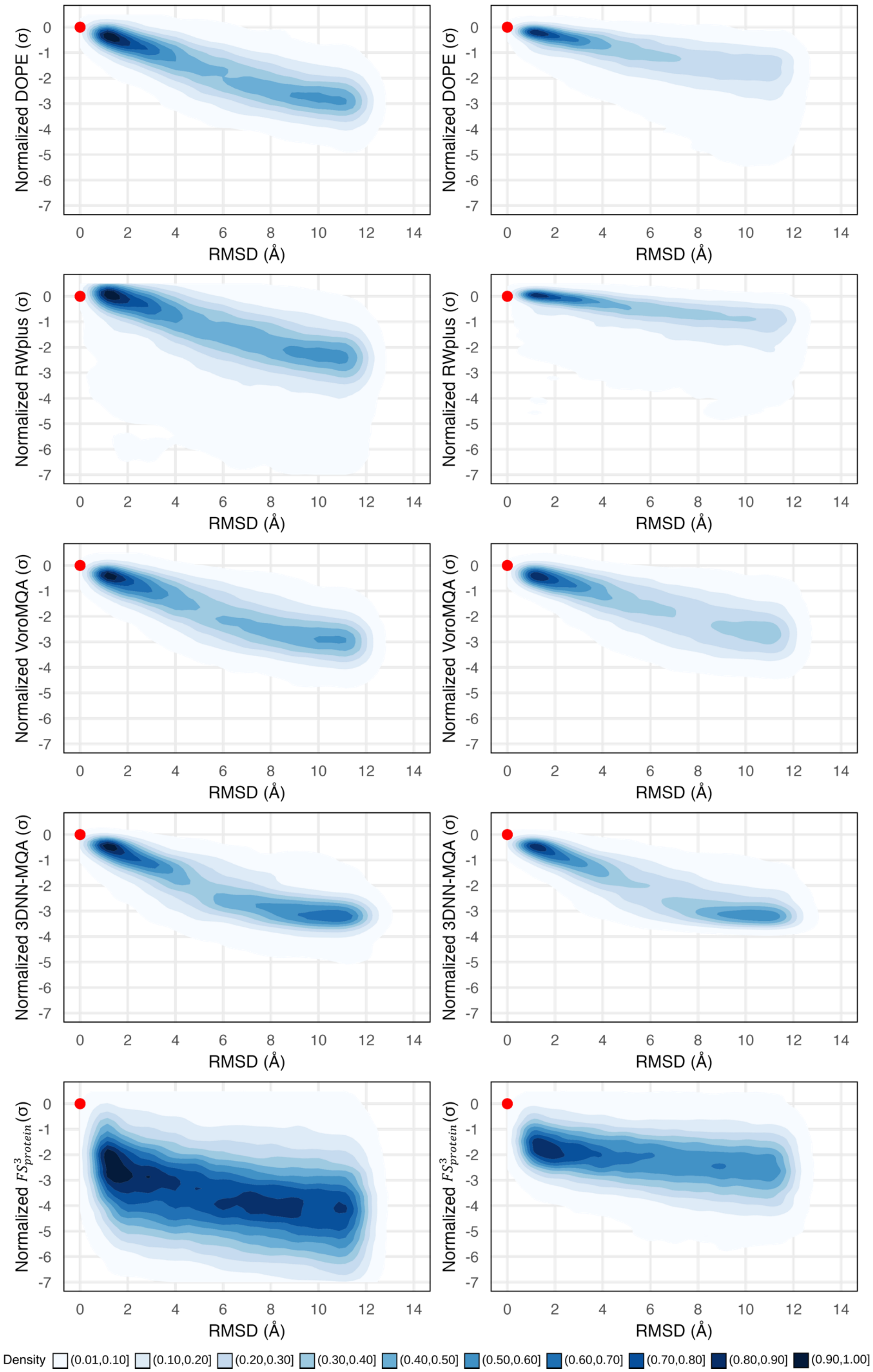
Normalized DOPE (σ)
Normalized RWplus (σ)
Normalized VoroMQA (σ)
Normalized 3DNN-MQA (σ)
Normalized $FS^3_{protein}$ (σ)
RMSD (Å)
0 -1 -2 -3 -4 -5 -6 -7
0 2 4 6 8 10 12 14
Density
(0.01,0.10]
(0.10,0.20]
(0.20,0.30]
(0.30,0.40]
(0.40,0.50]
(0.50,0.60]
(0.60,0.70]
(0.70,0.80]
(0.80,0.90]
(0.90,1.00]

Supporting information 5. Contact preferences matrices for the 8 protein atom type origins at the 10 ranks. The neighbor list is collected without (left-hand panels) or with (right-hand panels) the *primary contact* filter activated.

Rank 1 — f_{obs}/f_{exp} (scale 0 1 2 3 4 5 6)

	Hetatm	Meta	Xot	Car	Nam	Nbas	Oc	Oh	Oox	Oow
Xot	0.4	0.5	0.9	1.2	1	0.6	1.2	0.9	0.9	0.8
Car	0.6	2	2.3	2.4	0.3	1.6	0.6	1.2	1.1	0.9
Nam	0.4	0.7	0.9	0.4	0.1	0.3	1.9	0.7	0.9	0.7
Nbas	0.7	0.1	1	0.9	0.2	1	0.7	1.1	4.9	1.6
Oc	0.3	0.7	0.7	0.5	3.2	1.3	0.1	1	0.2	1.1
Oh	0.5	0.7	0.9	0.6	0.5	1.1	0.9	1.4	2.5	1.6
Oox	0.4	4.9	1.3	0.6	0.7	5.5	0.3	2.2	0.6	1.9
Oow	0.4	2.2	0.2	0.1	0.5	1.8	0.7	1.6	2.2	2.5

Rank 1 — f_{obs}/f_{exp} (scale 0 1 2 3 4 5 6)

	Hetatm	Meta	Xot	Car	Nam	Nbas	Oc	Oh	Oox	Oow
Xot	0.4	0.5	0.9	1.2	1	0.6	1.2	0.9	0.9	0.8
Car	0.6	2	2.3	2.4	0.3	1.6	0.6	1.2	1.1	0.9
Nam	0.4	0.7	0.9	0.4	0.1	0.3	1.9	0.7	0.9	0.7
Nbas	0.7	0.1	1	0.9	0.2	1	0.7	1.1	4.9	1.6
Oc	0.3	0.7	0.7	0.5	3.2	1.3	0.1	1	0.2	1.1
Oh	0.5	0.7	0.9	0.6	0.5	1.1	0.9	1.4	2.5	1.6
Oox	0.4	4.9	1.3	0.6	0.7	5.5	0.3	2.2	0.6	1.9
Oow	0.4	2.2	0.2	0.1	0.5	1.8	0.7	1.6	2.2	2.5

Rank 2 — f_{obs}/f_{exp} (scale 0 1 2 3 4 5 6)

	Empty	Hetatm	Meta	Xot	Car	Nam	Nbas	Oc	Oh	Oox	Oow
Xot	0.2	0.4	0.8	0.8	1.1	1	0.8	1.3	1.1	1	1
Car	0.5	0.6	2.2	1.4	1.7	0.5	1.4	0.6	1.1	1.2	0.9
Nam	0.2	0.4	0.6	1.6	0.6	0.2	0.4	1.4	0.6	0.5	0.5
Nbas	4.8	0.7	0.5	0.8	1.8	0.3	1	0.7	1.2	4.6	1.5
Oc	0.2	0.3	0.3	1.1	0.6	2.9	0.8	0.4	0.7	0.4	0.6
Oh	0.5	0.5	0.7	1.1	1.2	1	1.5	0.6	1.3	1.3	1.3
Oox	0.5	0.4	3.1	1.1	1	0.9	4.7	0.4	1.5	0.8	1.5
Oow	0.8	0.4	1.2	0.5	0.5	0.8	2.2	0.7	1.6	2.2	2.6

Rank 2 — f_{obs}/f_{exp} (scale 0 1 2 3 4 5 6)

	Empty	Hetatm	Meta	Xot	Car	Nam	Nbas	Oc	Oh	Oox	Oow
Xot	0.2	0.4	0.7	0.8	1.1	0.9	0.8	1.3	1.1	1	1
Car	0.8	0.5	1.9	1.5	1.8	0.5	1.3	0.7	1.2	1.3	1
Nam	0.2	0.3	1	1.5	0.7	0.4	0.6	1.3	0.7	0.5	0.7
Nbas	6.2	0.6	0.6	0.9	1	0.4	1.3	0.8	1.4	4.3	1.6
Oc	0.3	0.3	0.4	0.9	0.6	2.9	0.7	0.4	0.5	0.4	0.6
Oh	0.9	0.5	0.7	1.1	1	1	1.5	0.7	1.3	1.2	1.3
Oox	0.9	0.4	3	1	0.7	0.9	4	0.5	1.7	1	1.6
Oow	0.3	0.4	1.1	0.5	0.4	0.7	2	0.7	1.6	2.1	2.4

Rank 3 — f_{obs}/f_{exp} (scale 0 1 2 3 4 5 6)

	Empty	Hetatm	Meta	Xot	Car	Nam	Nbas	Oc	Oh	Oox	Oow
Xot	0.2	0.4	0.9	0.9	1	1.1	0.9	1.1	1.1	1	1.1
Car	0.7	0.6	1.5	1.2	1.5	0.6	1.5	0.6	1.1	1.2	1
Nam	0.3	0.4	1	1.1	0.6	0.4	0.5	1.8	0.9	0.6	0.6
Nbas	5.9	0.7	0.6	0.9	1.9	0.4	1.1	0.6	1.1	4.2	1.4
Oc	0.2	0.3	0.3	1.1	0.7	2.2	0.6	0.5	0.7	0.5	0.5
Oh	0.6	0.6	0.6	1.2	1.3	0.7	1.2	0.7	1	1.5	0.9
Oox	0.7	0.4	2.7	1.1	1.3	0.6	3.6	0.5	1.4	0.9	1.3
Oow	0.8	0.5	0.9	0.8	0.9	0.8	2.1	0.6	1.3	1.8	2.3

Rank 3 — f_{obs}/f_{exp} (scale 0 1 2 3 4 5 6)

	Empty	Hetatm	Meta	Xot	Car	Nam	Nbas	Oc	Oh	Oox	Oow
Xot	0.5	0.4	0.8	1	1.1	0.9	0.9	1.1	1	0.9	1
Car	1	0.5	1.1	1.4	1.5	0.5	1.2	0.8	1.2	1.3	1
Nam	0.5	0.3	1.6	1.2	0.7	0.7	0.9	1.3	0.8	0.7	0.9
Nbas	6.1	0.4	0.7	0.9	1	0.5	1.5	0.8	1.5	3.4	1.6
Oc	0.5	0.3	0.5	0.7	0.7	2.4	0.6	0.8	0.6	0.7	0.6
Oh	1	0.5	0.7	1.2	1.2	0.8	1.2	0.8	1.2	1.2	1
Oox	1.5	0.4	2.1	1	0.8	0.8	2.7	0.7	1.6	1.4	1.5
Oow	0.3	0.4	0.6	0.9	0.6	0.6	1.9	0.6	1.2	1.6	2.1

Rank 4 — f_{obs}/f_{exp} (scale 0 1 2 3 4 5 6)

	Empty	Hetatm	Meta	Xot	Car	Nam	Nbas	Oc	Oh	Oox	Oow
Xot	0.3	0.4	0.9	1	1	1.1	0.9	1	1.1	1	1.1
Car	0.8	0.6	1.2	1.1	1.4	0.6	1.5	0.7	1.1	1.2	1
Nam	0.3	0.4	1.4	0.9	0.7	0.5	0.6	2.1	0.9	0.7	0.8
Nbas	6.4	0.7	0.6	0.9	1.6	0.5	1.3	0.6	1.2	3.9	1.4
Oc	0.2	0.3	0.4	1.2	0.7	1.8	0.6	0.6	0.8	0.6	0.6
Oh	0.7	0.6	0.5	1.2	1.3	0.7	1.1	0.7	1.1	1.6	0.9
Oox	1	0.4	2.3	1.1	1.2	0.6	3.1	0.6	1.4	1	1.4
Oow	0.9	0.5	0.7	1.1	1.1	0.7	1.8	0.5	1	1.6	1.8

Rank 4 — f_{obs}/f_{exp} (scale 0 1 2 3 4 5 6)

	Empty	Hetatm	Meta	Xot	Car	Nam	Nbas	Oc	Oh	Oox	Oow
Xot	0.6	0.4	0.9	1.1	1	1	0.9	1.1	1	0.9	0.9
Car	1.2	0.5	1	1.3	1.4	0.6	1.1	0.9	1.2	1.3	1
Nam	0.6	0.4	1.6	0.9	0.9	1.5	1.1	0.8	1	0.8	1.1
Nbas	6	0.4	0.8	0.9	0.9	0.6	1.5	0.9	1.5	2.9	1.5
Oc	0.6	0.4	0.7	0.7	0.9	1.4	0.7	1.3	0.9	0.8	0.9
Oh	1.2	0.4	0.8	1.1	1	0.8	1.1	1	1.2	1.3	1.1
Oox	2.1	0.4	1.9	0.9	0.8	0.8	2.2	0.9	1.5	1.5	1.5
Oow	0.3	0.4	0.6	1.1	0.8	0.7	1.5	0.6	0.9	1.2	1.9

Rank 5

f_{obs}/f_{exp} (scale: 0 1 2 3 4 5 6)

	Empty	Hetatm	Meta	Xot	Car	Nam	Nbas	Oc	Oh	Oox	Oow
Xot	0.3	0.4	0.9	1	1	1.1	0.9	1.1	1	0.9	1
Car	0.9	0.6	1	1	1.5	0.7	1.4	0.9	1.1	1.2	1
Nam	0.3	0.4	1.4	1.1	0.7	0.9	0.7	1.1	0.9	0.7	1
Nbas	6.2	0.7	0.6	0.9	1.5	0.6	1.3	0.8	1.3	3.5	1.5
Oc	0.3	0.3	0.4	1.1	0.7	1.3	0.6	0.9	0.8	0.7	0.7
Oh	0.9	0.6	0.6	1.1	1.2	0.8	1.1	0.9	1.1	1.6	1
Oox	1.3	0.4	2.1	1	1.2	0.7	3	0.8	1.4	1.1	1.5
Oow	1.2	0.5	0.6	1	1.2	0.7	1.5	0.5	0.9	1.5	1.7

Rank 5

f_{obs}/f_{exp} (scale: 0 1 2 3 4 5 6)

	Empty	Hetatm	Meta	Xot	Car	Nam	Nbas	Oc	Oh	Oox	Oow
Xot	0.7	0.4	0.9	1.1	1.1	1	0.9	1	0.9	0.9	0.9
Car	1.2	0.5	0.9	1.2	1.4	0.6	1	0.9	1.2	1.3	1
Nam	0.6	0.4	1.3	0.9	0.9	1.5	1.1	0.8	1.1	0.9	1
Nbas	5.4	0.4	0.9	0.8	0.8	0.7	1.5	0.9	1.4	2.6	1.4
Oc	0.6	0.4	0.8	0.6	0.8	1.1	0.8	1.5	0.9	0.9	1
Oh	1.2	0.4	1	1	1	0.9	1.2	1	1.3	1.3	1.1
Oox	2.4	0.4	1.8	0.8	0.7	0.9	2	0.9	1.5	1.5	1.5
Oow	0.3	0.4	0.7	1	0.8	0.7	1.3	0.6	0.9	1.2	1.9

Rank 6

f_{obs}/f_{exp} (scale: 0 1 2 3 4 5 6)

	Empty	Hetatm	Meta	Xot	Car	Nam	Nbas	Oc	Oh	Oox	Oow
Xot	0.4	0.4	0.9	1	1	1.1	0.9	1.1	1	0.9	1
Car	0.9	0.6	0.8	1	1.5	0.7	1.4	1	1.1	1.2	1
Nam	0.4	0.4	1.6	1	0.7	1.4	0.8	1	0.9	0.8	1.1
Nbas	5.3	0.6	0.7	0.8	1.5	0.7	1.3	0.9	1.3	3.3	1.5
Oc	0.5	0.3	0.5	1.2	0.7	0.9	0.7	1	0.9	0.8	0.7
Oh	1.1	0.6	0.6	1	1.1	0.9	1.2	1	1.1	1.5	1
Oox	1.6	0.4	2	0.9	1.1	0.8	2.9	0.9	1.4	1.1	1.5
Oow	1.7	0.5	0.6	1	1.1	0.7	1.5	0.6	0.9	1.5	1.8

Rank 6

f_{obs}/f_{exp} (scale: 0 1 2 3 4 5 6)

	Empty	Hetatm	Meta	Xot	Car	Nam	Nbas	Oc	Oh	Oox	Oow
Xot	0.7	0.4	0.9	1.2	1.1	1	1	1	1	0.9	0.9
Car	1.3	0.4	0.8	1.2	1.4	0.7	1	1	1.2	1.3	1
Nam	0.7	0.3	1.1	0.9	0.9	1.3	1	1	1.1	0.9	1
Nbas	5	0.4	0.9	0.8	0.8	0.7	1.4	0.9	1.3	2.3	1.3
Oc	0.7	0.4	0.9	0.6	0.7	1.2	0.8	1.5	0.9	0.9	1
Oh	1.2	0.4	0.9	1	1	1	1.2	1	1.2	1.2	1.1
Oox	2.6	0.4	1.8	0.8	0.7	0.9	1.8	0.9	1.4	1.5	1.4
Oow	0.4	0.4	0.8	0.9	0.7	0.7	1.3	0.6	0.9	1.3	2

Rank 7

f_{obs}/f_{exp} (scale: 0 1 2 3 4 5 6)

	Empty	Hetatm	Meta	Xot	Car	Nam	Nbas	Oc	Oh	Oox	Oow
Xot	0.5	0.4	0.9	1	1.1	1	0.9	1.1	1	0.9	0.9
Car	0.9	0.6	0.8	1	1.5	0.7	1.3	1	1.1	1.2	1
Nam	0.5	0.4	1.6	0.8	0.7	2.1	0.8	0.9	1	0.8	1.1
Nbas	3.8	0.6	0.8	0.9	1.4	0.7	1.3	1	1.2	3	1.5
Oc	0.8	0.3	0.6	1.2	0.7	0.7	0.7	1	0.9	0.8	0.7
Oh	1.3	0.5	0.6	1	1.1	0.9	1.2	1.1	1.2	1.5	1.1
Oox	1.6	0.4	1.9	0.9	1.1	0.8	2.8	1	1.4	1.1	1.5
Oow	2.2	0.5	0.6	1	1.1	0.7	1.5	0.7	1	1.5	1.9

Rank 7

f_{obs}/f_{exp} (scale: 0 1 2 3 4 5 6)

	Empty	Hetatm	Meta	Xot	Car	Nam	Nbas	Oc	Oh	Oox	Oow
Xot	0.8	0.4	0.9	1.1	1.1	1	1	1	1	0.9	0.9
Car	1.3	0.4	0.8	1.2	1.3	0.8	1	1	1.1	1.2	0.9
Nam	0.7	0.4	1	0.9	1	1.2	1	1	1	0.9	1
Nbas	4.7	0.4	0.9	0.8	0.7	0.8	1.4	1	1.3	2.1	1.3
Oc	0.7	0.4	1	0.7	0.9	1.1	0.9	1.4	0.9	0.9	1
Oh	1.2	0.4	0.9	1	1	1.1	1.1	1	1.2	1.2	1
Oox	2.7	0.3	1.4	0.8	0.7	0.9	1.7	1	1.3	1.5	1.3
Oow	0.4	0.4	0.9	0.7	0.6	0.8	1.2	0.7	0.9	1.4	2.1

Rank 8

f_{obs}/f_{exp} (scale: 0 1 2 3 4 5 6)

	Empty	Hetatm	Meta	Xot	Car	Nam	Nbas	Oc	Oh	Oox	Oow
Xot	0.7	0.4	0.9	1	1.1	1	0.9	1	1	0.9	0.9
Car	0.9	0.6	0.8	1	1.5	0.7	1.3	1.1	1.1	1.2	1
Nam	0.6	0.4	1.6	0.8	0.7	2.1	0.8	0.9	1	0.9	1
Nbas	2.5	0.6	0.9	0.8	1.3	0.8	1.3	1	1.2	2.9	1.5
Oc	1	0.3	0.6	1.2	0.7	0.6	0.7	1.1	0.9	0.8	0.7
Oh	1.2	0.5	0.8	1	1	0.9	1.2	1.1	1.2	1.5	1.1
Oox	1.5	0.4	1.9	0.9	1	0.8	2.8	1.1	1.4	1.2	1.5
Oow	2.6	0.5	0.7	1	1	0.7	1.5	0.8	1	1.6	2

Rank 8

f_{obs}/f_{exp} (scale: 0 1 2 3 4 5 6)

	Empty	Hetatm	Meta	Xot	Car	Nam	Nbas	Oc	Oh	Oox	Oow
Xot	0.8	0.4	0.9	1.1	1	1.1	1	1	1	0.9	0.9
Car	1.2	0.4	0.9	1.1	1.2	0.9	0.9	1.1	1.1	1.2	0.9
Nam	0.7	0.4	1	1	1.2	1.1	1	1	1	0.9	1
Nbas	4.3	0.3	0.9	0.7	0.6	0.8	1.3	1	1.2	1.9	1.2
Oc	0.8	0.4	1.1	0.9	1	1	1	1.3	1	1	1
Oh	1.2	0.4	0.9	1	0.9	1.1	1.1	1	1.2	1.1	1
Oox	2.7	0.3	1.3	0.8	0.7	0.9	1.5	1	1.2	1.5	1.2
Oow	0.4	0.4	1	0.6	0.5	0.9	1.2	0.8	0.9	1.4	2

Rank 9

f_{obs}/f_{exp} (scale: 0 1 2 3 4 5 6)

	Empty	Hetatm	Meta	Xot	Car	Nam	Nbas	Oc	Oh	Oox	Oow
Xot	0.8	0.4	0.9	1	1.1	1	0.9	1	1	0.9	0.9
Car	0.8	0.6	0.8	1	1.5	0.8	1.3	1.1	1.1	1.2	1
Nam	0.8	0.4	1.5	0.9	0.7	1.8	0.8	0.8	1	0.8	1.1
Nbas	1.6	0.6	0.8	0.8	1.3	0.8	1.3	1.1	1.2	2.8	1.5
Oc	1	0.3	0.6	1.2	0.8	0.7	0.8	1.1	0.9	0.8	0.8
Oh	1.1	0.5	0.8	1	1	1	1.2	1.2	1.2	1.4	1.1
Oox	1.2	0.4	1.9	0.9	1	0.9	2.7	1.1	1.3	1.2	1.5
Oow	2.4	0.5	0.8	0.9	1	0.8	1.4	0.8	1	1.6	2.1

Rank 9

f_{obs}/f_{exp} (scale: 0 1 2 3 4 5 6)

	Empty	Hetatm	Meta	Xot	Car	Nam	Nbas	Oc	Oh	Oox	Oow
Xot	0.8	0.4	0.9	1.1	1	1.1	1	1	1	0.9	0.9
Car	1.2	0.4	0.9	1.1	1.1	0.9	0.9	1.1	1.1	1.2	0.9
Nam	0.7	0.4	1	1	1.2	1	1	1	1.1	0.9	1
Nbas	3.9	0.3	0.8	0.7	0.6	0.8	1.2	1	1.2	1.7	1.2
Oc	0.8	0.4	1.1	1	1.1	0.9	1	1.2	1.1	1	1
Oh	1.2	0.4	0.9	1	0.9	1.1	1.1	1	1.2	1.1	1
Oox	2.7	0.3	1.1	0.8	0.7	0.9	1.5	1	1.2	1.4	1.2
Oow	0.6	0.4	1	0.6	0.5	0.9	1.2	0.9	0.9	1.4	2

Rank 10

f_{obs}/f_{exp} (scale: 0 1 2 3 4 5 6)

	Empty	Hetatm	Meta	Xot	Car	Nam	Nbas	Oc	Oh	Oox	Oow
Xot	0.9	0.4	0.9	1	1.1	1	0.9	1	1	0.9	0.9
Car	0.8	0.6	0.8	1	1.4	0.8	1.3	1.1	1.1	1.2	1
Nam	0.8	0.4	1.4	1	0.7	1.6	0.9	0.8	1	0.9	1.1
Nbas	1.3	0.6	0.9	0.8	1.2	0.9	1.3	1.1	1.2	2.7	1.5
Oc	0.9	0.3	0.7	1.1	0.8	0.7	0.8	1.2	1	0.9	0.8
Oh	1	0.5	0.9	0.9	1	1	1.2	1.2	1.2	1.4	1.1
Oox	1	0.4	1.7	0.9	1	0.9	2.6	1.2	1.3	1.2	1.5
Oow	2.1	0.5	0.8	0.9	0.9	0.8	1.4	0.9	1	1.6	2.1

Rank 10

f_{obs}/f_{exp} (scale: 0 1 2 3 4 5 6)

	Empty	Hetatm	Meta	Xot	Car	Nam	Nbas	Oc	Oh	Oox	Oow
Xot	0.9	0.4	0.9	1.1	1	1.1	1	1	1	0.9	0.9
Car	1.1	0.4	1	1	1	1	0.9	1.1	1.1	1.1	0.9
Nam	0.8	0.4	1	1	1.3	1	1.1	1	1.1	0.9	1
Nbas	2.9	0.3	0.8	0.7	0.6	0.8	1.2	1	1.1	1.5	1.1
Oc	0.8	0.4	1.1	1	1.1	0.9	1.1	1.1	1.1	1	1
Oh	1.2	0.4	0.9	1	0.9	1	1.1	1	1.2	1.1	1
Oox	2.2	0.4	1	0.8	0.7	0.9	1.4	1	1.1	1.4	1.1
Oow	1	0.4	1.1	0.6	0.5	0.9	1.2	0.9	1	1.3	1.9

Supporting information 6. Influence of the number of neighbors k on the FS_{atom}^{k} score distribution. Range of the FS_{atom}^{k} scores calculated for the set of native structures (see manuscript Figure 5, no water molecules) when the number of neighbor k varies. The neighbor list is collected with the *primary contact* filter applied.

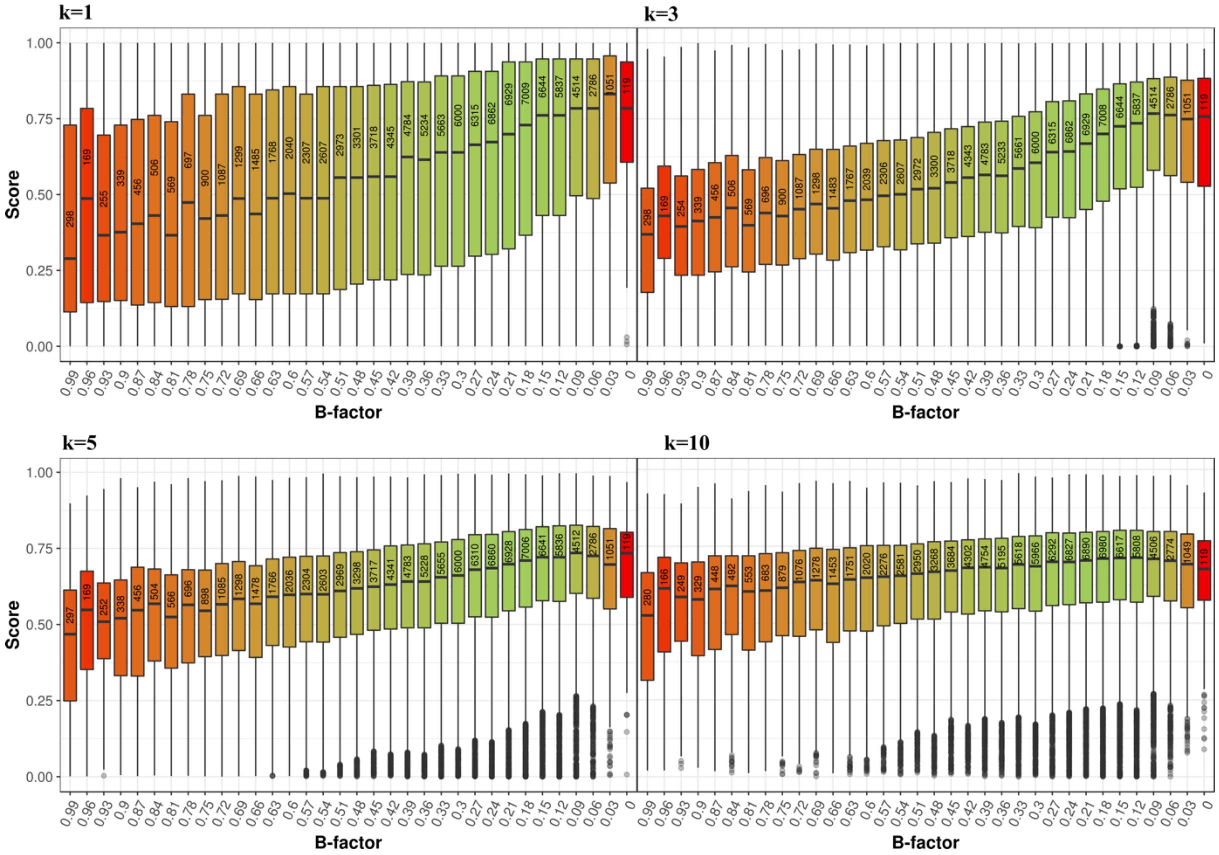

Supporting information 7. Correspondence analyses for the 10 origins. The neighbor list is collected without (left-hand panels) or with (right-hand panels) the *primary contact* filter activated.

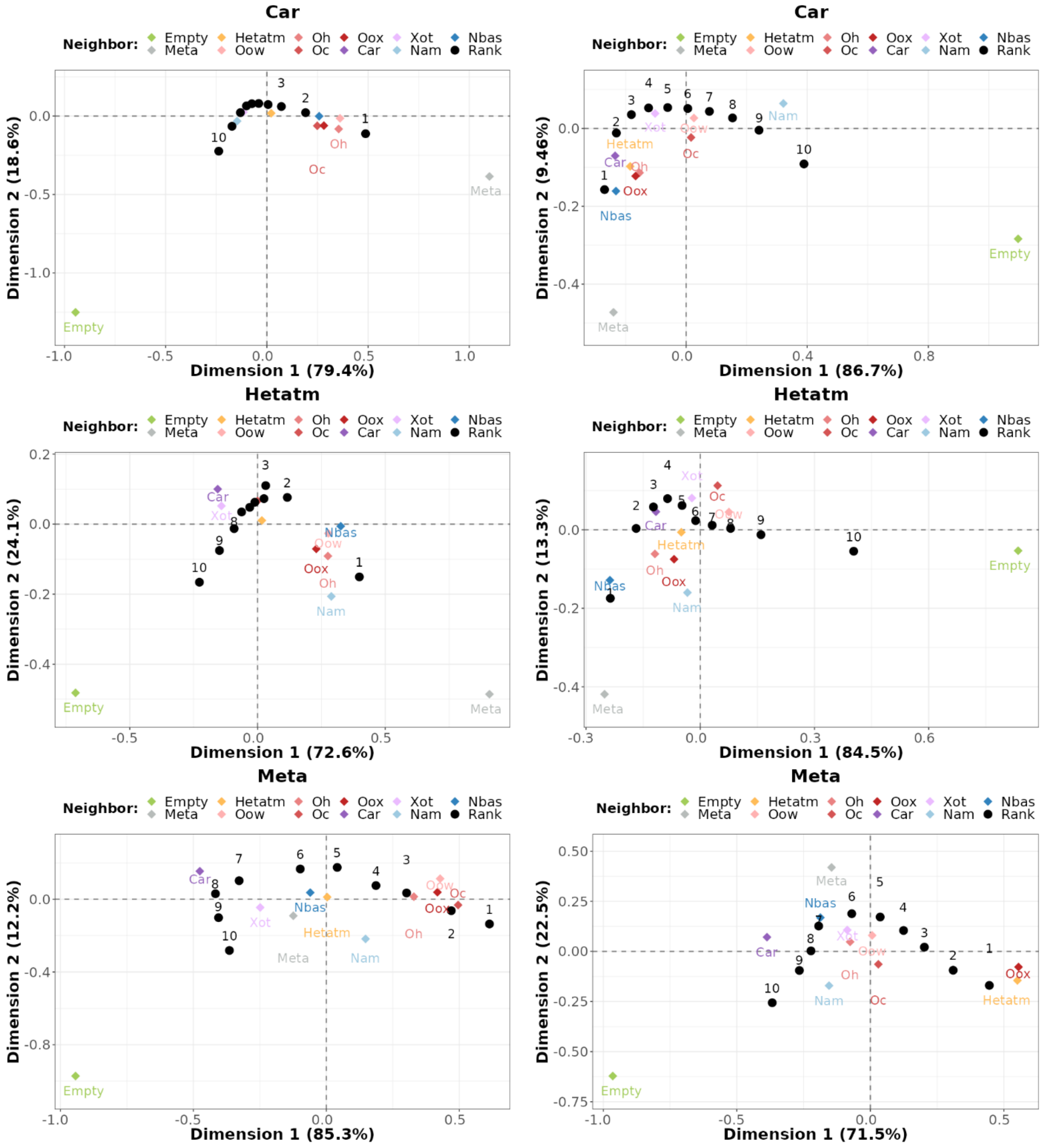

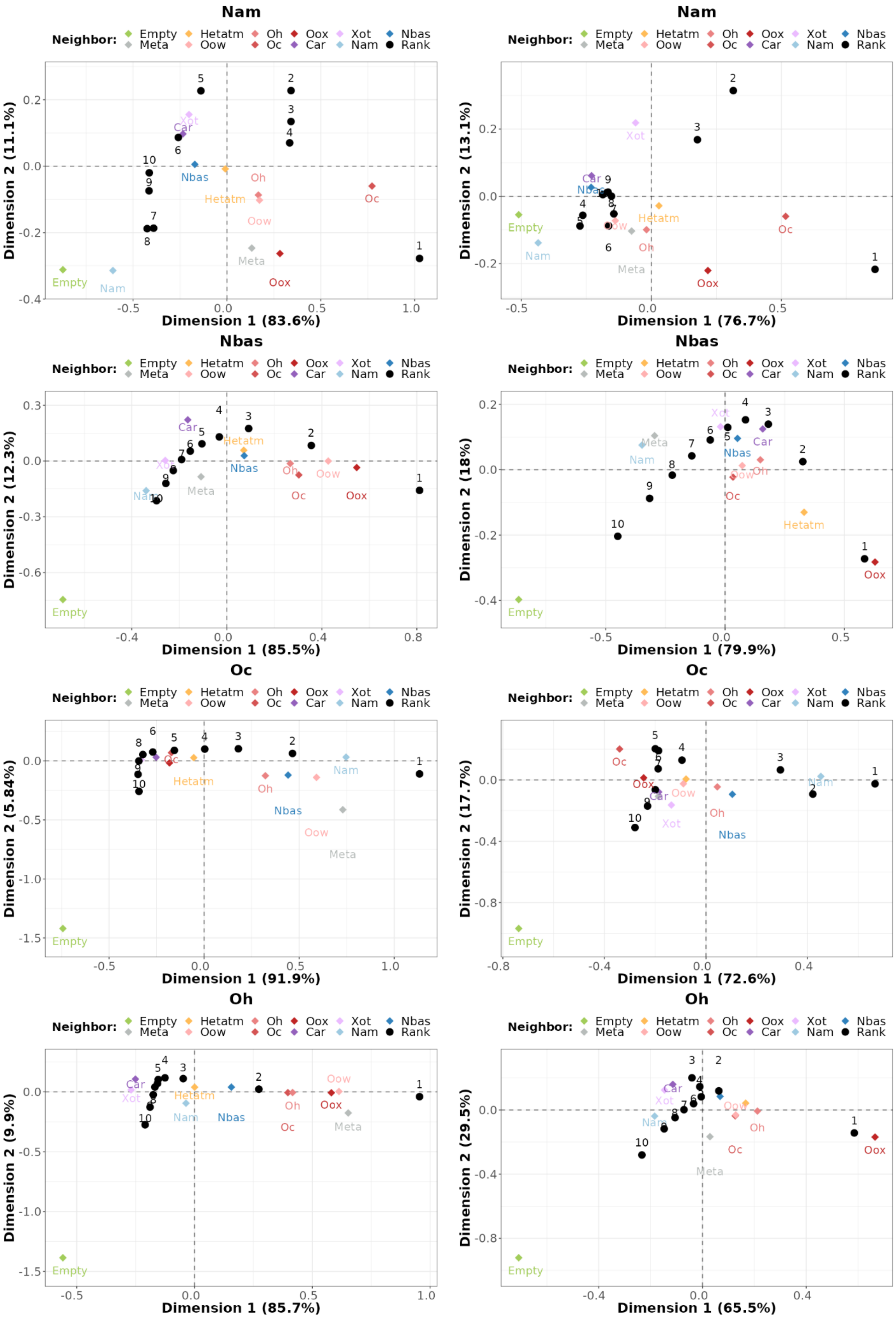
Nam
Neighbor: Empty Meta Hetatm Oow Oh Oc Oox Car Xot Nam Nbas Rank
Dimension 1 (83.6%)
Dimension 2 (11.1%)
Nam
Neighbor: Empty Meta Hetatm Oow Oh Oc Oox Car Xot Nam Nbas Rank
Dimension 1 (76.7%)
Dimension 2 (13.1%)
Nbas
Neighbor: Empty Meta Hetatm Oow Oh Oc Oox Car Xot Nam Nbas Rank
Dimension 1 (85.5%)
Dimension 2 (12.3%)
Nbas
Neighbor: Empty Meta Hetatm Oow Oh Oc Oox Car Xot Nam Nbas Rank
Dimension 1 (79.9%)
Dimension 2 (18%)
Oc
Neighbor: Empty Meta Hetatm Oow Oh Oc Oox Car Xot Nam Nbas Rank
Dimension 1 (91.9%)
Dimension 2 (5.84%)
Oc
Neighbor: Empty Meta Hetatm Oow Oh Oc Oox Car Xot Nam Nbas Rank
Dimension 1 (72.6%)
Dimension 2 (17.7%)
Oh
Neighbor: Empty Meta Hetatm Oow Oh Oc Oox Car Xot Nam Nbas Rank
Dimension 1 (85.7%)
Dimension 2 (9.9%)
Oh
Neighbor: Empty Meta Hetatm Oow Oh Oc Oox Car Xot Nam Nbas Rank
Dimension 1 (65.5%)
Dimension 2 (29.5%)

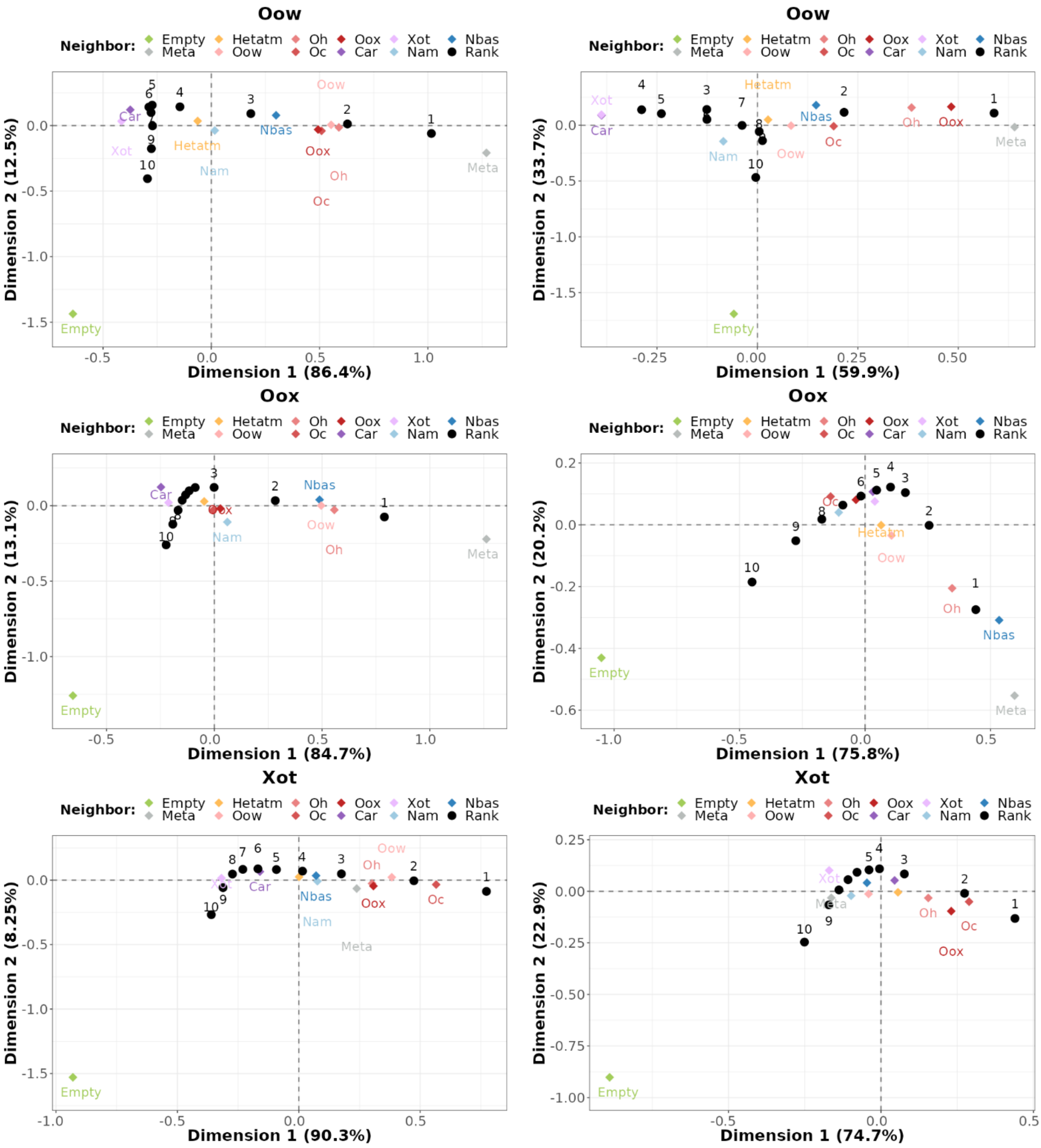
Oow
Neighbor: Empty Meta Hetatm Oow Oh Oc Oox Car Xot Nam Nbas Rank
Dimension 1 (86.4%)
Dimension 2 (12.5%)
Oow
Dimension 1 (59.9%)
Dimension 2 (33.7%)
Oox
Dimension 1 (84.7%)
Dimension 2 (13.1%)
Oox
Dimension 1 (75.8%)
Dimension 2 (20.2%)
Xot
Dimension 1 (90.3%)
Dimension 2 (8.25%)
Xot
Dimension 1 (74.7%)
Dimension 2 (22.9%)
